# Hydrophobic liquid electrolyte interphases for efficient aqueous zinc batteries

**DOI:** 10.1038/s41565-026-02187-0

**Published:** 2026-06-01

**Authors:** Guanjie Li, Shilin Zhang, Jodie A. Yuwono, Xinyu Li, Javen Qinfeng Shi, Chunsheng Wang, Zaiping Guo

**Affiliations:** 1 School of Chemical Engineering, College of Engineering and Information Technology, Adelaide University, Adelaide, South Australia Australia; 2 Responsible AI Research Centre, Australian Institute for Machine Learning, Adelaide University, Adelaide, South Australia Australia; 3 Department of Chemical and Biomolecular Engineering, University of Maryland, College Park, MD USA

**Keywords:** Batteries, Electrochemistry, Energy storage, Materials for energy and catalysis

## Abstract

Expanding the electrochemical stability window of aqueous electrolyte solutions is a viable strategy to improve battery performance. Using water-in-salt aqueous electrolyte solutions, the solid electrolyte interphase formed on the negative electrode enables an electrochemical stability window up to 3.0 V, but this often reduces ionic conductivity and increases costs. Here, to circumvent these issues, we report the use of hydrophobic and electrode-philic ether-based additives in 3-molal aqueous zinc trifluoromethanesulfonate electrolyte solutions. These additives, characterized by a weak Zn-ion solvation capability, are soluble in the aqueous electrolyte solution at low concentrations (below 2 mol%). They can be adsorbed on both positive and negative electrode surfaces, inhibiting Zn dendrite growth, forming a liquid electrolyte interphase that extends the electrochemical stability window to 3.08 V, enabling high bulk ionic conductivity (about 54 mS cm^−1^ at 25 °C) and ensuring the non-flammability of the aqueous electrolyte solution. This nanoengineered electrolyte approach enables a Zn||NaV_3_O_8_ single-layer pouch cell to operate 500 stable cycles (average Coulombic efficiency of 99.95%) with a specific discharge capacity retention of 80% at 500 mA g^−1^ and 25 °C with a calculated initial specific energy of 132 Wh kg^−1^ (based on the mass of the negative and positive electrode active materials).

## Abstract (web summary)

A hydrophobic, electrode‑philic ether additive self‑assembles into a nanoscale liquid electrolyte interphase that decouples interfacial and bulk electrolyte behaviour, suppresses water‑driven parasitic reactions and enables efficient aqueous zinc batteries.

## Main

Aqueous Zn batteries (AZBs) are a cost-effective, safe and environmentally friendly technology for large-scale energy storage^1–3^. However, their widespread deployment is hindered by the narrow electrochemical stability window (ESW) of the water solvent (limited to 1.23 V, the theoretical thermodynamic potential versus the reversible hydrogen electrode) and limited cycle life caused by Zn dendrite growth at the negative electrode. Since the Zn redox potential is lower than the reduction potential of H_2_O, during Zn plating and stripping, the parasitic hydrogen evolution reaction (HER) continuously generates hydrogen gas, increasing the internal cell pressure and electrolyte consumption, and forming a hydrophilic interphase (for example, containing Zn(OH)_2_ and Zn_4_SO_4_(OH)_6_·*n*H_2_O), which promotes Zn dendrite growth. Oxygen evolution reaction (OER) at high cell potentials limits the use of positive electrodes, capping the energy density of AZBs. To address these challenges, expanding the ESW of aqueous electrolytes (AEs) beyond the operating cell potential remains a key focus in battery research^4–6^.

**Fig. 1:**
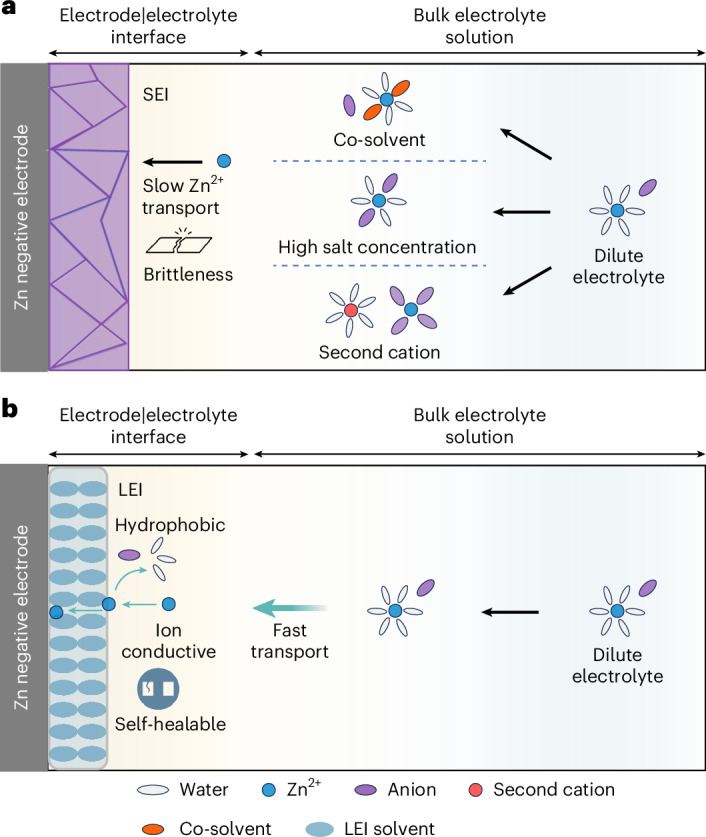
Comparison of bulk electrolyte engineering and interfacial LEI strategy for ESW expansion. **a**,**b**, Schematic of conventional bulk electrolyte engineering strategies (**a**) and the proposed LEI strategy for expanding the ESW (**b**).

The formation of a passivation interphase at the electrode|electrolyte interface, like the solid electrolyte interphase (SEI) at the cell’s negative electrode, which allows positively charged ionic species to reach the electrode surface and inhibiting water from reaching it, is essential to extend the electrolyte solution’s ESW. The formation of passivation interphases requires more salt-derived anions and less water on the electrode surface for redox reactions^7,8^. However, the complexity and variability of electrolyte solutions at the electrode interface, along with the lack of reliable engineering approaches for precise interface control, have led researchers to focus on modifying the bulk properties of electrolyte solutions to regulate interfacial behaviours. These bulk electrolyte engineering strategies aim to form a stable and ionically conductive SEI (Fig. 1a) via (1) adding organic solvents with higher Zn^2+^ solvation energies than water; (2) increasing the zinc salt concentration in the electrolyte solution, enabling the anion to enter the Zn^2+^ solvation shell; or (3) introducing supporting cations that bind to water more strongly than the active cation, enabling the anion to enter the active cation solvation sheath^9–13^.

However, these approaches require either a high concentration of salts or organic solvents in the electrolyte solution, which compromises Zn^2+^ ion conductivity and the non-flammability of the electrolyte solutions^14–16^. Strongly coordinating organic–Zn^2+^ clusters also increase the desolvation barrier and make the electrode kinetics sluggish^17^. Liquid electrolyte solutions that form anion-derived SEI on the electrode surface generally have a low bulk ionic conductivity due to the low coordination number of water molecules in the active cation solvation shell. Even when the SEI is formed using water-in-salt electrolyte solutions (which are highly concentrated aqueous solutions with salt concentrations generally of >5 molal), their ESW remains limited to 3.0 V because H_2_ evolution from water reduction on the Zn electrode can mechanically damage the rigid ceramic SEI. In addition, since the Zn^2+^ conductivity in the SEI is several magnitude lower than that in the bulk liquid electrolyte solution, the SEI brings additional interface impedance^15,18,19^. The rigid and brittle ceramic SEI also cannot accommodate the volume changes in negative metal electrodes during battery operation, and it generally cracks and reforms, which detrimentally affects the ESW of the electrolyte solution and the cycle life of the battery.

A promising strategy to overcome the SEI challenges is to form a liquid electrolyte interphase (LEI) that has the same functions as the SEI but is not affected by mechanical degradation (Fig. 1b). Unlike the SEI, which is formed by the reductive decomposition of anions in the Zn solvation shell, the LEI can be formed by the adsorption of electrode-philic and hydrophobic additives on the electrode surface, minimizing the interface effect on the electrolyte bulk properties and stabilizing the interface thermodynamically through its electrochemical inertness and hydrophobic nature. The decoupling of the interfacial and bulk properties of the electrolyte solution enables nanoengineering at the electrode|electrolyte interface, blocking water from reaching the electrode surface, and preserving the high ionic conductivity of the bulk electrolyte by fully solvating the Zn^2+^ cation. Although the SEI is generally brittle and exhibits high interfacial impedance, the LEI, as a liquid-phase interphase formed by spontaneously assembled organic species, enables self-healing through dynamic reorganization and maintains fast ion transport (Fig. 1b). Also, the hydrophobic LEI promotes the removal of coordinated water molecules, enabling a faster desolvation process.

Here we report the formation of an LEI at the zinc metal electrode|AE solution interface by introducing an ether-based hydrophobic organic additive, soluble at a low concentration of 1.8 mol%, which is also electrode-philic and shows optimized solvating ability. The hydrophobic LEI formed on the electrode surface is a hydrophobic liquid phase, distinct from the bulk AE solution phase, which enables the expansion of the ESW up to 3.08 V (–1.27 V to 1.81 V versus Ag|AgCl). This LEI characteristics enable the long-term cycling stability of Zn||Zn coin cells after 2,000 h at a depth of discharge (DoD_Zn_) of 60% and an average Coulombic efficiency (CE) of 99.83% over 2,500 cycles for Zn||Zn coin cells cycled at 1 mA cm^−2^ and 25 °C. In the Zn||NaV_3_O_8_ single-layer pouch cell configuration (with negative-to-positive (N/P) and electrolyte-to-capacity (E/C) ratios of 1.67 and 6 g Ah^−1^, respectively), the LEI enables an initial specific energy of 132 Wh kg^−1^ (based on the mass of the negative and positive electrode active materials) and a lifespan of 500 stable cycles (average CE of 99.95%) with a specific discharge capacity retention of 80% at 500 mA g^−1^ and 25 °C.

## Results

### LEI design

**Fig. 2:**
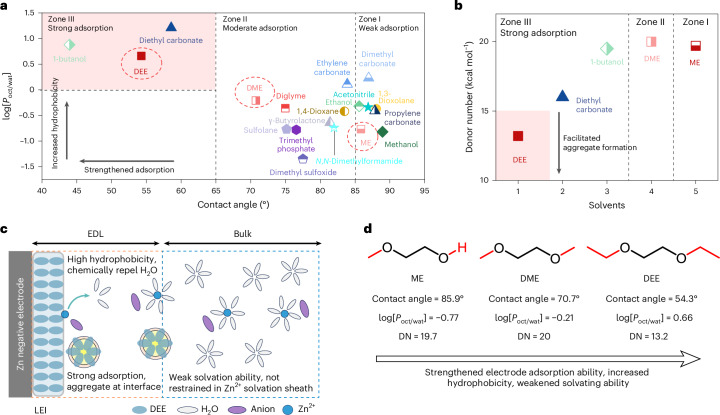
Hydrophobic LEI design principle. **a**, Solvent diagram of log[*P*_oct/wat_] (octanol–water partition coefficient) versus contact angle. Solvents in zone III (highlighted in pink) can adsorb onto the Zn electrode and expel water from the Zn surface due to their high hydrophobicity. Solvents in zone II show moderate adsorption ability and hydrophobicity, leading to partial water exclusion from the Zn surface, whereas solvents in zone I exhibit weak adsorption and low hydrophobicity and, thus, cannot displace water or form a stable LEI layer on the Zn electrode. **b**, Donor number of solvents in zones I–III (values are taken from refs. ^24–27^, measured at 25 °C). **c**, Schematic illustrating the role of three key parameters of organic solvents, namely, strong electrode adsorption ability, high hydrophobicity and weak solvation ability, in facilitating LEI layer formation. The yellow-filled, orange-bordered circle denotes a DEE cluster composed of aggregated DEE molecules (blue). **d**, Chemical structures of three ether molecules selected to investigate the LEI design. DN, donor number. Source data

To formulate an effective LEI, we screen a range of organic molecules that should be (1) strongly adsorbed on the Zn metal electrode surface to enable spontaneous aggregation into a liquid interlayer at the Zn|AE interface, and (2) hydrophobic enough to give the liquid interlayer water-repellent properties. The adsorption ability can be indicated by the contact angle between the electrolyte solutions and the Zn metal electrode^20–22^. A smaller contact angle suggests stronger adsorption, as the adsorbed organic additive molecules on the Zn surface reduce surface tension at the solid|liquid interface (Supplementary Note 1). Additionally, the logarithm (log) of the partition coefficient between an octanol (non-aqueous) phase and a water (aqueous) phase (log[*P*_oct/wat_]) acts as a descriptor of hydrophobicity^23^. Considering both adsorption and hydrophobicity factors within a range of organic molecules (Fig. 2a and Supplementary Table 1), we observed that most of them exhibit a weak-to-moderate adsorption ability on the Zn surface (contact angle with Zn, >65°) with weak-to-moderate hydrophobicity (log[*P*_oct/wat_] < 0). These organic additives fall within zones I and II shown in Fig. 2a. Only a few organic molecules (those in zone III (Fig. 2a)) exhibit both strong adsorption tendencies and high hydrophobicity, meeting the primary requirement for LEI formation. Another essential criterion is a weak solvation ability, characterized by a low donor number value of the organic additive^24–27^, which facilitates the formation of a compact, widely distributed LEI. This feature guarantees that the organic additive is not confined within the Zn^2+^ solvation sheath and remains soluble in the AE solution, aggregating into large organic clusters driven by their hydrophobicity, forming a micelle structure (Fig. 2b and Supplementary Note 1). This micelle further adsorbs onto the Zn surface, providing a continuous, dense organic-molecular structure within the LEI layer (Fig. 2c).

Applying these criteria to organic molecules in zone III (Fig. 2a), 1,2-diethoxyethane (DEE) emerges as a suitable organic additive for LEI formation. The evaluation of the CE of Zn||Cu asymmetric coin cells indicates that cells using a DEE-containing AE solution exhibit a higher CE compared with those using other organic molecules (Supplementary Figs. 1–9, Supplementary Note 1). We also observed that as the molecular size increased from 2-methoxyethanol (ME) to 1,2-dimethoxyethane (DME) to DEE, their adsorption ability strengthened, hydrophobicity increased and solvating capability weakened (Fig. 2d); this trend suggests an enhanced capability to form an LEI.

### LEI in AEs with DEE additive

**Fig. 3:**
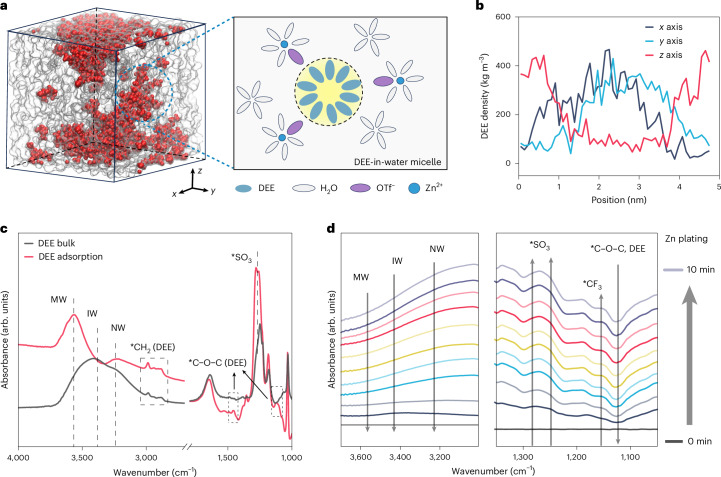
LEI formation on the Zn electrode. **a**, Snapshot of the 3-m Zn(OTf)_2_ AE solution containing DEE additive (1.8 mol%) obtained from MD simulations (left) and schematic of the DEE micelle structure (right). In the MD snapshot (left), DEE molecules are highlighted in red and water molecules are represented as transparent. The yellow-filled, dotted circle denotes a DEE cluster, where aggregated DEE molecules (blue) form a distinct domain, separated from the surrounding water clusters. **b**, Density profiles of DEE molecules across the *x*, *y* and *z* axes in the MD simulation box for the 3-m Zn(OTf)_2_ AE solution containing the DEE additive (1.8 mol%). The clustered distribution of DEE molecules in the electrolyte solution indicates the formation of DEE-in-water micelles. **c**, ATR-SEIRAS spectra of equilibrium adsorption species on Zn electrodes in the DEE-containing electrolyte solution (1.8 mol%), suggesting the formation of DEE-containing LEI on the Zn electrodes. NW, network water; IW, intermediate water; MW, multimer water. **d**, In situ ATR-SEIRAS spectra of adsorption species during Zn plating in the DEE-containing electrolyte solution (1.8 mol%), indicating changes in the surface concentration of H_2_O and OTf^−^ anion. The negative shift of the O–H stretching vibration indicates that DEE-containing LEI repels H_2_O from the Zn electrode surface, whereas the positive shift in *SO_3_ and *CF_3_ stretching vibrations suggests OTf^−^ anions are drawn to the Zn electrode surface. Source data

The 3 mol kg^−1^ (3-m) organic-additive-containing AE solutions prepared by dissolving 3 mol of zinc trifluoromethanesulfonate (Zn(OTf)_2_) salt in H_2_O–ether solvent mixtures (using either ME, DME or DEE as organic additives). The concentration of the organic additives in H_2_O ranges from 1.8 to 16.2 mol%. The 3-m Zn(OTf)_2_ dissolved in pure water is denoted as the baseline AE. The structures and properties of these electrolyte solutions were examined through spectroscopic measurements and theoretical simulations. Raman spectra revealed that the C–O–C symmetric stretching of DEE remains unchanged across DEE-containing electrolytes (1.8–16.2 mol%), indicating that DEE does not solvate Zn^2+^ but remains as a cation-uncoordinated species outside the solvation sheath (Supplementary Fig. 10). Moreover, its weak solvating ability and hydrophobicity promote its self-aggregation into micelle-like clusters. Molecular dynamics (MD) simulations confirm the formation of such micelle structures (Fig. 3a,b), which is distinct from the homogeneous distribution of H_2_O–ether binary solvents observed in AE solutions containing ME and DME additives (Supplementary Fig. 11). Small-angle X-ray scattering spectroscopy (SAXS) measurements show a widened peak at 0.42 Å^−1^ in the 1.8-mol%-DEE-containing electrolyte solution, suggesting the presence of micelle-like clusters (Supplementary Fig. 12). Due to the presence of these micelles, the water hydrogen network within the DEE-containing electrolyte solutions is disrupted at a high DEE concentration (for example, 16.2 mol%)^28–30^, as demonstrated by the notable blueshift and reduced intensity of the O−H stretching vibration of water (2,700–3,700 cm^−1^) observed in the Fourier transform infrared (FTIR) spectra (Supplementary Fig. 13). The deconvolution analysis quantitatively reveals that more than 12.1% of network water transforms into intermediate water and multimer water (poorly connected water)^11,31^ on increased concentrations of DEE (Supplementary Figs. 14–16). However, at a low DEE concentration, the continuous Zn–H_2_O conduction channels maintain high ionic conductivity.

The DEE micelles further adsorb onto the Zn metal electrode surface, forming an LEI layer (DEE-LEI) that is phase separated from the bulk electrolyte solution, as also suggested by the aggregation of DEE clusters on the Zn electrode observed in the MD simulations (Supplementary Fig. 17). The DEE-LEI has also been observed via attenuated total reflection–surface enhanced infrared absorption spectroscopy (ATR-SEIRAS; Supplementary Fig. 18) measurements, which can amplify the infrared signals of interfacial species within ~5 nm of the electrode surface, thereby enabling the identification of surface-adsorbed species^32^. The *v*_s_(C–H) peaks (2,800–3,000 cm^−1^, 1,455 cm^−1^) and the newly emerged *v*_s_(C–O–C) peak at 1,118 cm^−1^ indicate the spontaneous adsorption of the DEE-LEI layer on the Zn surface (Fig. 3c). Continuous spectral monitoring reveals that the DEE-LEI layer forms rapidly on the electrolyte solution contact with the Zn metal electrode and is completed within about 3 min, before the formation of any SEI or other by-products on the Zn metal surface (Supplementary Figs. 19 and 20). The DEE-LEI layer interrupts the water network and repels water molecules from the Zn|electrolyte interface, as evidenced by the increased multimer water content, but reduced intermediate water and network water signals. By contrast, the intensity of *v*_s_(C–H) and *v*_s_(C–O–C) peaks is low in electrolyte solutions containing ME and DME additives, consistent with their limited adsorption ability on the Zn surface (Supplementary Fig. 21). Near-ambient-pressure (NAP) X-ray photoelectron spectroscopy (XPS) measurements confirm the strong adsorption of DEE-LEI on the Zn surface, showing a higher S(C–O–C_ether_)/S(H_2_O_ads_) ratio of 3.45 compared with those in electrolyte solutions containing ME (0.77) and DME (1.27) (Supplementary Fig. 22 and Supplementary Table 2). This observation is further supported by the higher adsorption energy of DEE on Zn(101) and (002) facets and a lower capacitance of the electric double layer (*C*_EDL_) of the Zn electrode (Supplementary Figs. 23–25) compared with those in ME and DME electrolytes. The strong adsorption ensures robust, stable attachment of DEE-LEI to the Zn metal electrode surface throughout the Zn plating process^33^. Quartz crystal microbalance (QCM) analysis reveals that the adsorbed amount of DEE is ~0.0132 mg cm^−2^ on the Zn electrode (Supplementary Fig. 26). In comparison, the DEE dosage in the electrolyte is ~3 mg cm^−2^, based on the addition of 50 µl cm^−2^ electrolyte per Zn||Zn coin cell. This dosage is several orders of magnitude higher than that required for saturated adsorption, suggesting that the electrolyte provides a sufficient DEE reservoir to sustain a fully developed LEI layer on the Zn electrode, even as the surface area increases during Zn deposition.

In situ ATR-SEIRAS reveals that no water reaches the Zn surface during Zn deposition in the DEE-containing electrolyte due to the hydrophobic nature of the LEI (Fig. 3d). However, DEE-LEI allows the transport of OTf^−^ anions to the Zn surface, driven by the strong bonding between –CF_3_ (OTf^−^ anions) and hydrophobic tails –CH_2_CH_3_ (DEE molecules). By contrast, the electrolyte solutions with ME and DME additives lack water-repellent capabilities (weak adsorption and hydrophobicity), which allows notable amounts of network water and intermediate water to be drawn to the Zn surface (Supplementary Figs. 27 and 28). Linear sweep voltammetry measurements reveal that DEE exhibits good reductive stability even at −3.0 V versus Ag|AgCl, indicating that DEE remains electrochemically stable within the operational potential window of Zn electrodes (Supplementary Fig. 29).

### Fundamental electrochemical properties of AE solutions with ether additive

**Fig. 4:**
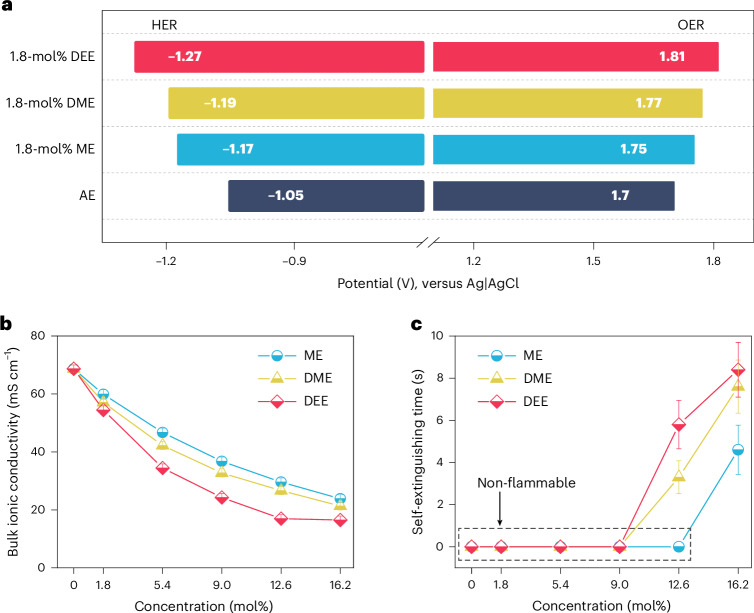
Fundamental electrochemical properties of electrolyte solutions with ether additives. **a**, ESWs of AE-, 1.8-mol%-ME-, 1.8-mol%-DME- and 1.8-mol%-DEE-containing electrolyte solutions, determined by coupled linear sweep voltammetry and differential electrochemical mass spectrometry measurements (Supplementary Fig. 30 shows extended data). **b**, Bulk ionic conductivities of electrolytes with different concentrations of organic solvents at 25 °C. **c**, Self-extinguishing time of electrolytes with different concentrations of organic solvents, measured by igniting glass fibre separators soaked with 200 µl of electrolyte solution. The error bar represents the standard deviation based on three independent measurements. Source data

The ESWs of electrolyte solutions with various ether-based additives were investigated by using differential electrochemical mass spectrometry. The 1.8-mol%-DEE-containing electrolyte solution demonstrates an improved cathodic stability, compared with the other ether-containing electrolytes, with an HER potential shift from –1.05 V to –1.27 V (versus Ag|AgCl), compared with AE, attributed to the water-repelling ability of DEE-LEI (Fig. 4a and Supplementary Fig. 30), aligning well with the in situ ATR-SEIRAS result. On the anodic side, the OER potential also extends from 1.70 V to 1.81 V (versus Ag|AgCl), compared with the AE, yielding a wider ESW of 3.08 V, compared with 2.96 and 2.92 V for the 1.8-mol%-DME-containing and the 1.8-mol%-ME-containing electrolyte solutions, respectively.

Importantly, the expanded ESW in the 1.8-mol%-DEE-containing electrolyte solution does not compromise the advantages of AEs, such as fast ion conduction and safety. The 1.8-mol%-DEE-containing electrolyte exhibits a bulk ionic conductivity of 54.46 mS cm^−1^ at 25 °C and remaining non-flammable (Fig. 4b,c and Supplementary Fig. 31). In comparison, electrolyte solutions with the ME and DME additives, with weak-to-moderate adsorption strength, require higher additive concentrations to achieve a wide ESW, which could diminish the ionic conductivity substantially and introduce the flammability risk. ME- and DME- containing electrolyte solutions become flammable at additive concentrations of 16.2 mol% and 12.6 mol%, respectively (Supplementary Fig. 31), and their bulk ionic conductivities drop to 23.86 mS cm^−1^ and 23.99 mS cm^−1^ at 25 °C, respectively (Fig. 4b).

### Electrochemical behaviour of Zn electrodes with ether-containing AE solutions

**Fig. 5:**
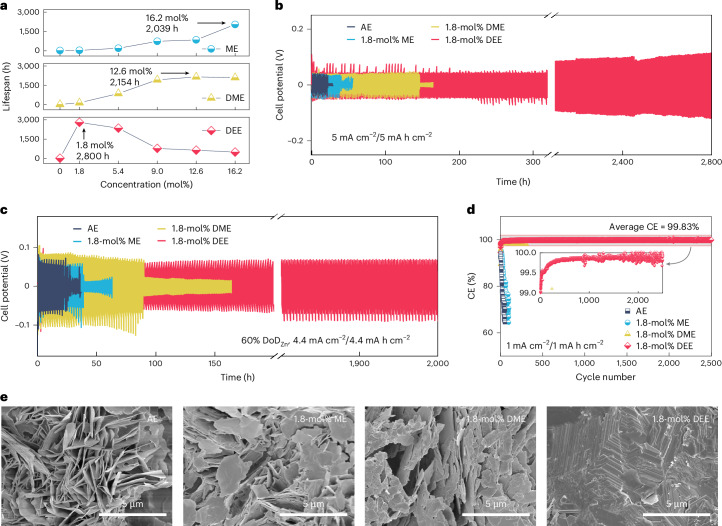
Electrochemical behaviour of Zn electrodes with ether-containing AE solutions in symmetric and asymemtric coin cells. **a**, Correlation between the lifespan of Zn||Zn cells and concentrations of ME, DME and DEE in 3-m Zn(OTf)_2_ electrolyte solutions. **b**, Potential profiles of Zn||Zn coin cells during stripping/plating at 5 mA cm^−2^, 5 mA h cm^−2^ and 25 °C. **c**, Potential profile of Zn||Zn cells with a 60% DoD_Zn_ at 4.4 mA cm^−2^, 4.4 mA h cm^−2^ and 25 °C. **d**, CE of Zn||Cu cells cycled at 1 mA cm^−2^, 1 mA h cm^−2^ and 25 °C. **e**, Ex situ SEM micrographs of Zn electrodes after plating at 5 mA cm^−2^ and 25 °C with a capacity of 20 mA h cm^−2^. Source data

Figure 5a illustrates the correlation between the lifespan of Zn||Zn symmetric coin cells and the concentration of ME, DME and DEE additives in the 3-m Zn(OTf)_2_ AE solution. The cell potential profiles during multiple stripping and plating are presented in Supplementary Fig. 32. With the formed DEE-LEI, the optimal DEE additive concentration in the electrolyte solution is 1.8 mol%, whereas for the ME- and DME-containing electrolytes, the concentrations are 16.2 mol% and 12.6 mol%, respectively. The 1.8-mol%-DEE-containing electrolyte enables a prolonged cell lifespan of 2,800 h at 5 mA cm^−2^ and 5 mA h cm^−2^, which is over 20 times longer than those cells using electrolytes with the ME (38 h) and DME (146 h) at the same concentration (Fig. 5b). The performance decline at high DEE concentrations stems from excessive micelle formation in the bulk electrolyte, which disrupts the uniformity of Zn^2+^ flux^34,35^. The 1.8-mol%-DEE-containing electrolyte also enables extended lifespans of 2,000 h and 600 h for the Zn||Zn coin cells under high DoD_Zn_ of 60% and 80%, respectively (Fig. 5c and Supplementary Fig. 33). The 1.8-mol%-DEE-containing electrolyte further exhibits a lower corrosion current of 0.073 mA cm^−2^ and a lower corrosion rate of 2.77 µg h^−1^, which are less than half of those in the baseline AE (Supplementary Figs. 34 and 35). Consistently, fewer Zn_*x*_OTf_*y*_(OH)_2*x*−*y*_ H_2_O corrosion by-products are observed on the Zn surface after 7 days of static storage (Zn foil stored in electrolytes) and after 40 h of cycling in cells (Supplementary Figs. 36 and 37), underscoring the anticorrosion ability of the 1.8-mol%-DEE-containing electrolyte solution. With a mitigated HER, the Zn||Cu asymmetric cell cycled in 1.8-mol%-DEE-containing electrolyte achieves an average CE of 99.83% after 2,500 cycles at 1 mA cm^−2^, 1 mA h cm^−2^ and 25 °C. (Fig. 5d), outperforming cells with ME and DME electrolytes (CE < 99%). A high average CE of 99% was achieved in the 1.8-mol%-DEE-containing electrolyte (Supplementary Fig. 38), when testing Zn||Cu asymmetric coin cells with Aurbach’s method^36,37^. In addition, the Zn electrode in 1.8-mol% DEE shows electrode kinetics comparable with AE, with low overpotential even at 30 mA cm^−2^ and 25 °C (14.6 mV for DEE electrolyte versus 17.4 mV for AE; Supplementary Fig. 39).

The adsorption of the DEE-LEI also regulates the growth of Zn during plating/stripping, resulting in improving the morphology of the electrodeposited zinc on the Cu electrodes (Supplementary Fig. 40). The preferential adsorption of DEE-LEI on the Zn(002) facet promotes orientated Zn deposition along the Zn(002) facet, which is evidenced by close-packed and ladder-pattern morphology (Fig. 5e) as well as a higher *I*_(002)_/*I*_(101)_ ratio of 0.895 compared with those for ME (0.289) and DME (0.294) electrolytes (Supplementary Fig. 41). This ratio continues to increase over 400 h of cycling, indicating that the (002) facet becomes increasingly dominant (Supplementary Fig. 42). The Zn deposition in cells using the 1.8-mol%-DEE-containing electrolyte is dense and uniform with planar three-dimension morphology on the Zn(002) facet, as confirmed by confocal laser microscopy images (Supplementary Fig. 43) and the stable deposition current (Supplementary Fig. 44). As such, Zn||Zn symmetric cells with 1.8-mol%-DEE-containing electrolyte demonstrate a longer cycle life even under large current densities (Supplementary Fig. 45). Post-mortem microscopy analysis shows a homogeneous and compact morphology of Zn deposition after prolonged cycles (Supplementary Figs. 46 and 47). Additionally, neither the solvation structure nor the pH appears to account for the improved Zn reversibility (Supplementary Figs. 48–55 and Supplementary Note 2).

Compared with ME, DME and other co-solvents/additives used in aqueous-based zinc electrolyte solutions, the use of the DEE additive enables improved performance in lifespan and accumulated capacity (Supplementary Table 3), with the optimal dosage of 1.8-mol% DEE being equal to ~11 wt% of the electrolyte solvents and ~5 wt% of the whole electrolyte solution. A comparison with conventional surfactant additives also reveals that the use of 1.8-mol%-DEE-containing electrolyte enables improved performance in terms of CE, electrode polarization and cell cycle life (Supplementary Figs. 56 and 57, Supplementary Table 4 and Supplementary Note 3). As a proof of concept, we also extended our electrolyte design, adapting it to various salts, such as Zn(ClO_4_)_2_ and ZnSO_4_, and organic solvents/additives, such as carbonates and alcohols, demonstrating the broad applicability of the LEI strategy (Supplementary Figs. 58–68 and Supplementary Note 4).

### Electrochemical characterizations of Zn||NaV_3_O_8_ cells with ether-containing AE solutions

**Fig. 6:**
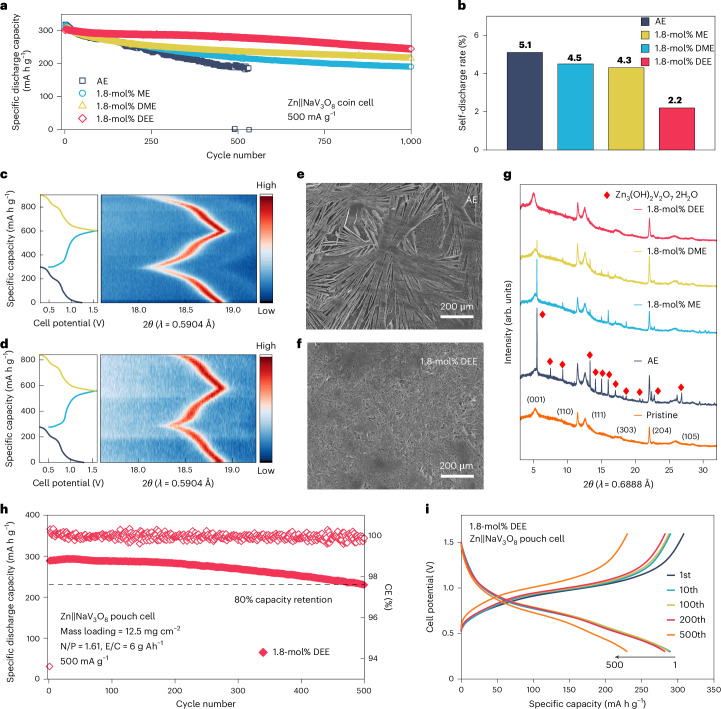
Electrochemical and physicochemical characterizations of Zn||NaV_3_O_8_ cells with ether-containing AE solutions. **a**,**b**, Cycling performance (**a**) and daily self-discharge rate (**b**) of Zn||NaV_3_O_8_ coin cells at 500 mA g^−1^ and 25 °C. The specific discharge capacity is calculated based on the mass of the NaV_3_O_8_ active material. The self-discharge rate was calculated from the capacity decay of individual cells, with the corresponding potential profiles shown in Supplementary Fig. 73. **c**,**d**, Contour plot of NaV_3_O_8_(204) reflection evolution obtained via operando synchrotron-based XRPD measurements carried out during Zn||NaV_3_O_8_ coin cell cycling using the baseline AE (**c**) and 1.8-mol%-DEE-containing (**d**) electrolyte solutions. The black, light blue and dark yellow potential profile curves correspond to the initial discharge, initial charge and second discharge, respectively. The colour bar represents the intensity of the XRPD patterns, with red indicating high intensity and blue indicating low intensity. **e**,**f**, Ex situ SEM micrographs of aged NaV_3_O_8_ electrode after 200 cycles in Zn metal coin cells using the baseline AE (**e**) and 1.8-mol%-DEE-containing (**f**) electrolyte solutions. **g**, Ex situ synchrotron-based XRPD patterns of pristine and aged NaV_3_O_8_ electrode after 200 cycles in Zn||NaV_3_O_8_ coin cell configuration at 500 mA g^−1^ and 25 °C. **h**,**i**, Cycling performance (**h**) and selected potential profiles (**i**) of a Zn||NaV_3_O_8_ single-layer pouch cell with the 1.8-mol%-DEE-containing electrolyte and an N/P ratio of 1.61, E/C ratio of 6 g A h^−1^ cycled at 500 mA g^−1^ and 25 °C. The specific discharge capacity is calculated based on the mass of the NaV_3_O_8_ active material. Source data

The DEE-LEI was also formed on the positive electrode side, as indicated by the reduced contact angle between the 1.8-mol%-DEE-containing electrolyte and the NaV_3_O_8_ electrode (Supplementary Fig. 69). QCM analysis further show an adsorbed DEE mass of ~0.0164 mg cm^−2^ on the NaV_3_O_8_ positive electrode, which is slightly higher than that on the Zn negative electrode, consistent with its larger specific surface area (Supplementary Fig. 70). Figure 6a shows the specific discharge cycle performance of the Zn||NaV_3_O_8_ coin cells in four different electrolyte solutions with various ether additive types. The Zn||NaV_3_O_8_ coin cell exhibiting a higher specific discharge capacity retention is the one with the 1.8-mol%-DEE-containing electrolyte (82% after 1,000 cycles at 500 mA g^−1^). The Zn||NaV_3_O_8_ coin cells with the 1.8-mol%-ME-containing and 1.8-mol%-DME-containing electrolyte solutions retain 61% and 71% of the initial specific discharge capacity, respectively, at 500 mA g^−1^ after 1,000 cycles. Improved cycle stability is also demonstrated at varied specific currents ranging from 125 mA g^−1^ to 2,000 mA g^−1^ (Supplementary Fig. 71), with no decay in rate performance compared with that of baseline AE (Supplementary Fig. 72). The Zn|1.8-mol%-DEE-containing electrolyte|NaV_3_O_8_ coin cell also exhibits a lower self-discharge rate (2.2% per day) (Fig. 6b and Supplementary Fig. 73) than those for the same type of cells using the baseline AE, 1.8-mol% ME and 1.8-mol% DME electrolytes.

Operando synchrotron-based X-ray powder diffraction (XRPD) reveals the underlying mechanism of the battery performance improvement for the Zn||NaV_3_O_8_ cells. The formation of BZS (Zn_*x*_OTf_*y*_(OH)_2*x*−*y*_ H_2_O) phases during discharge, indicative of Zn^2+^/H^+^ co-intercalation, is fully reversible on charging in all electrolytes and, thus, does not contribute to capacity fading (Supplementary Fig. 74 and Supplementary Note 5). Importantly, for cells with the baseline AE electrolyte, we observed that Zn^2+^/H^+^ insertion expands the interlayer spacing of the (204) plane in the NaV_3_O_8_ electrode on initial discharge to 0.3 V, leading to a downshift in the (204) plane to a lower 2*θ* value from 18.88° to 18.19°, and further to 18.07° in the second discharge to 0.3 V (Fig. 6c). By contrast, minor peak shifts were observed for the NaV_3_O_8_ electrode tested in the Zn metal configuration with the 1.8-mol%-DEE-containing electrolyte (from 18.88° to 18.44°), lower than those in 1.8-mol%-DME-containing (from 18.88° to 18.29°) and 1.8-mol%-ME-containing (from 18.88° to 18.23°) electrolyte solutions, indicating reduced structural strain with mitigated vanadium dissolution when the 1.8-mol%-DEE-containing electrolyte solution is used (Fig. 6d and Supplementary Figs. 74 and 75). This can be attributed to the presence of DEE-LEI on the positive electrode surface, which prevents excessive water penetration, resulting in reduced size of the solvated Zn^2+^ and H^+^ ions during their intercalation into the NaV_3_O_8_ electrode^38^. As such, an alleviated lattice strain of NaV_3_O_8_ was observed during the discharge of cells using the 1.8-mol%-DEE-containing electrolyte. It should be pointed out that this effect does not negate the beneficial role of a small, structurally accommodating amount of interlayer water in facilitating H^+^ and Zn^2+^ diffusion^39^. Rather, it underscores the importance of suppressing excessive H_2_O co-insertion to avoid the positive electrode’s structural disruption and to achieve optimized Zn||NaV_3_O_8_ cells cycling stability and rate capability^40^.

The formation of Zn_3_(OH)_2_V_2_O_7_ 2H_2_O (ZVO) by-products is another source of capacity fading in Zn||NaV_3_O_8_, affecting the positive electrode. During cycling, OH^−^ anions produced by HER at the Zn electrode shuttle to the NaV_3_O_8_-based electrode surface, leading to the formation of high-impedance ZVO by-products on the positive electrode surface^41,42^. Abundant strip-like by-products were observed on the surface of the NaV_3_O_8_-based electrode after 200 cycles in zinc metal cells using the baseline AE at 500 mA g^−1^ and 25 °C (Fig. 6e,g), resulting in sluggish charge transfer kinetics of the Zn^2+^ and H^+^ insertion/de-insertion. By contrast, the formed DEE-LEI is effective to mitigate the HER and diffusion of OH^−^, with a lower amount of by-product observed on the positive electrode surface (Fig. 6f,g). Furthermore, other hydrated Zn^2+^ and H^+^ co-intercalation positive electrode active materials, such as V_2_O_5_ and MnO_2_, exhibit improved cycling stability when tested in zinc metal coin cells with the 1.8-mol%-DEE-containing electrolyte compared with AE, ME and DME, confirming the broad applicability of the DEE-LEI strategy (Supplementary Fig. 76).

Because of the stabilization of DEE-LEI, Zn||NaV_3_O_8_ coin cells in the 1.8-mol%-DEE-containing electrolyte show a specific discharge capacity retention of 76% after 1,500 cycles at 2,000 mA g^−1^ even at 50 °C (Supplementary Fig. 77). Also, the 1.8-mol%-DEE-containing electrolyte exhibits adequate bulk ionic conductivity at below-zero temperatures, for example, 5.58 mS cm^−1^ at −35 °C (Supplementary Fig. 78). This enables Zn||NaV_3_O_8_ cells to operate at −35 °C, delivering stable cycling performance and specific discharge capacities at 2,000 mA g^−1^ (which represent 56% retention of the specific discharge capacity at 25 °C; Supplementary Fig. 79). We further assembled a Zn||NaV_3_O_8_ single-layer pouch cell with N/P ratio of 1.61 and E/C ratio of 6 g Ah^−1^, which demonstrates a lifespan of 500 cycles at 500 mA g^−1^ and 25 °C with an average CE of 99.95% and a specific discharge capacity retention of 80% (Fig. 6h,i), which is well-aligned performance compared with selected state-of-the-art reports (Supplementary Table 5). The calculated initial specific energy of the Zn||NaV_3_O_8_ single-layer pouch cell is 132 Wh kg^−1^ based on the combined mass of the Zn negative electrode and composite NaV_3_O_8_-based positive electrode.

Furthermore, the LEI-forming electrolyte enables the stable operation of high-voltage Li^+^-intercalation cathodes beyond the ESW of conventional AE (up to 2.5 V), with solvent selection playing a critical role in balancing interfacial stability and oxidative tolerance. Benchmarking against representative high-concentration electrolytes and organic/aqueous hybrid electrolytes further demonstrates the ability of our electrolyte solution design to improve ionic conductivity, rate capability and specific energy (Supplementary Figs. 80–93, Supplementary Table 6 and Supplementary Notes 6 and 7).

## Conclusion

In this research work, we introduce the concept of LEIs for AZBs formed on both positive and negative electrodes. We demonstrated that the ideal electrode-philic additive for the LEI formation should possess both high adsorption ability (contact angle, <65°) and strong hydrophobicity (log[*P*_oct/wat_] > 0) to facilitate the aggregation of hydrophobic organic molecules on the Zn surface. Additionally, it should exhibit weak solvating ability (donor number, <18) to ensure a compact structure for a fully covered LEI. The water-repulsive ability of the LEI suppresses water splitting on the Zn negative electrode and H_2_O co-insertion into the NaV_3_O_8_-based positive electrode, resulting in highly reversible Zn plating/stripping, and long cycle life for Zn||NaV_3_O_8_ cells. When LEI-forming electrolyte solution is tested in a Zn||NaV_3_O_8_ single-layer pouch cell configuration (with N/P and E/C ratios of 1.61 and 6 g Ah^−1^, respectively), an extended lifespan of 500 cycles at 500 mA g^−1^ is attainable with a calculated initial specific energy of 132 Wh kg^−1^. The electrolyte also exhibits wide temperature adaptability, supporting the stable operation of Zn||NaV_3_O_8_ cells over a temperature range of −35 to +50 °C. Moreover, the LEI-forming electrolytes extend the accessible ESW to stabilize Li-based high-potential positive electrodes (up to 2.5 V). Future improvements could be realized by rational molecular design to enhance interfacial adsorption, hydrophobicity and oxidative stability, for example, by extending alkyl chains or partially fluorinating the molecules, as well as maintaining weak Zn^2+^ solvation. These strategies are expected to further improve Zn plating/stripping CE beyond 99.9% and enable an operating cell potential of over 2.5 V, helping unlock the potential of AZBs for large-scale energy storage.

## Methods

### Electrolyte solution preparation

All chemicals were used as received without purification. Salts and organic solvents were purchased from Sigma-Aldrich. Salts used in this study include Zn(OTf)_2_ (purity, 98%), ZnSO_4_·7H_2_O (purity, 99.5%), Zn(ClO4)_2_·6H_2_O (purity not specified) and lithium trifluoromethanesulfonate (purity, 99.995%). Organic solvents include ME (CH_3_OCH_2_CH_2_OH; purity, 99.0%), DME (CH_3_OCH_2_CH_2_OCH_3_; purity, 99.0%), DEE (CH_3_CH_2_OCH_2_CH_2_OCH_2_CH_3_; purity, 98.0%), diglyme ((CH_3_OCH_2_CH_2_)_2_O; purity, 99.0%), 1,4-dioxane (C_4_H_8_O_2_; purity, 99.0%), 1,3-dioxolane (C_3_H_6_O_2_; purity, 99.0%), methanol (CH_3_OH; purity, ≥99.8%), ethanol (CH_3_CH_2_OH; purity, ≥99.5%), 1-butanol (CH_3_(CH_2_)_3_OH; purity, 99.8%), dimethyl carbonate ((CH_3_O)_2_CO; purity, ≥99.9%), diethyl carbonate ((C_2_H_5_O)_2_CO; purity, ≥99.0%), ethylene carbonate (C_3_H_4_O_3_; purity, ≥99.0%), propylene carbonate (C_4_H_6_O_3_; purity, ≥99.0%), γ-butyrolactone (C_4_H_6_O_2_; purity, ≥99.0%), *N*,*N*-dimethylformamide (HCON(CH_3_)_2_; purity, ≥99.8%), acetonitrile (CH_3_CN; purity, ≥99.9%), trimethyl phosphate ((CH_3_O)_3_PO; purity, ≥99.0%,), dimethyl sulfoxide ((CH_3_)_2_SO; purity, ≥99.9%) and sulfolane (C_4_H_8_O_2_S; purity, ≥99.0%). The zinc salt concentration was controlled at 3 mol kg^−1^ (3 m) where the mass of the solvent is the mass of water (ultrapure water, approximately 18.2 MΩ cm at 25 °C, purified by a Milli-Q water purification system) and the organic additives in various molar percentage values. Additional 2 m of lithium trifluoromethanesulfonate was added into 3-m Zn(OTf)_2_ electrolyte solutions with the ether additives to provide Li^+^ ions for intercalation/deintercalation at the LiMn_2_O_4_ and LiVPO_4_F positive electrode.

### Electrode preparation

To synthesize the NaV_3_O_8_ positive electrode active material, 3 g of commercial V_2_O_5_ powder (Sigma-Aldrich; purity, ≥98.0%) was added into 100 ml of 2 mol l^−1^ of NaCl aqueous solution (Sigma-Aldrich; purity, ≥99.0%) and stirred magnetically at 400 rpm for 72 h in air under a fume hood in a glass beaker. The resulting orange–red powders were washed with 300 ml of deionized water for three cycles. In each washing step, the powders were dispersed in water and stirred at 400 rpm for 5 min in a glass beaker, followed by collection via centrifugation at 8,000 rpm for 10 min. The obtained powders were subsequently freeze dried at temperatures below −40 °C under a vacuum of <50 Pa for 20 h. The MnO_2_ (purity, ≥99.0%; water content, ≤50 ppm) and LiMn_2_O_4_ (purity, ≥99.0%; water content, ≤800 ppm) powders were purchased from Carnd Technology, whereas LiVPO_4_F (purity, ≥99.0%; water content, ≤500 ppm) powders were supplied from Advanced Lithium Electrochemistry. All powders were dried at 120 °C under vacuum for 12 h before use. Composite positive electrodes were prepared by mixing the active material powder, electron-conductive carbon additive (Super P, Carnd Technology; purity, ≥99.5%, primary particle size, ≤50 nm; specific surface area, ≥62 m^2^ g^−1^) and polytetrafluoroethylene (Carnd Technology, 60 wt% dispersion in H_2_O) in a mass ratio of 7:2:1 in ethanol, followed by manually grinding using an agate mortar and pestle for 10 min in air at 25 °C. The dough-like electrode slurry was placed onto a titanium (Ti) mesh current collector (Carnd Technology; purity, ≥99.5%; thickness of 0.27 mm, 100 mesh, pore size of 0.15 mm) and roll pressed using a roller press (MSK-2150-H5, MTI). The electrode was pressed repeatedly (typically 5–10 passes) with a controlled roller gap to ensure uniform thickness and good adhesion between the active material and the current collector. The mass loading of the active material was controlled to approximately 12.5 mg cm^−2^ by adjusting the electrode area after roll pressing. The obtained electrodes were then dried at 80 °C overnight to remove any residual H_2_O and ethanol. The dry electrodes were cut using a precision disc cutter (MSK-T-10, MTI) before coin cell assembly. The positive electrodes obtained were stored in a vacuum desiccator before cell assembly and tested in a coin cell and pouch cell configuration. Zn foils (purity, ≥99.995%; thickness of 10, 15 or 100 µm) and Cu foil (purity, ≥99.95%; thickness of 9 µm) were purchased from Carnd Technology. The Zn negative electrodes and Cu current collectors were cut into disc-shaped electrodes using a precision disc cutter (MSK-T-10, MTI) before coin cell assembly. Before cutting, the Zn foil was polished with softback sanding sponges (3M) and then wiped with ethanol, whereas the Cu foil was cleaned by wiping with ethanol. For pouch cell assembly, the NaV_3_O_8_ positive electrodes, Zn negative electrodes and Cu current collectors were cut using a home-made cutter.

### Electrochemical measurements

CR2025 coin-type cells were assembled in air at 25 °C using stainless steel (SS) cases and springs, with glass fibre membranes (Filtech; thickness of 0.66 mm and diameter of 19 mm) as the separator and an electrolyte solution volume of 100 µl. The electrolyte was transferred using a calibrated single-channel air-displacement micropipette (20–200 µl; Thermo Fisher Scientific) equipped with disposable 200-µl polypropylene tips, and applied dropwise onto the separator to ensure uniform wetting. The electrodes used in the coin cells were 12 mm in diameter. The crimping load applied for the coin cell assembly was of 0.5 t. For the Zn||NaV_3_O_8_ single-layer pouch cells, the composite positive electrode dimensions were 3 × 4 cm^2^, with a hydrophilic sulfonated composite separator (Carnd Technology; thickness of 0.152 mm, lateral size of 3.5 × 4.5 cm^2^, porosity of ≥88% and pore size of ≤20 μm) and a 10-µm-thick zinc foil (lateral size of 3 × 4 cm^2^). Nickel tabs (Carnd Technology; purity, ≥99.5%) were attached to both positive current collector (Ti mesh) and negative current collector (Cu) by ultrasonic welding (MSK-800-2K, MTI). The single-layer electrode stack was then placed into an aluminium-laminate pouch. The electrolyte was injected into the pouch using a pipette (100–1,000 µl, Thermo Fisher Scientific), with the electrolyte amount controlled at an E/C ratio of 6 g Ah^−1^. The pouch cell was subsequently vacuum sealed at ~10^−2^ torr for 60 s and heat sealed at 180 °C for 6 s (MSK-115A-S, MTI). After sealing, the cells were rested for 12 h to ensure complete electrolyte wetting before testing. An external pressure of approximately 0.1 MPa was applied to the pouch cell by sandwiching it between two rigid acrylic plates during testing. Charge–discharge tests of batteries were performed on a Neware battery test system (CT-3008) in an air-conditioned laboratory environment with an average temperature of 25 ± 1 °C. The charge–discharge tests of Zn||NaV_3_O_8_ coin cells at −35 °C was conducted in a temperature-controlled climatic chamber (GWS MT3065), with an average temperature deviation of ±0.5 °C. The mass used to calculate the specific current and specific capacity refers to the mass of the positive electrode active material. Three cells were tested for each electrochemical experiment, and consistent performance was observed across all cells. The results presented in the figures correspond to a representative cell; additional data from the other cells are available on request.

The bulk ionic conductivities (*σ*) and pH values of electrolytes at 25 °C were measured using a pH/conductivity multiparameter benchtop meter (Thermo Orion Versa Star Pro). Electrochemical impedance spectroscopy (EIS) measurements were used to measure bulk ionic conductivities at −35 °C, with the cell constant *K*_cell_ determined based on the bulk ionic conductivity at 25 °C. EIS measurements were carried out using SS||SS (Carnd Technology, 304; thickness of 15 μm, diameter of 16 mm, used as received) CR2025 coin cells (assembled as described above) on a VMP-300 potentiostat (BioLogic) using the potentiostatic mode with an amplitude of 5 mV and frequencies ranging from 500 kHz to 10 Hz, collecting 5 points per decade. Before each EIS measurement, the system was stabilized at the open-circuit potential for 300 s to ensure a steady state. The bulk ionic conductivity of an electrolyte solution at −35 °C was calculated using the following equation:

1 $$\sigma =\frac{L}{{SR}}=\frac{{K}_{\mathrm{cell}}}{R},$$

where *L* is the distance between the two SS electrodes, *S* is the contact area of the SS electrodes and *R* is the resistance value (in Ω) extrapolated at the intersection between the raw EIS data and the real impedance axis.

The corrosion rate (Corr) was evaluated by monitoring the potential of a Zn@Ti electrode, which was prepared by depositing 0.565 mAh of metallic Zn onto a Ti foil (Carnd Technology; purity, ≥99.89%, thickness of 20 μm and diameter of 12 mm). The measurement was conducted in Zn||Ti coin cells (assembled as described above). The Corr is determined using the following equation:

2 $$\mathrm{Corr}=\frac{{m}_{\mathrm{Zn}}}{t},$$

where ${m}_{{\rm{Zn}}}$ represents the total mass of the deposited Zn metal and $t$ is the corrosion time corresponding to the complete consumption of Zn.

The HER and OER potentials of the electrolyte solutions were investigated using an HPR-40 differential electrochemical mass spectrometer (HIDEN Analytical), coupled with a gold (Au) working electrode, an Ag|AgCl reference electrode and a Pt counter electrode at a scan rate of 5 mV s^−1^ at 25 °C.

Cyclic voltammetry (CV) tests were conducted on symmetric Zn||Zn coin cells (assembled as described above) over a potential range of −15 mV s^−1^ to 15 mV s^−1^ at specified scan rates and 25 °C.

The reduction stability of water and ether additives were investigated by linear sweep voltammetry tests using a Ti working electrode, an Ag|AgCl reference electrode and a Pt counter electrode at a scan rate of 5 mV s^−1^ at 25 °C.

Tafel tests were conducted on symmetric Zn||Zn coin cells over a potential range of −20 mV s^−1^ to 20 mV s^−1^ at a scanning rate of 10 mV s^−1^ and 25 °C.

### Ex situ physicochemical characterizations

The contact angle of the electrolyte solutions on the zinc metal negative electrode and the NaV_3_O_8_ positive electrode was measured using an optical tensiometer (Attension Theta, Biolin Scientific). Electrolyte droplets (approximately 3 µl) were automatically dispensed onto the electrode surface using a microsyringe. The contact angle was determined from the droplet profile and recorded after 10 s as a stable value. Each measurement was performed three times at different locations on the electrode surface, and the average value was reported.

FTIR spectroscopic measurements were performed using a Nicolet 6700 Thermo Fisher FTIR spectrometer in the attenuated total reflection mode.

Raman spectra were collected using a LabRAM HR Evolution Raman microscope (Horiba Jobin Yvon) with a 532-nm laser.

SAXS measurements were carried out in the capillary transmission mode at the SAXS/wide-angle X-ray scattering beamline of the ANSTO—Australian Synchrotron.

NAP-XPS was conducted at the TLS 24A1 beamline of the National Synchrotron Radiation Research Center. The chamber vacuum was controlled at 1 mbar.

QCM with dissipation monitoring measurements were performed using 5.0-MHz AT-cut quartz crystals precoated with gold (14.0-mm diameter; Biolin, QX301). Zn powders (40–60 nm and purity of 99%; Sigma-Aldrich) or NaV_3_O_8_ powders were mixed with poly(vinylidene fluoride) (Sigma-Aldrich) at a weight ratio of 9:1 in 1-methyl-2-pyrrolidinone (Sigma-Aldrich; purity, ≥99.5%) to form a homogeneous slurry. The slurry was spin-coated onto the quartz crystals at 8,000 rpm and subsequently dried in a vacuum oven at 40 °C for 12 h. The QCM measurements were carried out in a flow-cell configuration. Initially, a baseline was established by flowing ultrapure H_2_O at a rate of 100 μl min^−1^ until a stable frequency signal was obtained, where water served as the reference adsorbed species. Subsequently, the electrolyte was switched to the 1.8-mol%-DEE-containing solution, and the resulting frequency changes were recorded. The adsorbed mass of DEE was calculated from the frequency shift using the Sauerbrey equation. The fundamental resonance frequency and its overtones (third, fifth and seventh) were recorded simultaneously, together with the corresponding dissipation factors. Data acquisition and analysis were performed using QSoft401 (v.2.8.4.948) and QSense Dfind (v.1.2.8) software^43^.

The cycled electrodes for FTIR, X-ray diffraction and scanning electron microscopy (SEM) characterization were harvested by disassembling the cells in ambient air. The electrodes were rinsed three times with ultrapure water to remove residual electrolytes and then dried in ambient conditions for 24 h before analysis.

For the XPS measurements, the cells were disassembled in an Ar-filled glovebox (Ar gas; BOC Australia; 99.999%; H_2_O and O_2_ content, <0.1 ppm). The electrodes were transferred into glass vials, washed three times with 3 ml of dimethyl carbonate (Sigma-Aldrich; purity, ≥99.9%; water content, ≤10 ppm), and then vacuum dried. To prevent air exposure, the dried samples were transferred directly to the XPS instrument using an airtight container purged with Ar gas.

X-ray diffraction measurements of Zn electrodes were conducted using Rigaku Ultima IV with monochromatic Cu Kα radiation, scanning between 5° and 80° at a rate of 10° min^−1^. Ex situ XRPD measurements of NaV_3_O_8_ electrodes was performed at the Powder Diffraction beamline of the ANSTO—Australian Synchrotron, using an X-ray beam with a wavelength of 0.68880 Å. Diffraction patterns were collected using a MYTHEN microstrip detector with an exposure time of 30 s.

Morphological images and surface roughness of electrodes were obtained using SEM (Hitachi SU7000) and a confocal microscope (Olympus LEXT OLS5000 profilometer).

XPS measurements of Zn electrodes were performed on Thermo Scientific Nexsa using monochromic Al Kα radiation.

Flammability tests of electrolyte solutions were conducted by igniting a glass fibre separator soaked with 200 µl of electrolyte using a gas lighter for 3 s. The self-extinguishing time, defined as the time required for flame extinction, was recorded, with each test repeated three times.

Titration tests were performed to evaluate the pH-buffering behaviour of the electrolyte solutions. In each measurement, 3 ml of electrolyte solution was placed in a glass vial and stirred at 100 rpm at 25 °C. A 0.1 mol l^−1^ of NaOH aqueous solution was then added stepwise in increments of 30 µl using a pipette (10–100 µl; Thermo Fisher Scientific), and the pH value was recorded after each addition using a calibrated pH meter.

The amounts of dissolved vanadium in separators were determined by inductively coupled plasma mass spectrometry (Agilent 8900x QQQ-ICP-MS).

### In situ and operando physicochemical characterizations of zinc cells

The in situ observation of the adsorption layer on the Zn surface was conducted using the ATR-SEIRAS technique. Specifically, a thin Zn film with a thickness of 50 nm was deposited on an Au@Si wafer (Shanghai Yuanfang; thickness of 500 μm and lateral dimensions of 1.1 × 0.9 cm^2^). The obtained Zn@Au@Si wafer was used as the working electrode, with an Ag|AgCl reference electrode and a Pt counter electrode, all assembled in a custom-designed spectro-electrochemical cell. The ATR-SEIRAS measurements were conducted using a Nicolet iS50 FTIR spectrometer, equipped with a narrowband MCT-A detector and an in situ infrared optical accessory (SPEC-I, Shanghai Yuanfang) at an incidence angle of 45°. For static adsorption in the absence of an external electric field (Fig. 3c and Supplementary Figs. 19 and 21), the background spectrum was collected before the injection of the electrolyte solution. Continuous spectral acquisition was then conducted until an equilibrium state was observed. For the Zn plating observation (Fig. 3d and Supplementary Figs. 27 and 28), the background was set as the equilibrium adsorption state. The plating current density was set to be 0.5 mA cm^−2^.

Operando synchrotron-based XRPD was performed at the Powder Diffraction beamline of the ANSTO—Australian Synchrotron. Zn||NaV_3_O_8_ CR2025 coin cells with 4-mm-diameter windows were used to ensure synchrotron beam transmission. The customized Zn||NaV_3_O_8_ coin cells (assembled as described above) were tested at 500 mA g^−1^ between 0.3 V and 1.6 V and 25 °C. Diffraction patterns were collected using a MYTHEN microstrip detector with an exposure time of 30 s, and data were recorded at 3-min intervals.

The wavelength of the synchrotron X-ray beam for operando XRPD experiments was 0.59040 Å, whereas ex situ XRPD experiments used a wavelength of 0.68880 Å. This variation does not affect the reliability of the results, as both experiments provided sufficiently strong X-ray intensity to detect the species present on NaV_3_O_8_ electrodes with sufficient sensitivity.

### Statistical analysis

The statistical analysis was performed using in-house developed code written in Python language (Python v.3.10.13). Specifically, the Pearson correlation coefficient was calculated using the ‘corrcoef’ function from the Numpy library (v.1.26.2)^44^. Mutual information was calculated with the ‘mutual_info_regression’ function from the Scikit-learn library (v.1.3.0)^45^. Feature importance was calculated using the ‘permutation_importance’ function in conjunction with the ‘RandomForestRegressor’ machine learning algorithm, both implemented in the Scikit-learn library (v.1.3.0) with default parameters^45^. The equation between CE and four descriptors was obtained by fitting a Ridge Regression, as implemented in the Scikit-learn library as well.

### Computational methods

All MD simulations were performed using the GAFF force field^46^. The ACPYPE was used to obtain the GAFF force field topology^47^. The simulation box size was 5 × 5 × 5 nm^3^ for all simulation models, which consisted of Zn^2+^, OTf^−^ and H_2_O, without and with ME/DME/DEE molecules. The cut-off distance of 1.2 nm was used for the Lennard–Jones potential. The Coulombic potential was measured using particle mesh Ewald with a cut-off distance of 1.2 nm and Fourier grid spacing of 0.12. All bonds were constrained with the LINCS algorithm and periodic boundary conditions were applied in all directions. The MD simulations were started by running initial energy minimization, followed by 1,500 ps of *NVT* simulation and 1,500 ps of *NPT* simulation, with an integration time step of 0.001 ps. All simulation systems were finally maintained at 298 K using the Nosé–Hoover thermostat for 30 ns to collect the simulation data. A time constant of 1 ps was applied for the temperature coupling. The calculations of proton diffusion coefficients, hydrogen bonds and partial density were conducted using GROMACS.

The adsorption energy of water, ME, DME and DEE molecules on the Zn surfaces of (101) and (002) facets was investigated using density functional theory. The density functional theory calculations were implemented using the Vienna ab initio simulation package^48,49^ with the core and valence electronic interactions being modelled using the projector augmented-wave method^50,51^. The revised Perdew–Burke–Ernzerhof exchange–correlation functional was used^52^. The wavefunction was expanded with a kinetic energy cut-off of 500 eV and a Γ *k*-point were used. The dispersion correction was also considered in this study by using DFT-D3 method^53^. The adsorption energy (*E*_ads_) was calculated using the following equation:

3 $${E}_{\mathrm{ads}}={E}_{\mathrm{Zn}-\mathrm{surface}+\mathrm{adsorbents}}-{E}_{\mathrm{Zn}-\mathrm{surface}}-{E}_{\mathrm{adsorbents}},$$

where *E*_Zn-surface + adsorbents_, *E*_Zn-surface_ and *E*_adsorbents_ are the total electronic energies for the Zn surface with adsorbed species, clean Zn surface and adsorbed species (including water, ME, DME and DEE molecules), respectively.

### Reporting summary

Further information on research design is available in the Nature Portfolio Reporting Summary linked to this article.

## Online content

Any methods, additional references, Nature Portfolio reporting summaries, source data, extended data, supplementary information, acknowledgements, peer review information; details of author contributions and competing interests; and statements of data and code availability are available at 10.1038/s41565-026-02187-0.

## Supplementary information

Supplementary Information Supplementary Figs. 1–93, Tables 1–6 and Notes 1–7.

Reporting Summary

## Source data

Source Data Fig. 2 Statistical source data for Fig. 2.

Source Data Fig. 3 Statistical source data for Fig. 3.

Source Data Fig. 4 Statistical source data for Fig. 4.

Source Data Fig. 5 Statistical source data for Fig. 5.

Source Data Fig. 6 Statistical source data for Fig. 6.

## Data Availability

All data supporting the findings of this study are available in the Article and its Supplementary Information. Source data are provided with this paper.
