# Supplementary material for: Hydrophobic liquid electrolyte interphases for efficient aqueous zinc batteries
Source: Nat Nanotechnol. 2026 Jun 1;21(7):967–75. doi: 10.1038/s41565-026-02187-0 (PMC13379314; doi:10.1038/s41565-026-02187-0)
Supplement: Supplementary file 1 — Supplementary Figs. 1–93, Tables 1–6 and Notes 1–7. [file 41565_2026_2187_MOESM1_ESM.pdf]

nature nanotechnology

Supplementary information https://doi.org/10.1038/s41565-026-02187-0

Hydrophobic liquid electrolyte interphases for efficient aqueous zinc batteries

In the format provided by the authors and unedited

Table of Contents

Supplementary Figures 1 to 93

Supplementary Tables 1 to 6

Supplementary Notes 1 to 7

References

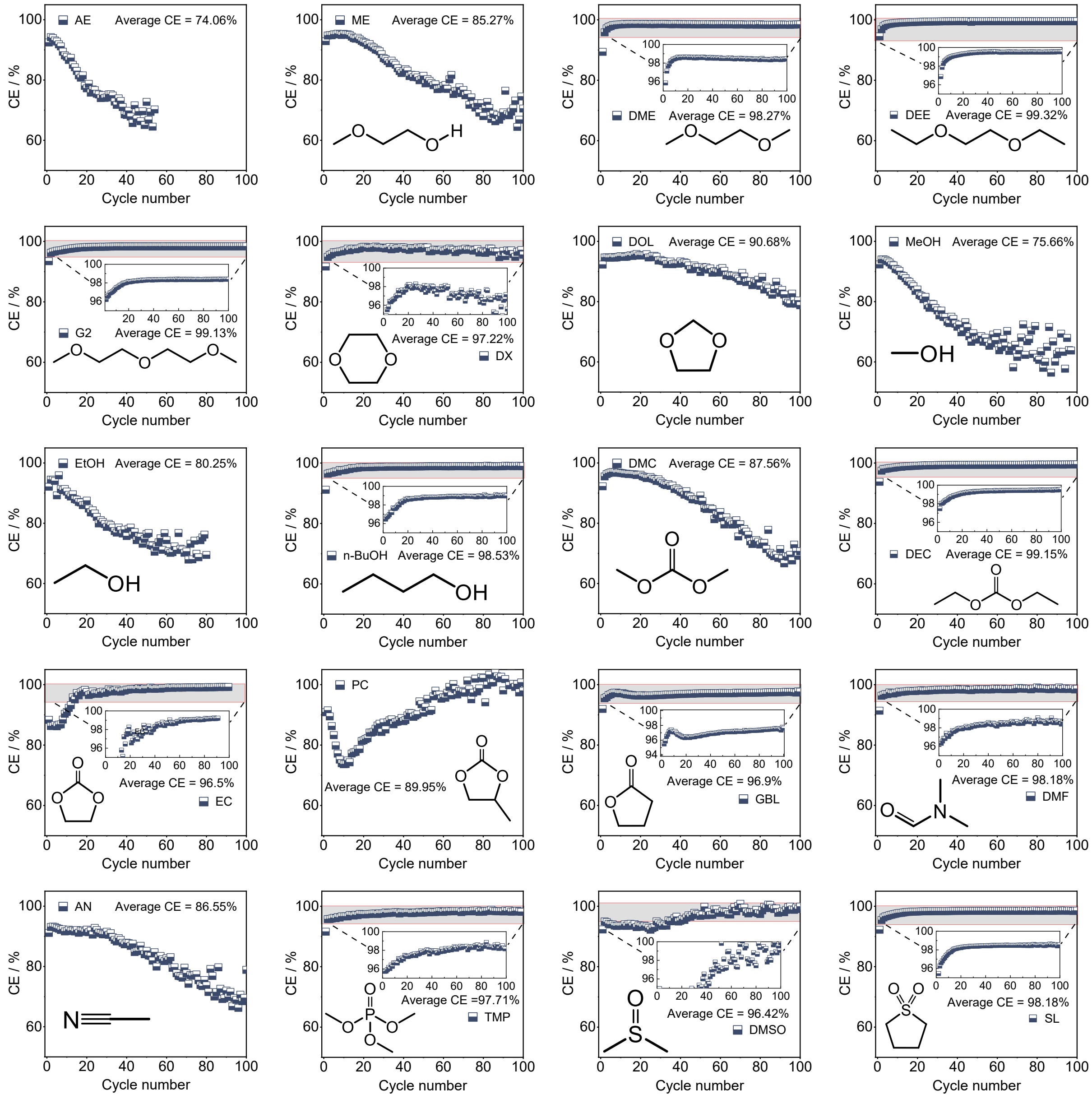

Supplementary Figure 1. Coulombic efficiency (CE) of asymmetric Zn||Cu cells tested at 1 mA cm^{-2}, 1 mA h cm^{-2} and 25 °C using 3 m Zn(OTf)$_2$ electrolyte solutions containing various organic additives at a concentration of 1.8 mol% for a single organic additive. The Zn(OTf)$_2$ was chosen for its strong salting-in effect, which enhances the miscibility of low-solubility organic solvents, such as DEE, beyond its intrinsic water solubility limit in the aqueous electrolyte solution (Supplementary Figs. 8 and 9). Abbreviations of electrolytes and solvents are as follows: 3m Zn(OTf)$_2$ in H_2O (AE), 2-methoxyethanol (ME), 1, 2-dimethoxyethane (DME), 1, 2-diethoxyethane (DEE), diglyme (G2), 1, 4-dioxane (DX), 1, 3-dioxolane (DOL), methanol (MeOH), ethanol (EtOH), 1-butanol (*n*-BuOH), dimethyl carbonate (DMC), diethyl carbonate (DEC), ethylene carbonate (EC), propylene carbonate (PC), 1, 4-butyrolactone (GBL), N, N-dimethylformamide (DMF), acetonitrile (AN), trimethyl phosphate (TMP), dimethyl sulfoxide (DMSO) and sulfolane (SL).

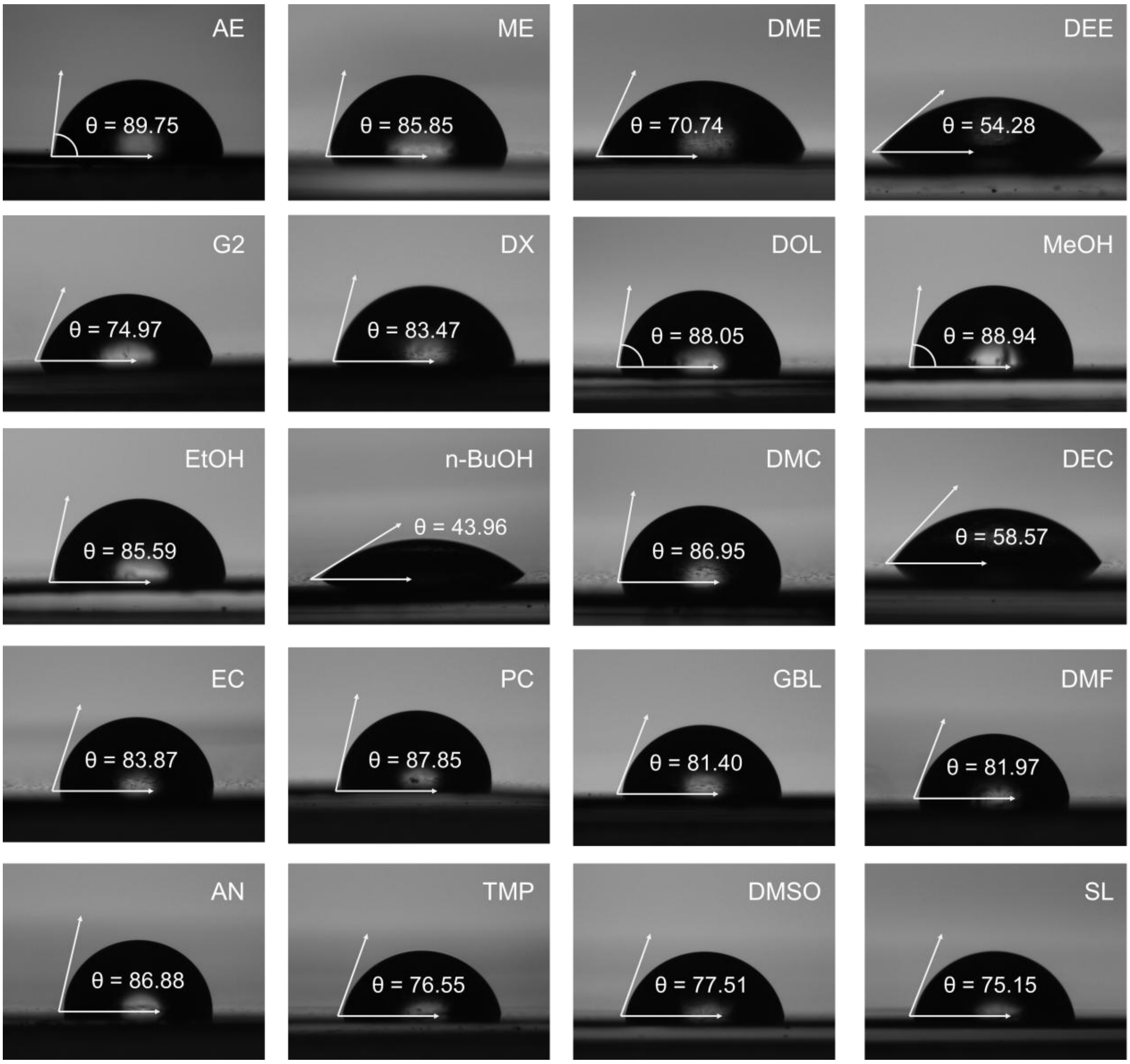

Supplementary Figure 2. Photographic pictures showing the contact angle between a Zn metal electrode and 3 m $Zn(OTf)_2$ electrolyte solutions containing various organic additives at a concentration of 1.8 mol% for a single organic additive.

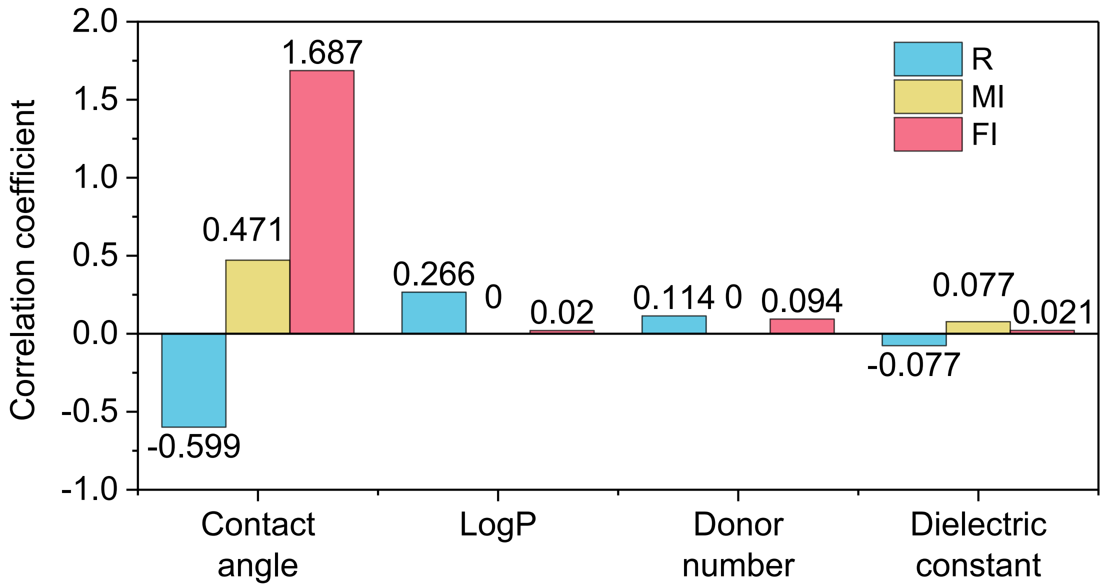

Supplementary Figure 3. Correlation coefficient between CE of asymmetric Zn||Cu cells with 3 m $Zn(OTf)_2$ electrolyte solutions containing selected organic additives and the four descriptors of organic additives, as proposed by three different statistical models: Pearson correlation coefficient (R), mutual information (MI) and permutational feature importance (FI).

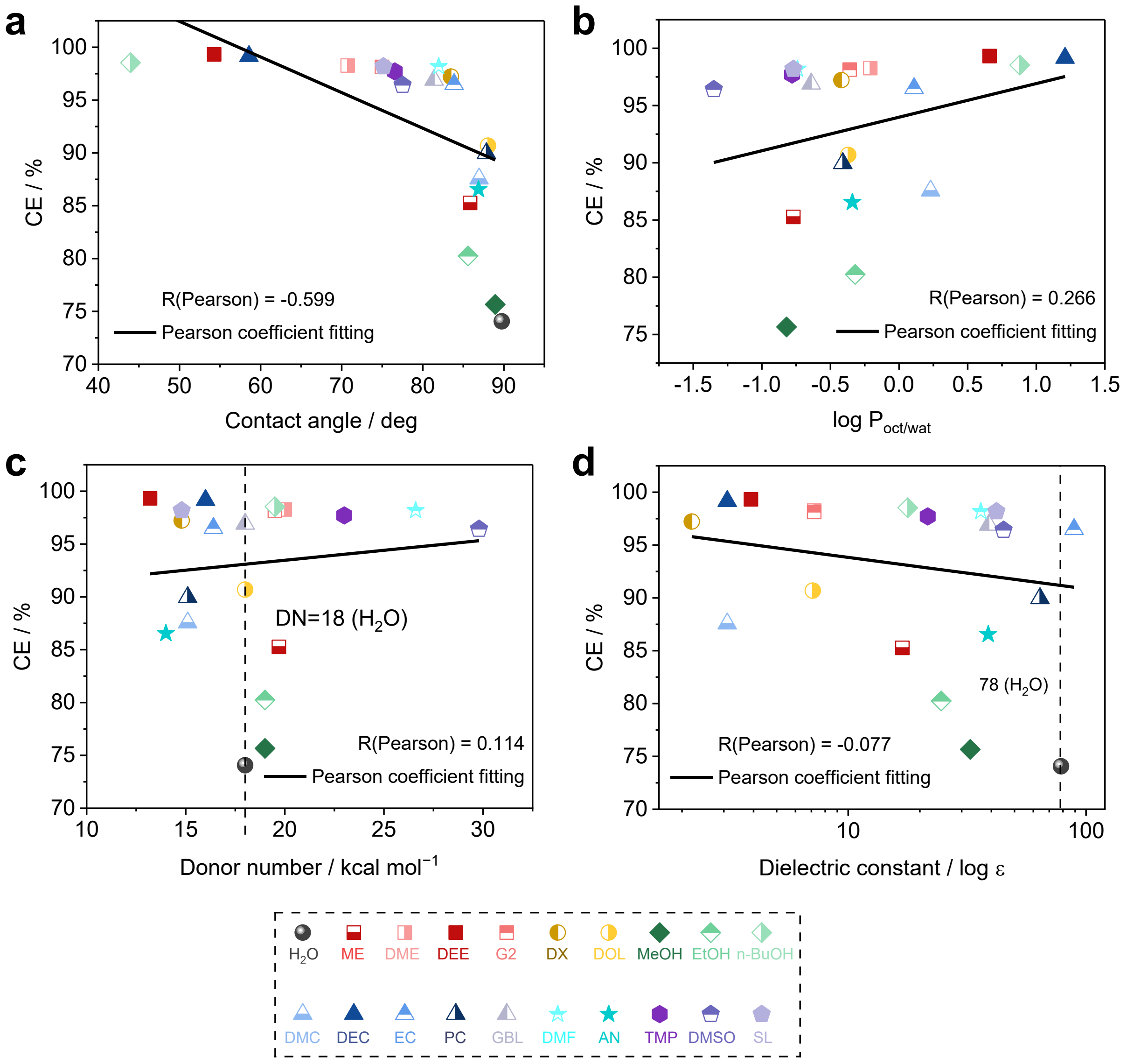

Supplementary Figure 4. Correlation between CE of asymmetric Zn||Cu cells with 3 m $Zn(OTf)_2$ electrolyte solutions containing various organic additives at a concentration of 1.8 mol% for a single organic additive and the four descriptors of the organic additives: (**a**) contact angle, (**b**) log $P_{oct/wat}$, (**c**) donor number and (**d**) dielectric constant.

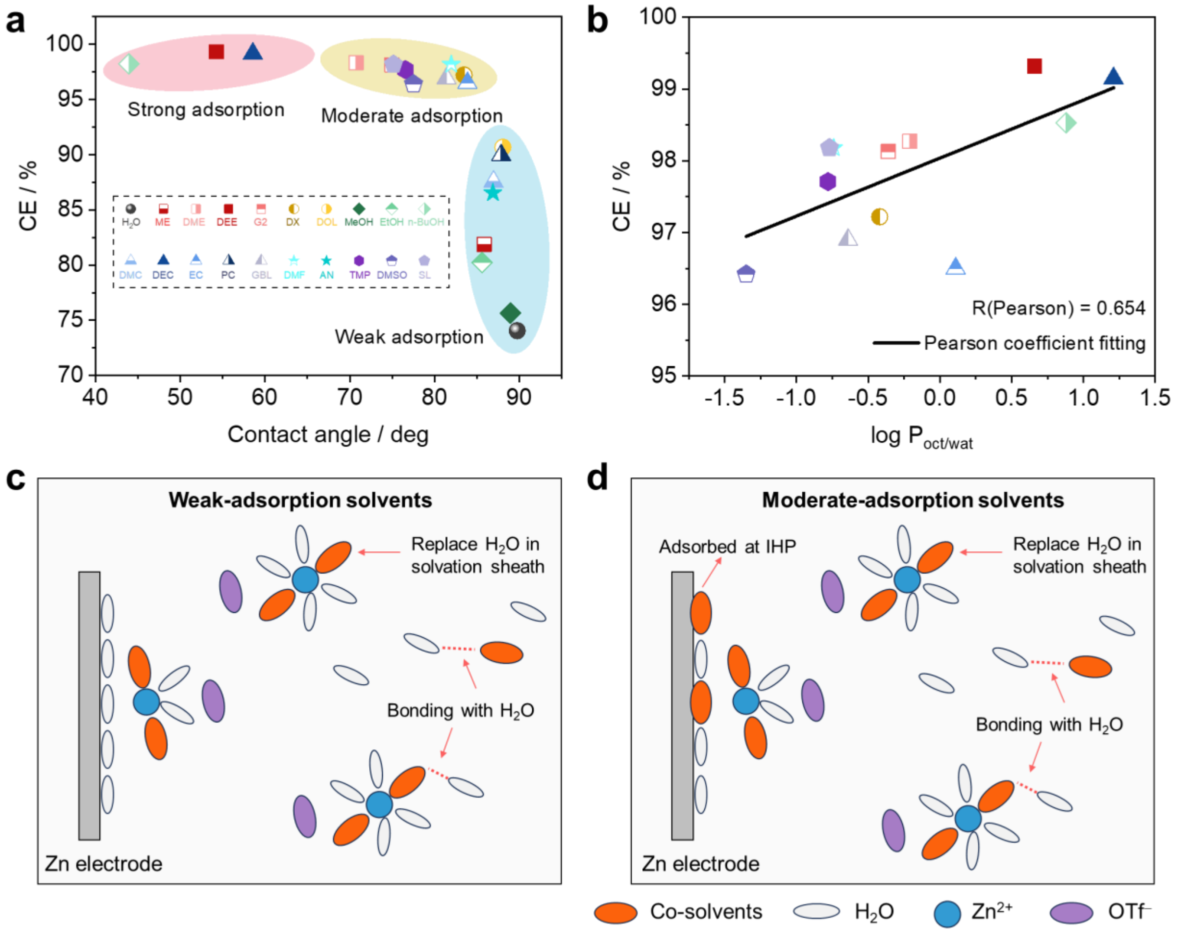

Supplementary Figure 5. (**a**) Correlation coefficient between CE of asymmetric Zn||Cu cells with 3 m Zn(OTf)$_2$ electrolyte solutions containing various organic additives at a concentration of 1.8 mol% for a single organic additive and the contact angle. Solvents are categorized into three zones: weak-adsorption (zone I), moderate-adsorption (zone II) and strong-adsorption (zone III). (**b**) Correlation coefficient between CE of asymmetric Zn||Cu cells with 3 m Zn(OTf)$_2$ electrolyte solutions containing various organic additives at a concentration of 1.8 mol% for a single organic additive and the log $P_{oct/wat}$ of organic solvents in zone II and zone III. Schematic representation of the HER mitigation mechanism of solvents in (**c**) zone I and (**d**) zone II.

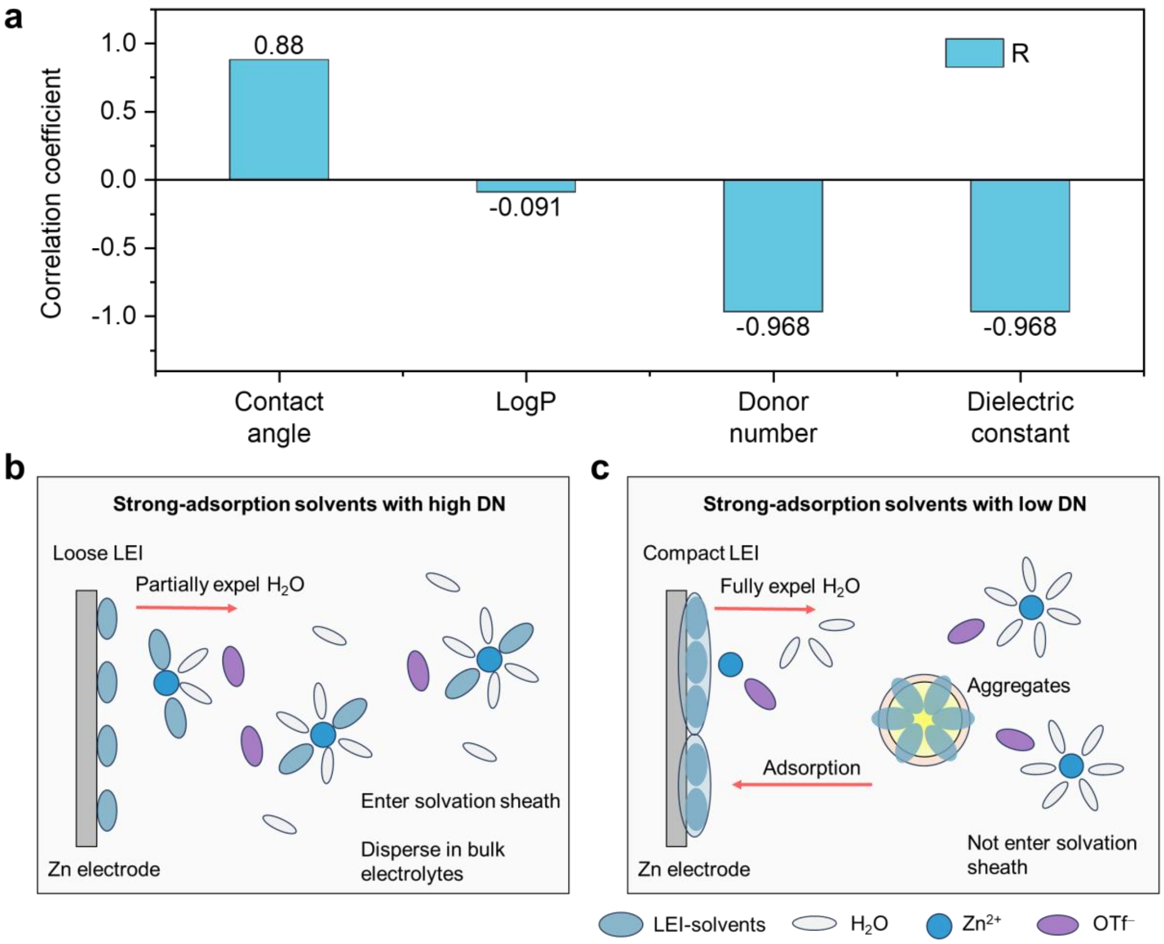

Supplementary Figure 6. (**a**) Correlation coefficient between CE of asymmetric Zn||Cu cells with 3 m $Zn(OTf)_2$ electrolyte solutions containing various organic additives at a concentration of 1.8 mol% for a single organic additive and the four descriptors of the organic additives in zone III. Schematic representation of the HER mitigation mechanism of solvents with (**b**) strong solvation ability and (**c**) weak solvation ability. The yellow-filled, orange-bordered circle in panel c denotes a DEE cluster composed of aggregated DEE molecules (blue). Noted, only the Pearson coefficient was calculated here due to the limited number of solvents (three), which would cause statistical reliability issues when using MI or FI models.

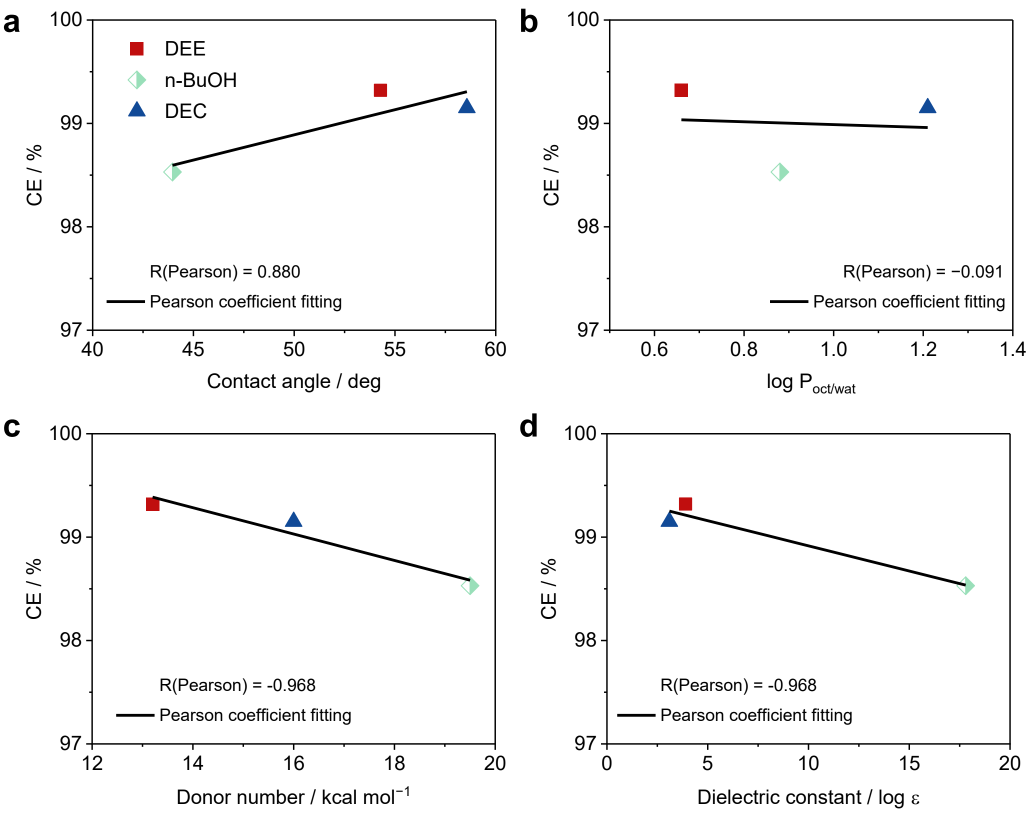

Supplementary Figure 7. Correlation coefficient between CE of asymmetric Zn||Cu cells with 3 m $Zn(OTf)_2$ electrolyte solutions containing various organic additives at a concentration of 1.8 mol% for a single organic additive and the four descriptors of the organic additives in zone III: (**a**) contact angle, (**b**) log $P_{oct/wat}$, (**c**) donor number and (**d**) dielectric constant.

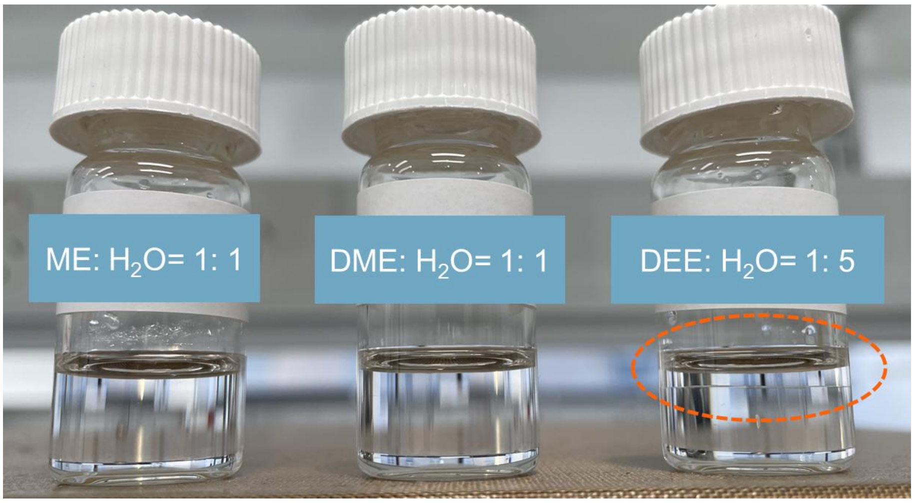

Supplementary Figure 8. Photographic pictures of the vials containing the various ether/H_2O binary mixtures (without salt) at 25 °C in air, giving qualitative insights into the solubility of the ethers in water. It can be noticed that both ME and DME are miscible with H_2O, whereas phase separation occurs when the DEE: H_2O weight ratio reaches 1: 5, indicating the low water solubility and high hydrophobicity of DEE.

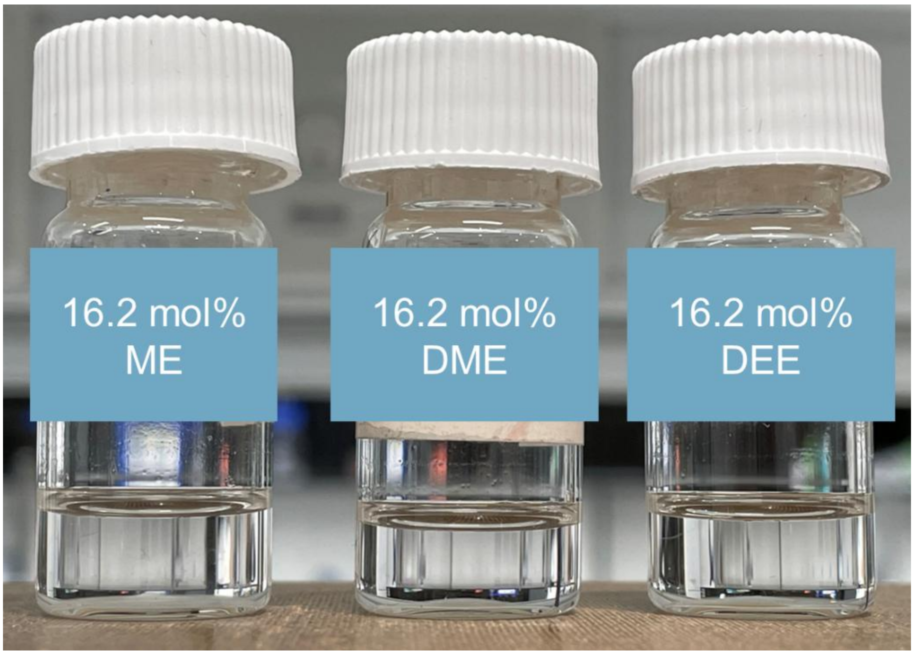

Supplementary Figure 9. Optical photos of the 3 mol kg^{-1} (m) $Zn(OTf)_2$ electrolyte solutions with selected ether additives at 25 °C in air. When the $Zn(OTf)_2$ salt is dissolved in water, the solubility of DEE in water is enhanced due to the chaotropic effect of the OTf^- anion, whose weak hydration ability breaks water-water hydrogen bonding and promotes dissolution of the organic additives. Particularly, DEE is macroscopically miscible in the 3 m $Zn(OTf)_2$ aqueous electrolyte solution even at 16.2 mol% (equal to a DEE: H_2O weight ratio of 1.06: 1).

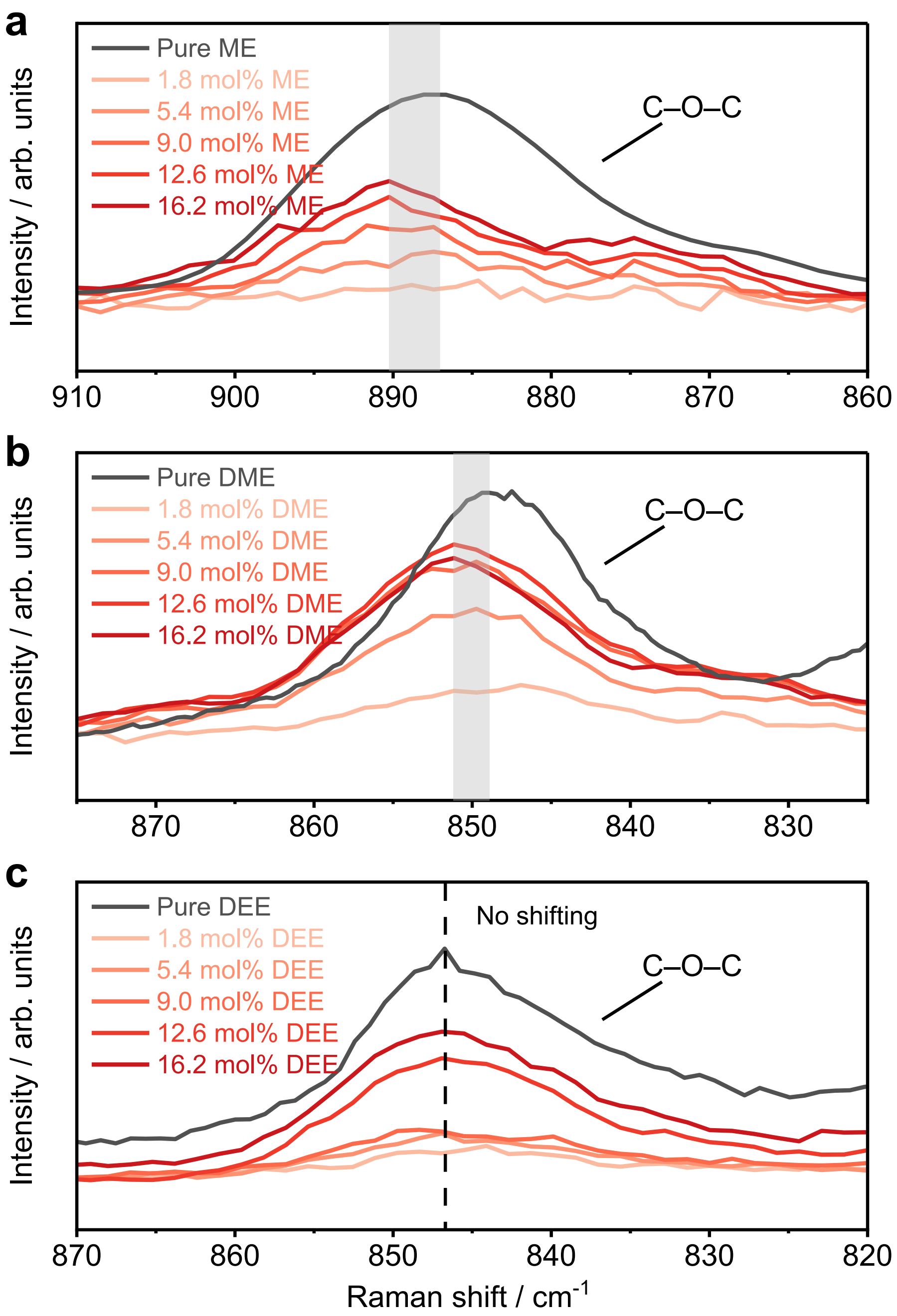

Supplementary Figure 10. Raman spectra of 3 m $Zn(OTf)_2$ aqueous electrolyte solutions containing various amounts of organic additives: (**a**) ME, (**b**) DME and (**c**) DEE, which indicates the ν_s(C–O–C) shifting of ethers. Raman spectra revealed that the C–O–C symmetric stretching vibrations of DEE molecules did not shift in electrolytes containing DEE ranging from 1.8 to 16.2 mol%, in contrast to the shifting to high wavenumbers observed in electrolyte solutions containing ME and DME additives. This observation suggests that DEE molecules do not solvate Zn^{2+} ions but remains as a cation-uncoordinated electrolyte component outside the solvation sheath due to its weak solvating ability. Moreover, the weak solvation ability and hydrophobicity of cation-uncoordinated DEE molecules promote self-coordination, leading to the formation of DEE aggregates that resemble a micellar structure. This structure arises from the surfactant-like physicochemical behaviour of DEE molecules, which contain hydrophobic tails ($-CH_2CH_3$) for self-aggregation and hydrophilic heads (–O–) that bind to water molecules.

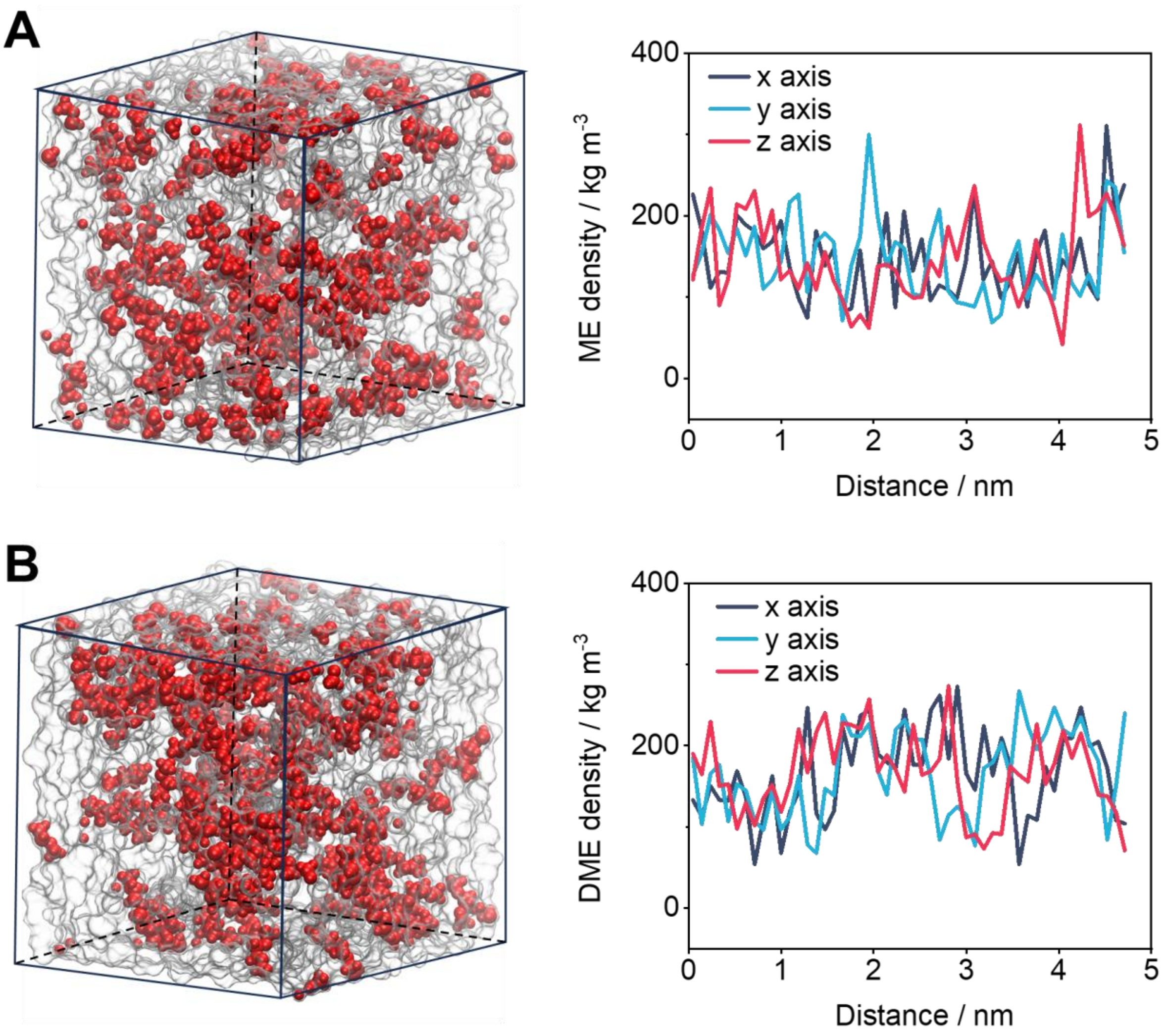

Supplementary Figure 11. The snapshot and density profiles of 3 m $Zn(OTf)_2$ aqueous electrolyte solutions containing (**a**) ME and (**b**) DME additives (1.8 mol%) across *x*, *y* and *z* axes in the MD simulation box, showing the uniform spatial distribution of these two molecules.

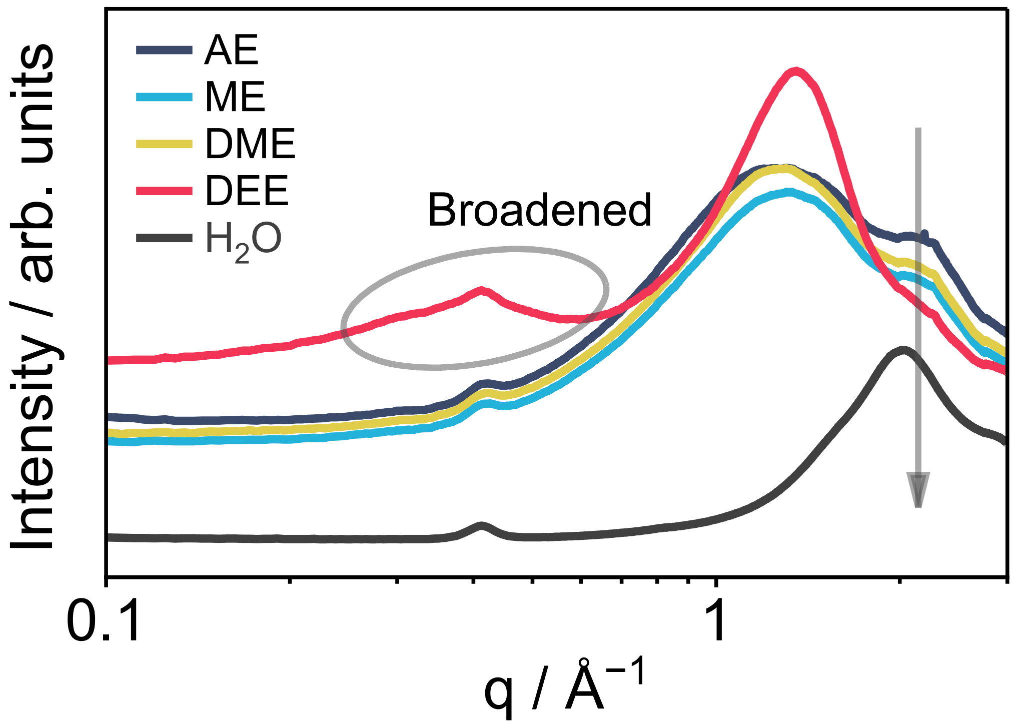

Supplementary Figure 12. SAXS spectra of ultrapure water (approximately 18.2 MΩ cm at 25 °C) and 3 m $Zn(OTf)_2$ aqueous electrolyte solutions containing ME, DME and DEE organic additives (1.8 mol%).

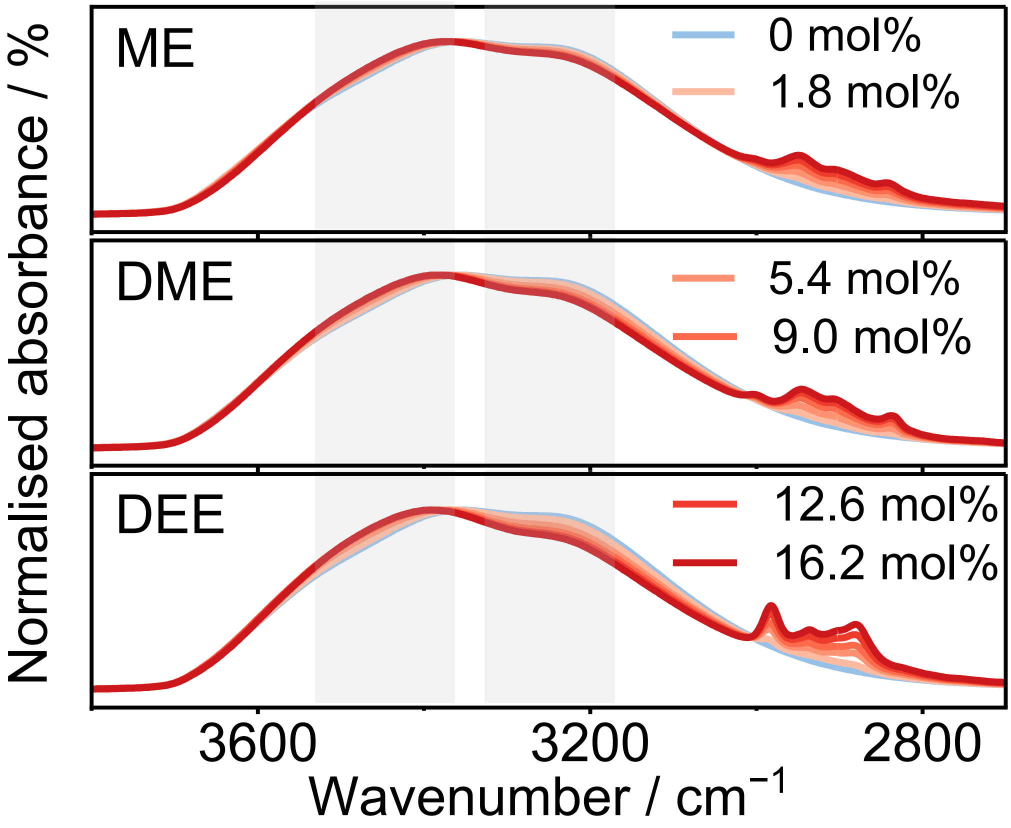

Supplementary Figure 13. FTIR spectra of 3 m $Zn(OTf)_2$ aqueous electrolyte solutions containing ME, DME and DEE organic additives in various concentrations, which show the ν_s(O–H) shift of H_2O molecules.

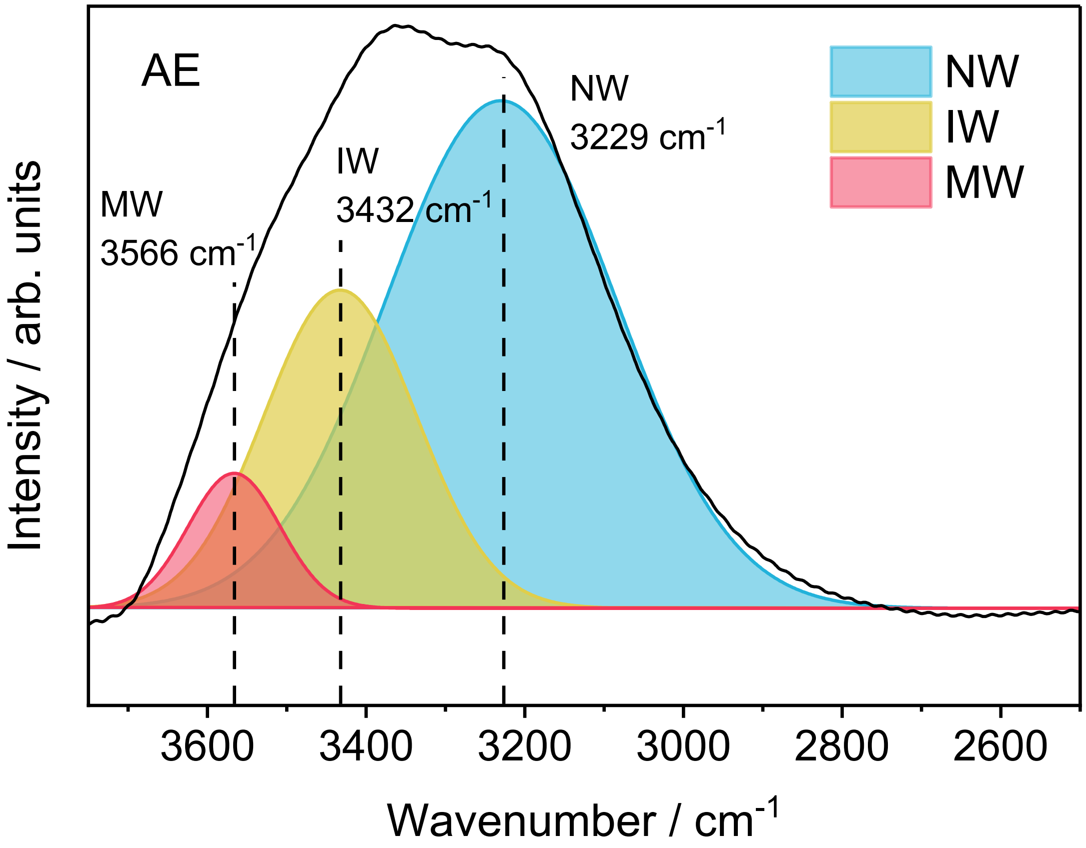

Supplementary Figure 14. Deconvolution analysis of the O–H stretching vibration in the baseline AE solution (3 m $Zn(OTf)_2$) showing the positions of various water cluster structures.

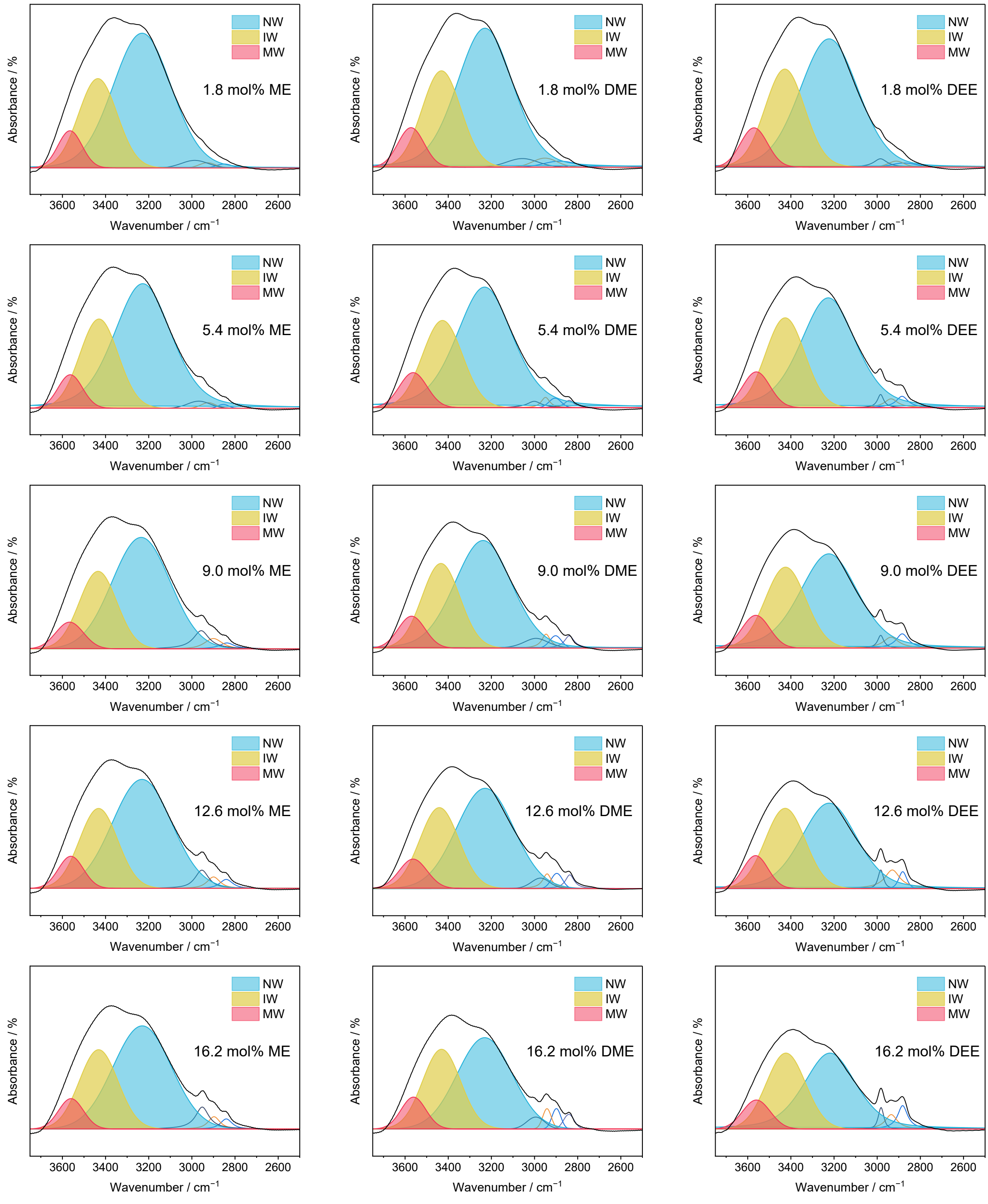

Supplementary Figure 15. Deconvolution analysis of the O–H stretching vibration in 3 m $Zn(OTf)_2$ electrolyte solutions containing ME, DME and DEE organic additives at different concentrations.

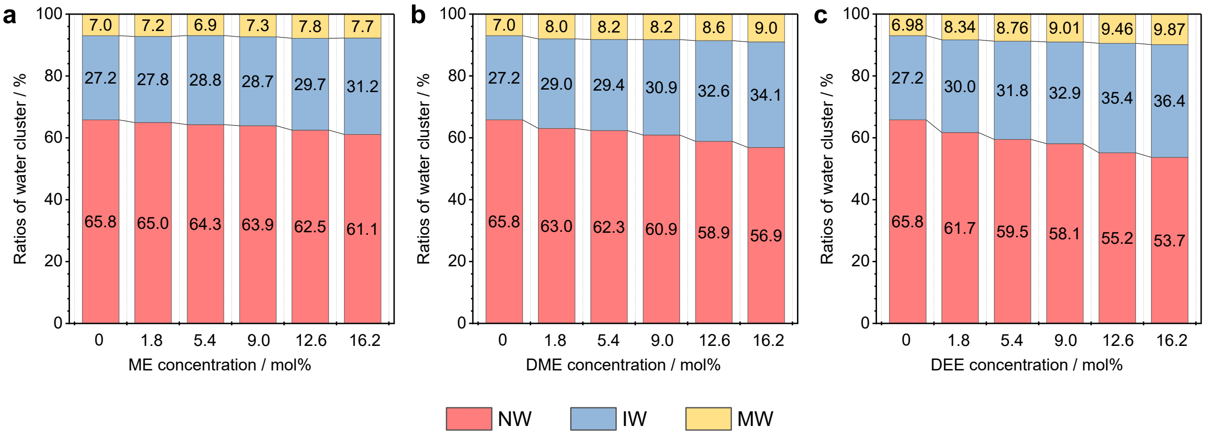

Supplementary Figure 16. Ratios of different types of water molecule clusters in 3 m $Zn(OTf)_2$ electrolyte solutions containing various concentrations of (**a**) ME, (**b**) DME and (**c**) DEE organic additives. Results are obtained from the deconvolution analysis of the O–H stretching region in Supplementary Fig. 15.

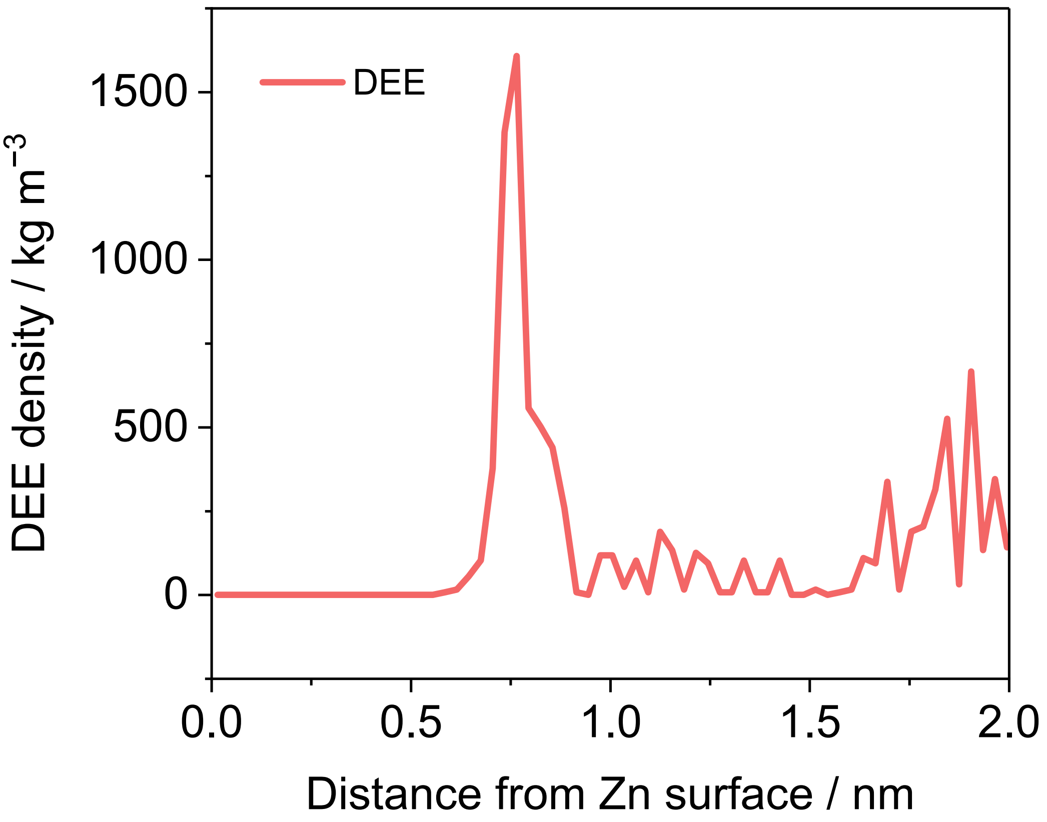

Supplementary Figure 17. Density profile of DEE molecules as a function of the distance from the Zn metal electrode surface. Here, to observe the adsorption behaviours of DEE molecules as micelles on the Zn electrode surface, we conducted MD simulations of DEE molecules in contact with the Zn metal (101) facet. The density of DEE at the Zn surface reaches ~1600 kg m^{-3}, which is four-time higher than the ~400 kg m^{-3} in the bulk of the electrolyte solution (Fig. 3b). This indicates that the DEE micelles already formed in the bulk of the electrolyte solution further aggregates on the Zn metal surface, driven by the strong adsorption tendency of DEE molecules, ultimately forming a compact DEE-LEI layer.

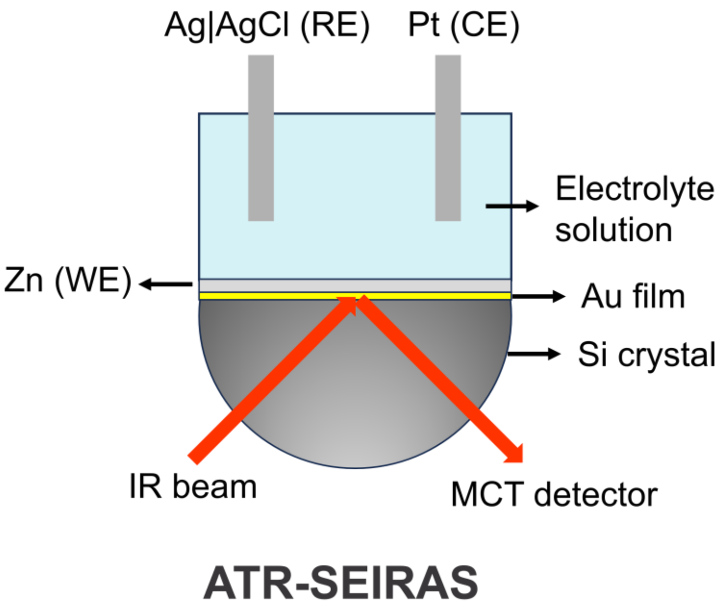

Supplementary Figure 18. Schematic diagram of the ATR-SEIRAS experimental cell setup. Reference electrode: RE; Counter electrode: CE; Working electrode: WE; Mercury Cadmium Telluride: MCT. The ATR-SEIRAS technique is effective in amplifying IR signals from surface species, particularly those within about 5 nm of an interface[1], which allows for the detection of absorption anions with ion radii on the order of a few Ångströms. The maximum detection depth of this setup is typically a few nanometres, corresponding to the evanescent-wave penetration depth, which is determined by the refractive index of the ATR crystal and the incident angle. To ensure reliable detection of interfacial signals in our experiments, we optimized several key aspects of the configuration. A thin (~10 nm) Au film was sputtered onto a high-refractive-index Si ATR crystal to provide both strong surface enhancement and good electrical conductivity. The incidence angle was set to ~45°, which allows the evanescent field to effectively probe the nanometre-thick interfacial region. In addition, the electrolyte solution layer was confined to about 20 μm by adjusting the spacing between the crystal and the bottom of the cell, which minimizes bulk absorption and maximizes the contribution of interfacial species. With this design, the ATR-SEIRAS technique selectively amplifies vibrational signals from species at the electrode-electrolyte interface, enabling high-surface-sensitivity monitoring of the adsorption and transformation of reaction intermediates.

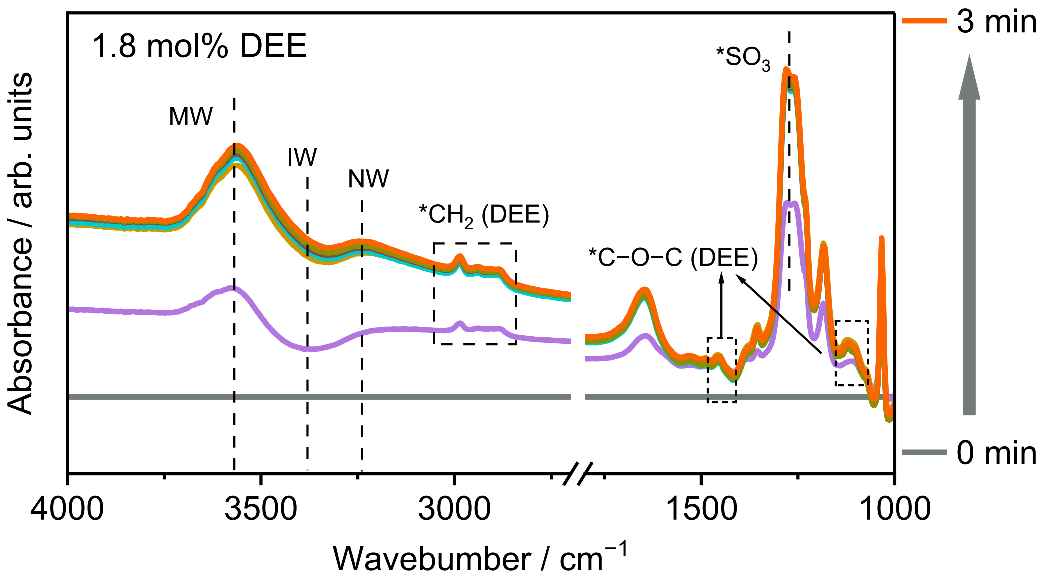

Supplementary Figure 19. *In situ* SEIRAS spectra of surface species on the Zn negative electrode surface for a 3 m $Zn(OTf)_2$ electrolyte solution with 1.8 mol% DEE, illustrating the progressive adsorption of DEE molecules on the Zn surface. The test was conducted without applying an external electric field. These ATR-SEIRAS spectra show that the characteristic signals of DEE start to appear within 30 seconds after electrolyte solution injection in the cell, indicating the onset of DEE adsorption on the Zn metal electrode surface. The intensity of these signals gradually increases and reaches a steady state after about one minute, remaining nearly unchanged thereafter. This behaviour suggests that the adsorption of DEE molecules rapidly reaches equilibrium within a few minutes, suggesting the establishment of a stable interfacial layer.

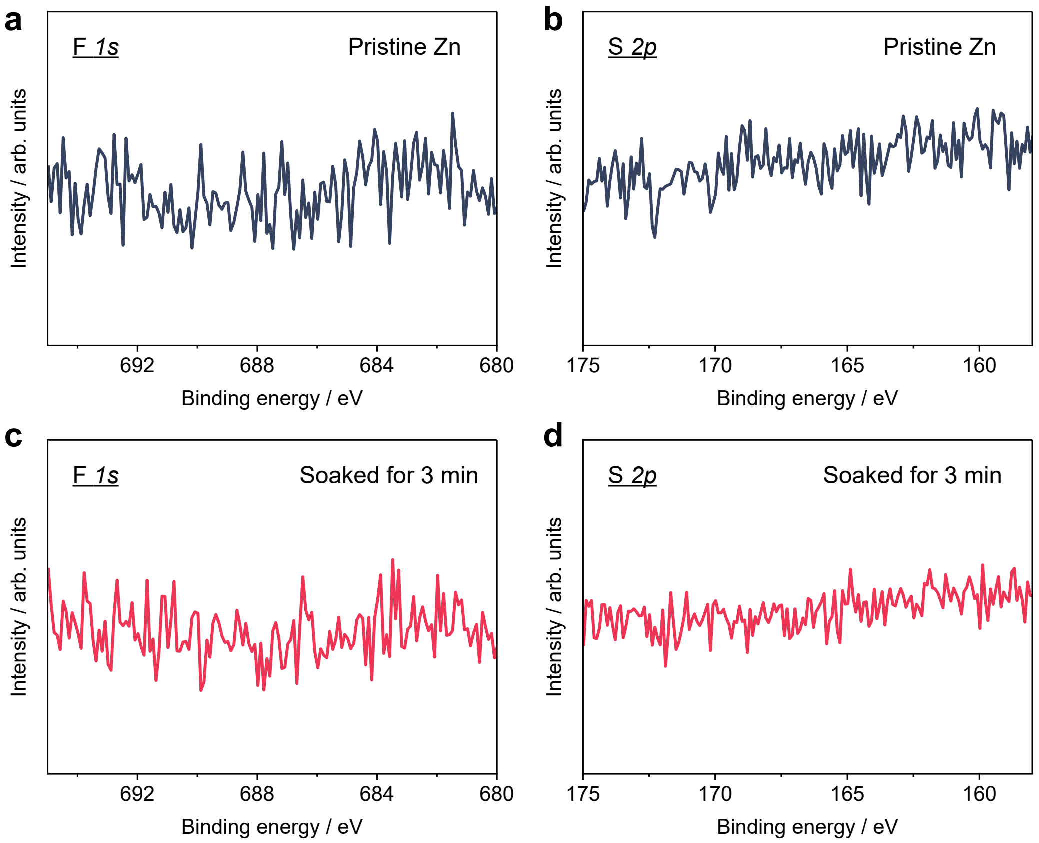

Supplementary Figure 20. XPS spectra of Zn metal electrodes before and after soaking in 3 m $Zn(OTf)_2$ electrolyte solution with 1.8 mol% DEE for 3 min: (**a**) F *1s* and (**b**) S *2p* pf the Zn metal electrode prior to soaking, (**c**) F *1s* and (**d**) S *2p* of the Zn metal electrode after soaking.

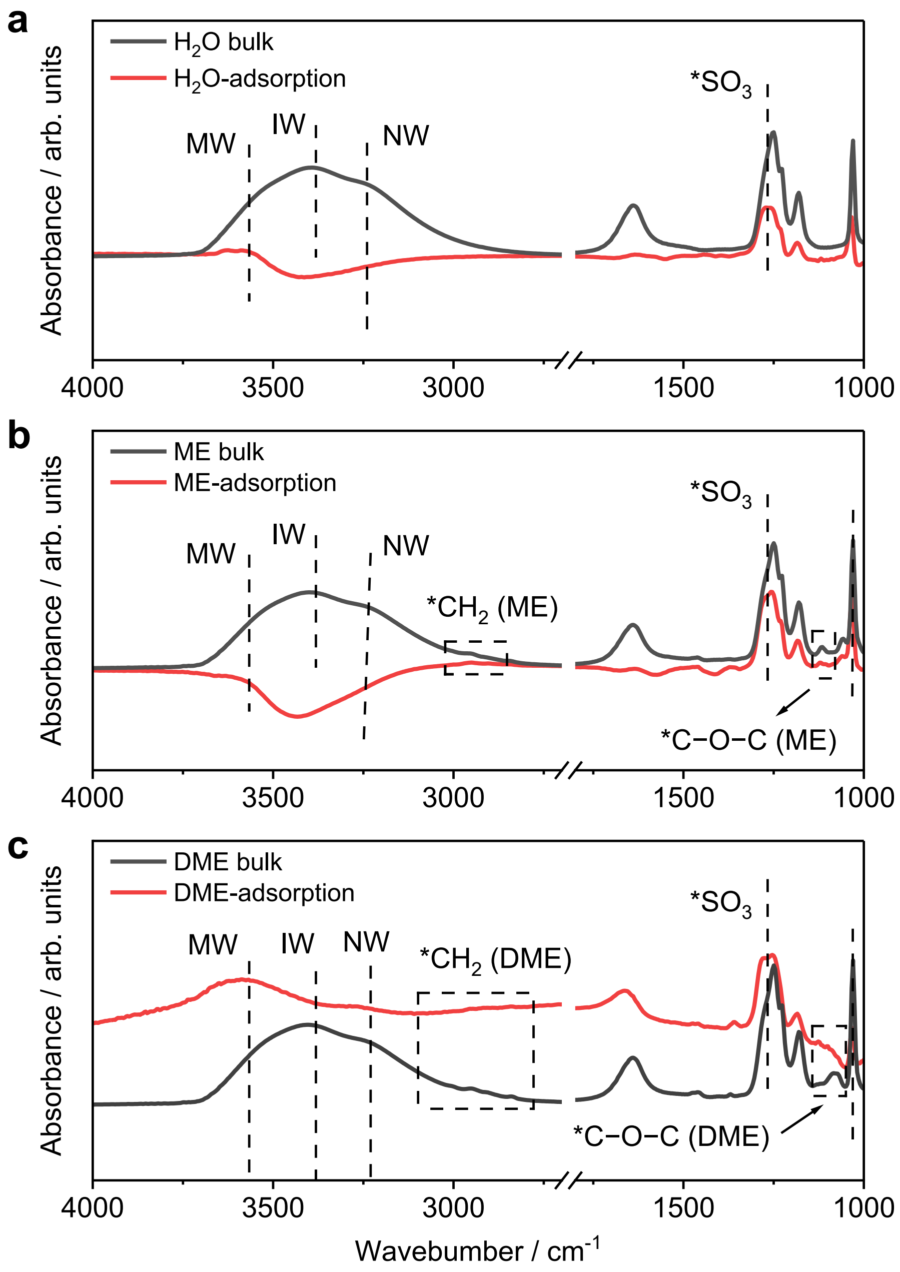

Supplementary Figure 21. ATR-SEIRAS spectra in the bulk and adsorbed at the Zn metal electrode surface of 3 m $Zn(OTf)_2$ electrolyte solutions (**a**) without additives (baseline AE), and with (**b**) 1.8 mol% ME and (**c**) 1.8 mol% DME additives. The spectra of bulk electrolytes were collected from samples without contact with the Zn electrode, while the spectra of adsorption electrolytes were derived from the electrolyte layer present on the Zn surface.

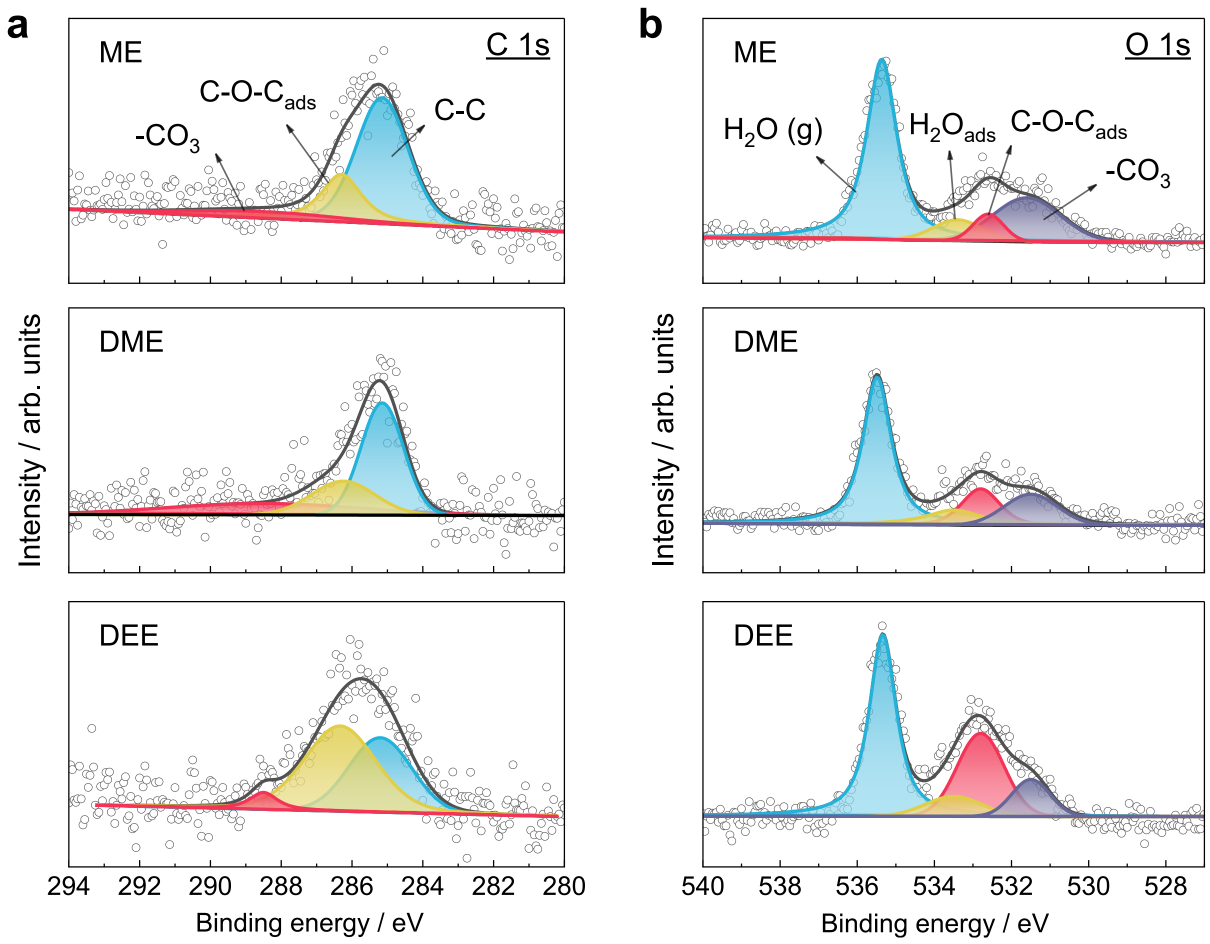

Supplementary Figure 22. NAP-XPS spectra collected ex situ for the 3 m $Zn(OTf)_2$ electrolyte solutions with 1.8 mol % of ME, DME and DEE additives adsorbed on the Zn metal electrode surface : (**a**) C 1*s* and (**b**) O 1*s*.

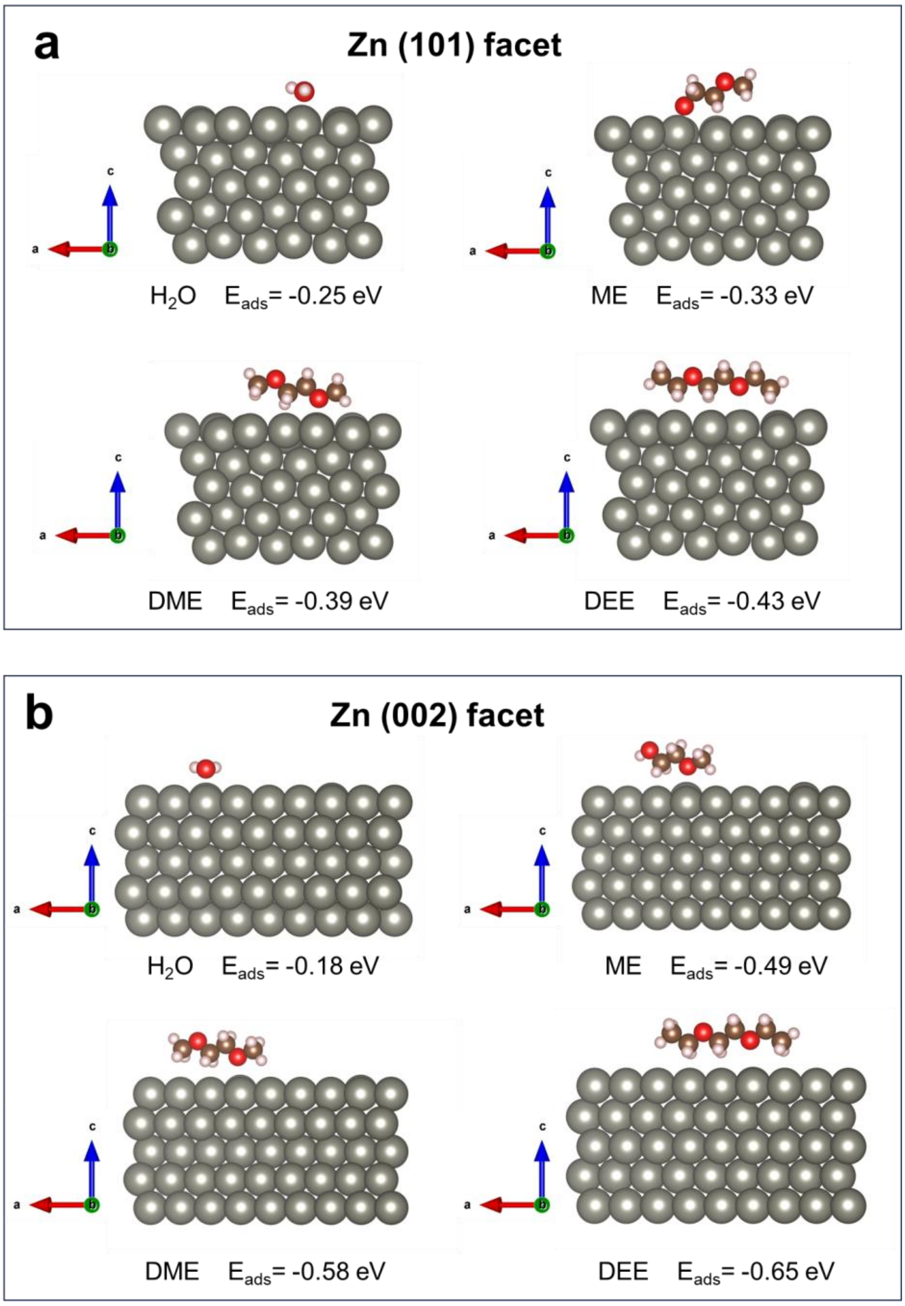

Supplementary Figure 23. Calculation results of adsorption energy of different solvents (H_2O, ME, DME and DEE) on preferred (**a**) Zn (101) and (**b**) Zn (002) facets.

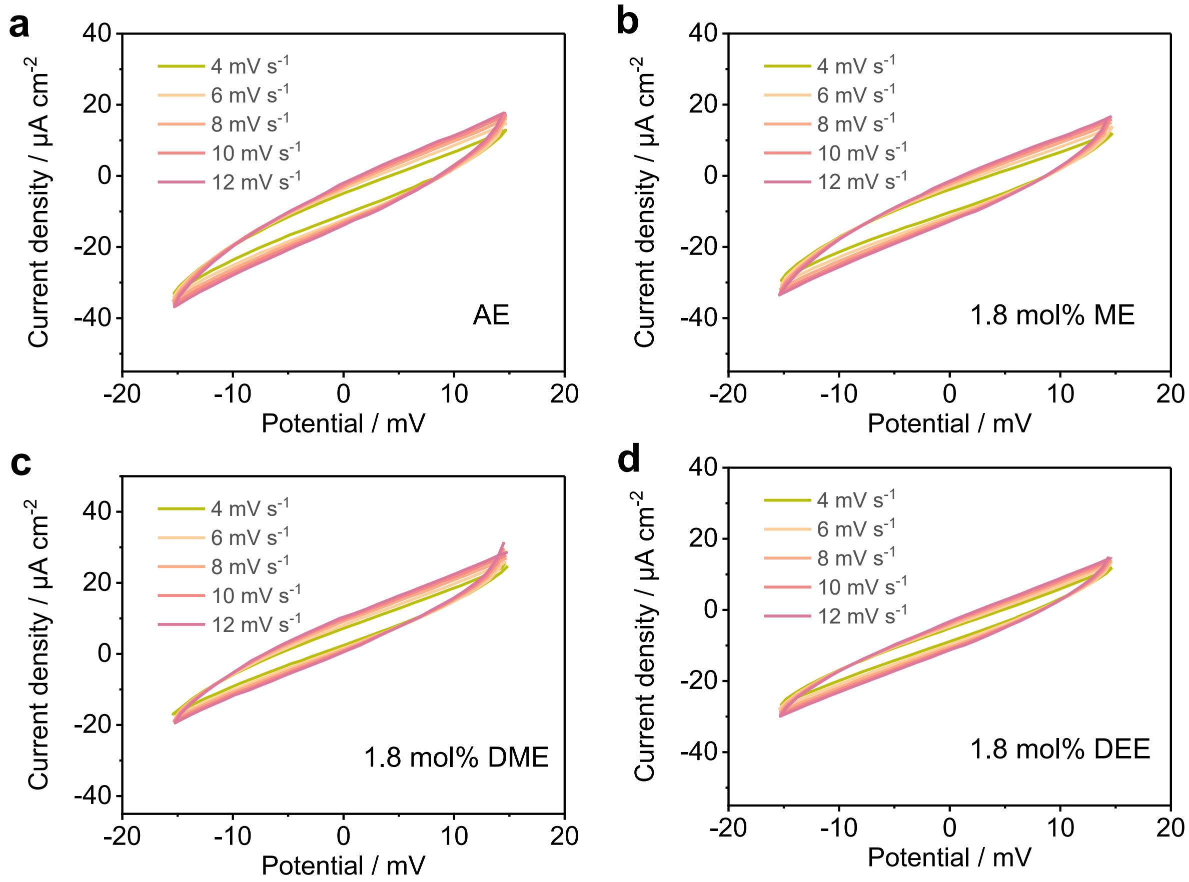

Supplementary Figure 24. Cyclic voltammetry (CV) curves of Zn||Zn cells in (**a**) AE, (**b**) 1.8 mol% ME, (**c**) 1.8 mol% DME and (**d**) 1.8 mol% DEE electrolyte solutions at scanning rates ranging from 4 to 12 mV s^{-1}.

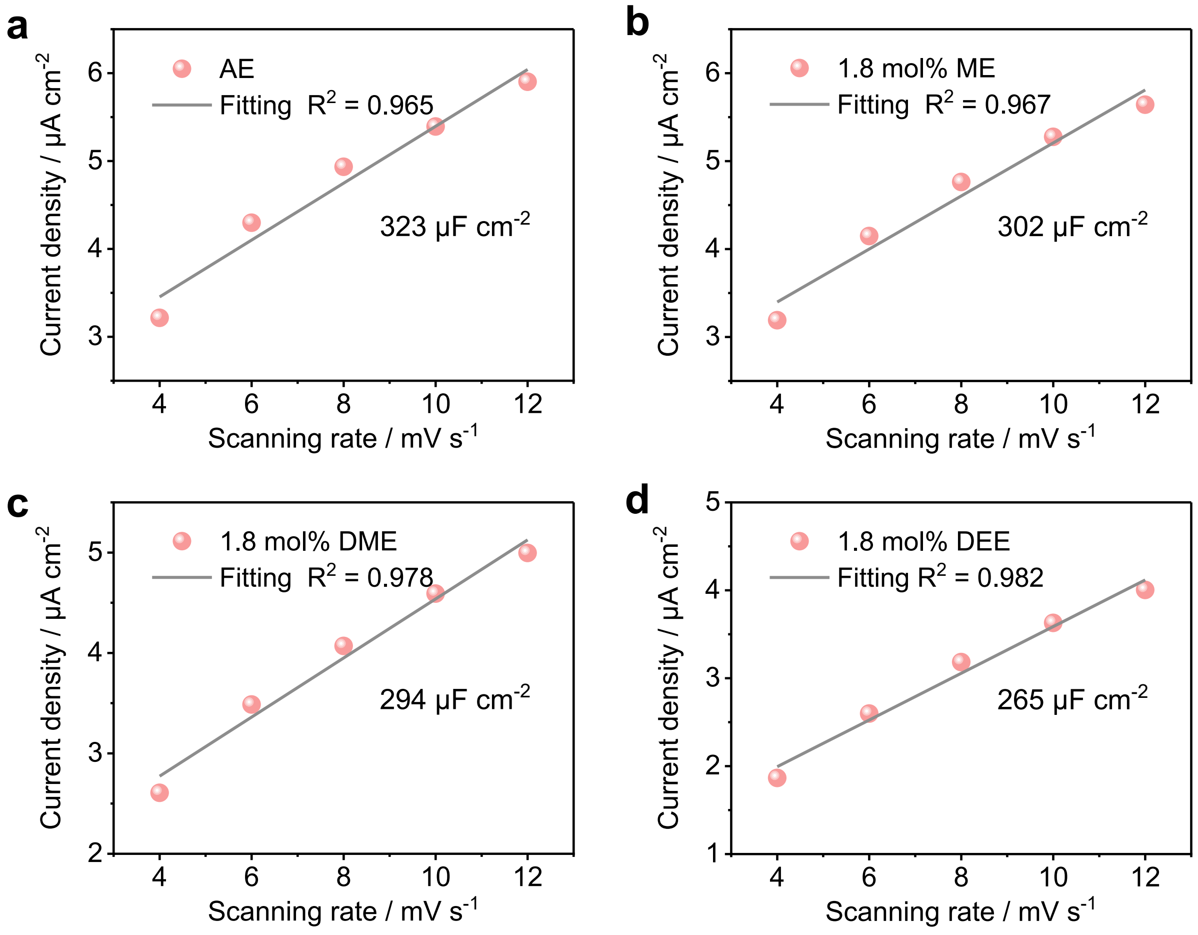

Supplementary Figure 25. Current density-scanning rate fitting result of Zn||Zn cells in (**a**) AE, (**b**) 1.8 mol% ME, (**c**) 1.8 mol% DME and (**d**) 1.8 mol% DEE electrolyte solutions, showing the capacitance (μF cm^{-2}) of the electric double layer (EDL). The inner Helmholtz layer (IHP) at the Zn|electrolyte interface typically consists of adsorbed water molecules in aqueous electrolyte solutions [2,3], which can be altered when organic molecules are added. The organic additive can replace the adsorbed water molecule in the IHP, thereby mitigating water splitting. This change in the IHP is reflected in a decrease in the EDL capacitance (C_{EDL}). The low dielectric constant of adsorbed ether molecules reduces the overall dielectric constant of the adsorption layer, resulting in a decrease in C_{EDL} according to the equation $C_{EDL} = \frac{\varepsilon A}{d}$ (Eq. 1), where ε is the dielectric constant of EDL, A is the electrode surface area and d is the thickness of the EDL. As a result, a lower value is observed in the DEE electrolyte, indicating that more DEE molecules replace H_2O and adsorb onto the Zn surface.

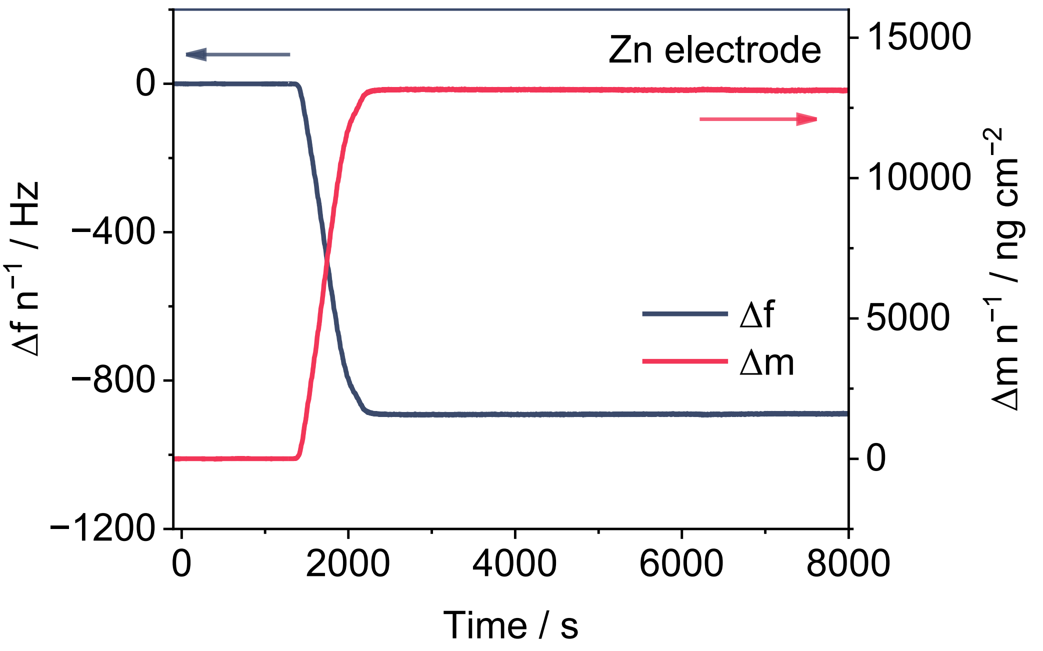

Supplementary Figure 26. QCM measurement (harmonic number n = 3) of the Zn negative electrode in the 1.8 mol% DEE electrolyte. Δf is the frequency change, and Δm is the mass change after the electrolyte injection. In the experiment, the frequency of the Zn electrode in AE, where water molecules serve as the primary adsorbed species, was first recorded as the baseline.

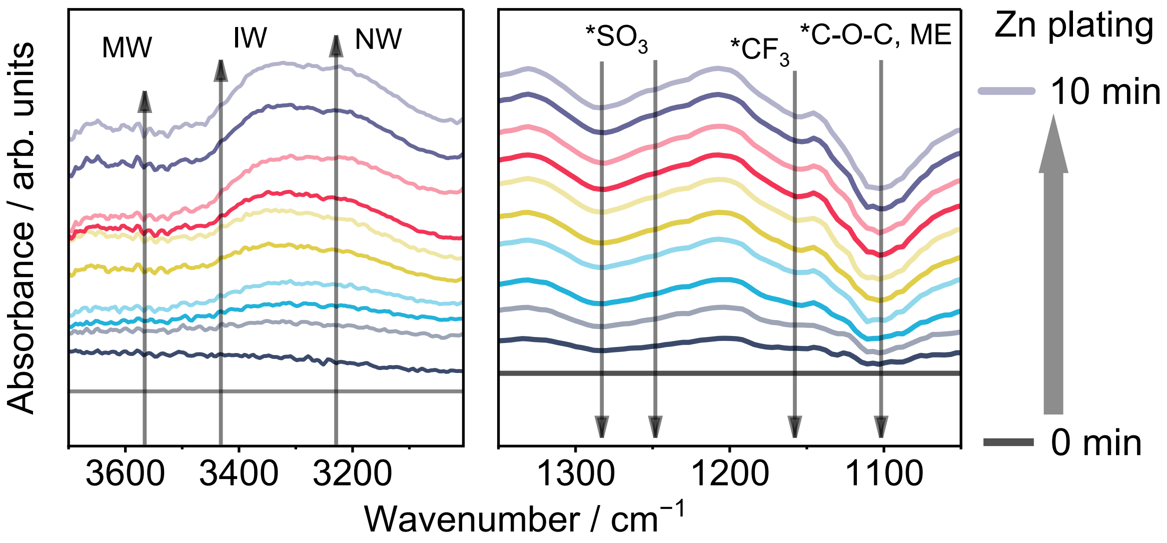

Supplementary Figure 27. *In situ* ATR-SEIRAS spectra of the 3 m Zn(OTf)$_2$ electrolyte solutions with 1.8 mol% of ME additive during the Zn metal electrodeposition.

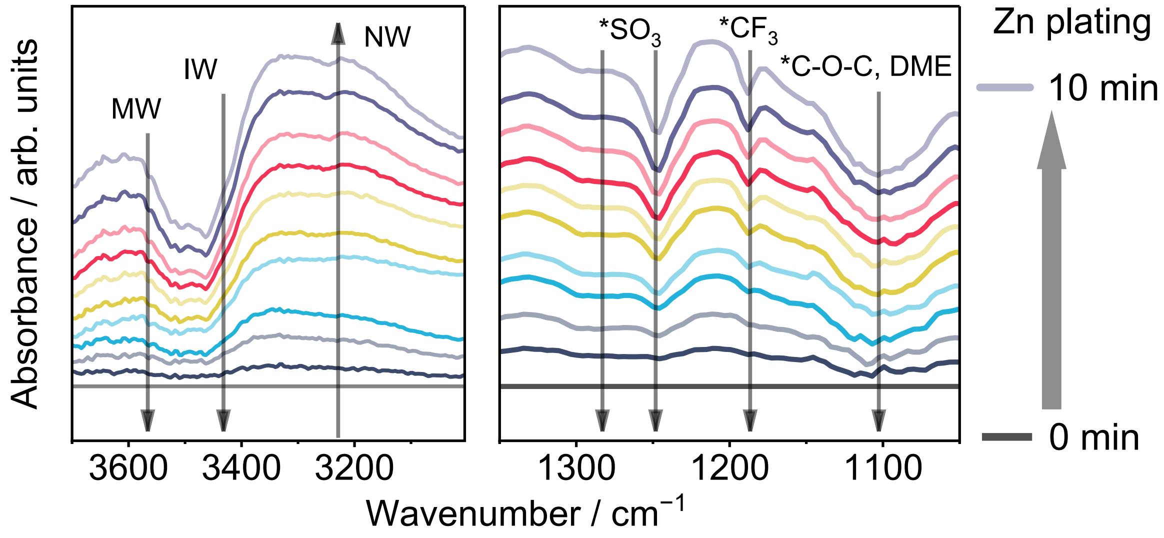

Supplementary Figure 28. *In situ* ATR-SEIRAS spectra of the 3 m Zn(OTf)$_2$ electrolyte solutions with 1.8 mol% of DME additive during the Zn metal electrodeposition.

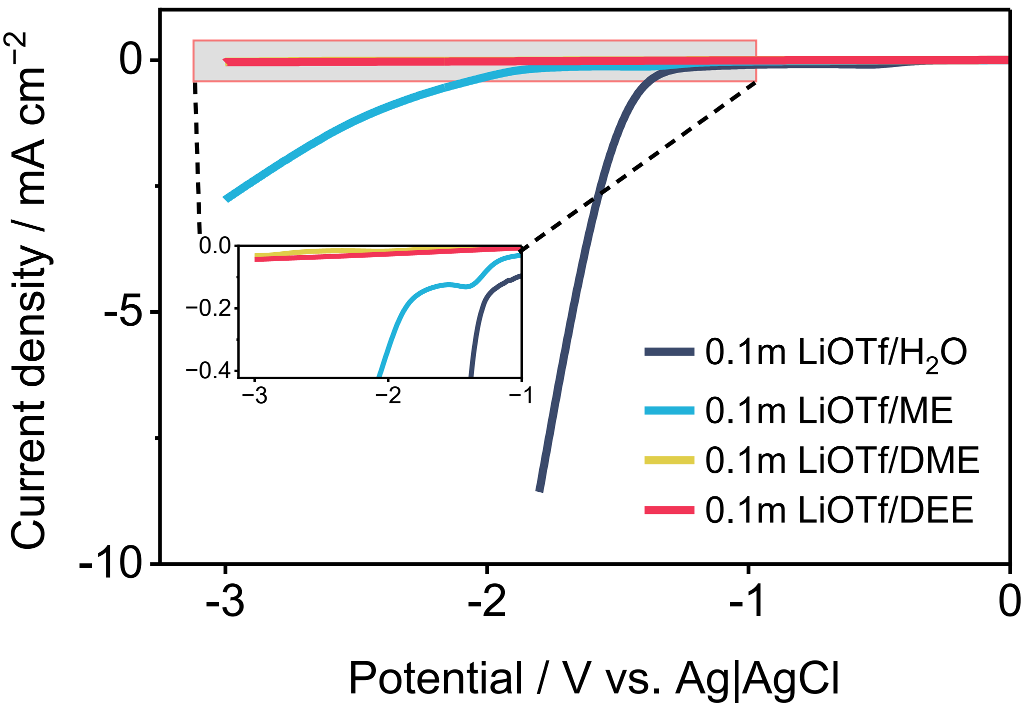

Supplementary Figure 29. Linear sweep voltammetry (LSV) measurements of Ti electrodes in various electrolyte solutions at a scanning of 5 mV s^{-1}, showing the reductive stability of water, ME, DME and DEE.

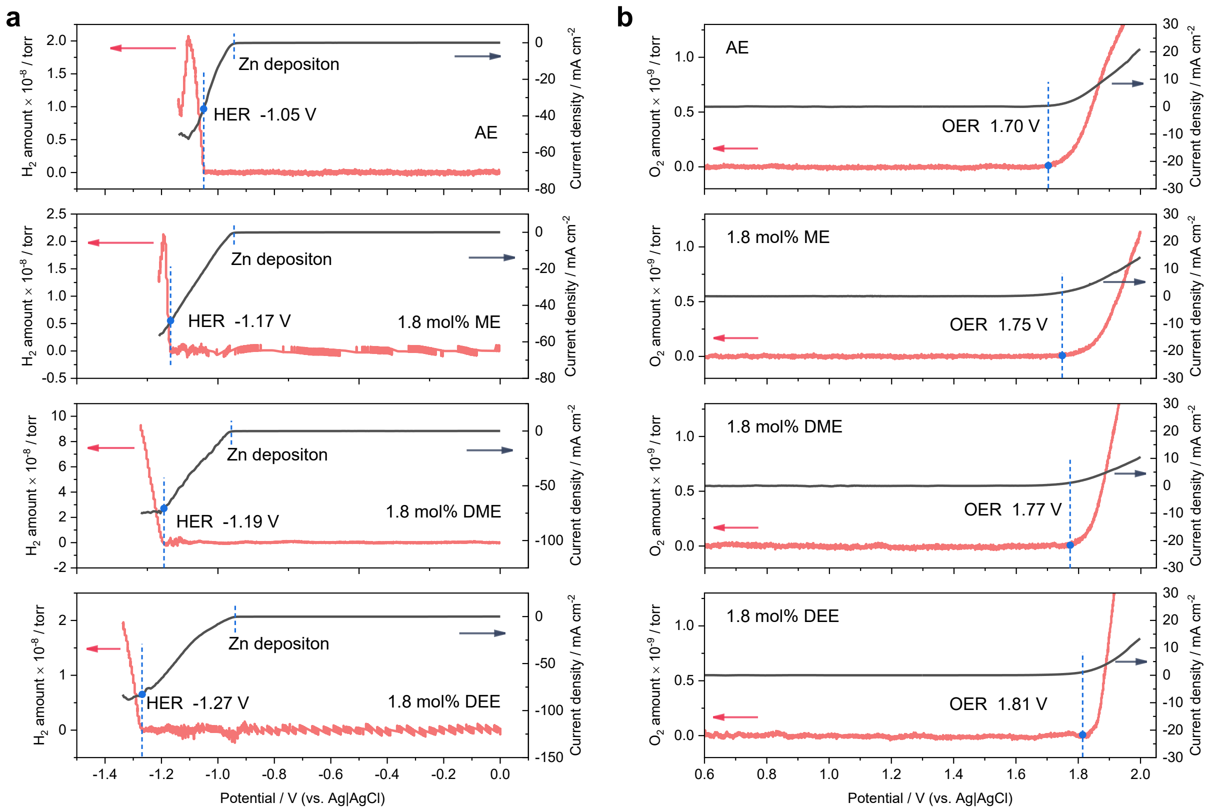

Supplementary Figure 30. Water splitting behaviour characterization by using DEMS measurement. (**a**) hydrogen evolution, (**b**) oxygen evolution, and corresponding LSV curves of Au electrode in AE, 1.8 mol% ME, 1.8 mol% DME and 1.8 mol% DEE electrolyte solutions.

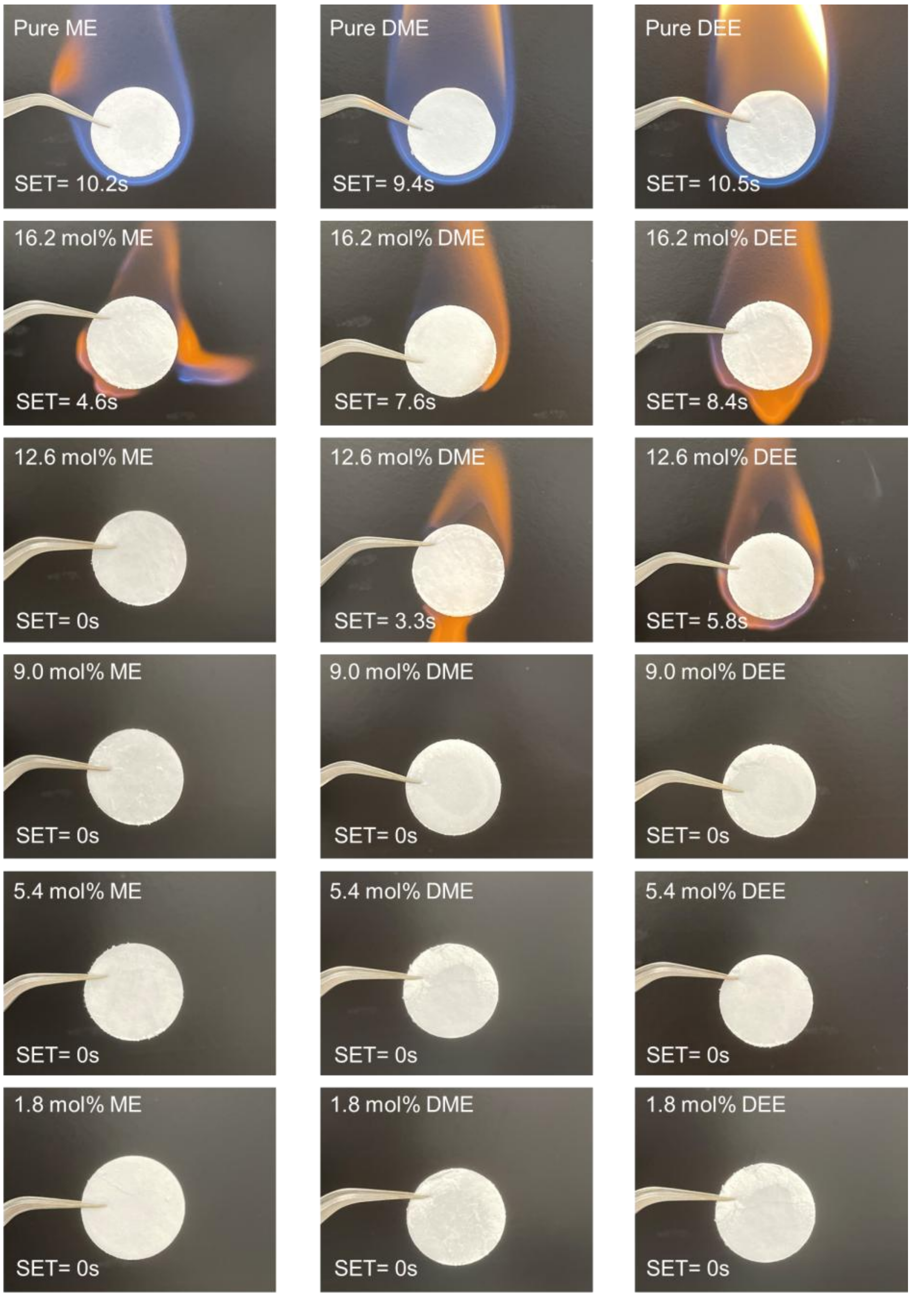

Supplementary Figure 31. Flammability test of 3 m $Zn(OTf)_2$ aqueous electrolyte solutions containing ME, DME and DEE organic additives. To carry out the test, 200 µL of electrolyte solution was absorbed into the porous structures of a glass fiber separator, and optical photos were captured after igniting the electrolyte for 3 seconds using a gas lighter. SET stands for the self-extinguishing time. It can be seen that the ME-containing electrolyte-soaked separator is non-flammable at concentrations below 12.6 mol%, while DME and DEE analogues are non-flammable below 9.0 mol%. However, the optimal concentrations for these organic additives vary: ME at 16.2 mol%, DME at 12.6 mol%, and DEE at 1.8 mol% (see also Fig. 5a in the main text). At their optimal concentrations, aqueous electrolyte solutions containing ME and DME additives become flammable. Only the DEE-containing electrolyte solutions remain non-flammable at their optimal concentrations, highlighting their safety.

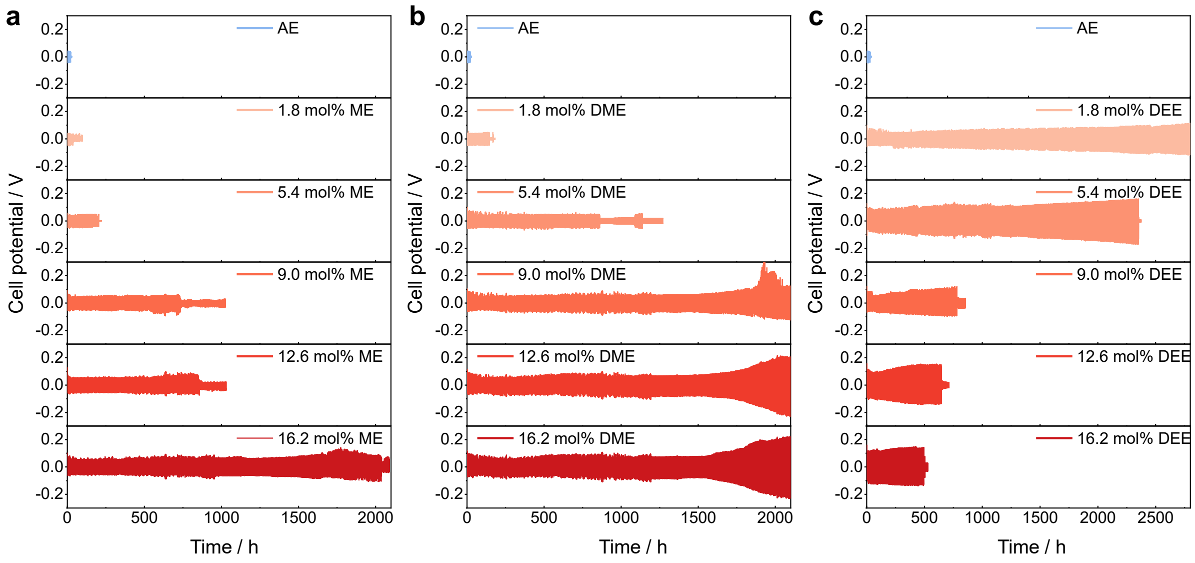

Supplementary Figure 32. Cycle performance of Zn||Zn coin cells at 5 mA cm^{-2} and 5 mAh cm^{-2} in aqueous electrolyte solutions containing (**a**) ME, (**b**) DME and (**c**) DEE organic additives in various concentrations.

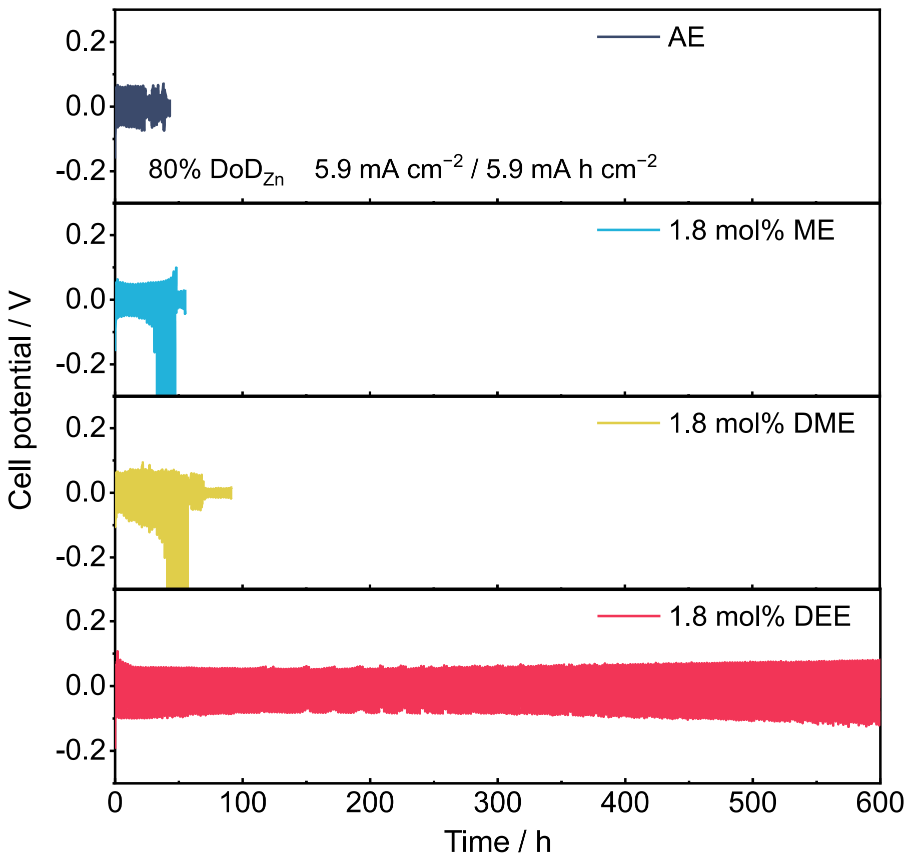

Supplementary Figure 33. Cycle performance of symmetric Zn||Zn coin cells with a high DoD_{Zn} of 80% in baseline AE, 1.8 mol% ME, 1.8 mol% DME and 1.8 mol% DEE electrolyte solutions. The applied current density is set to be 5.9 mA cm^{-2} with a total capacity of 5.9 mA h cm^{-2}.

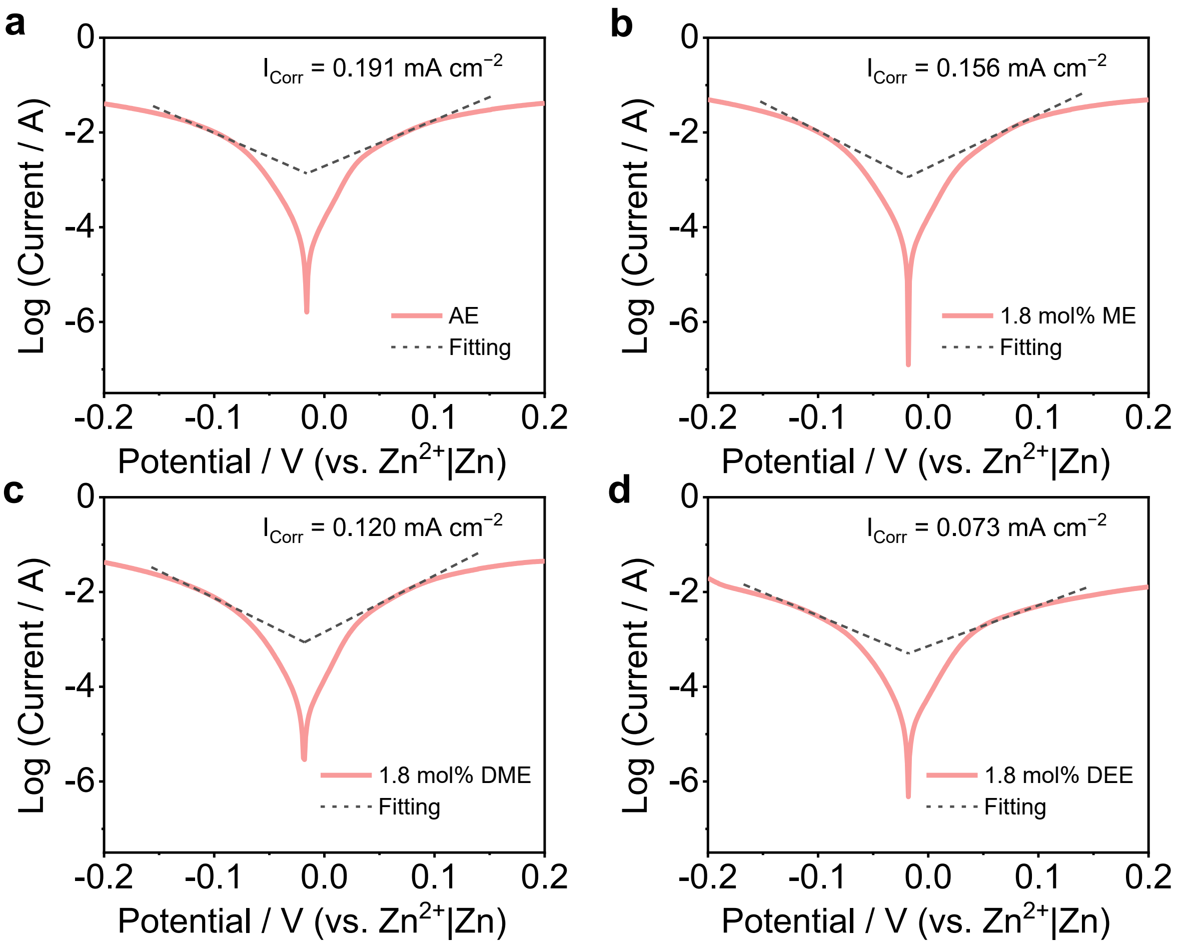

Supplementary Figure 34. Tafel plots of Zn electrodes tested in a Zn||Zn coin cell configuration using various electrolyte solutions: (**a**) baseline AE, (**b**) 1.8 mol% ME, (**c**) 1.8 mol% DME and (**d**) 1.8 mol% DEE.

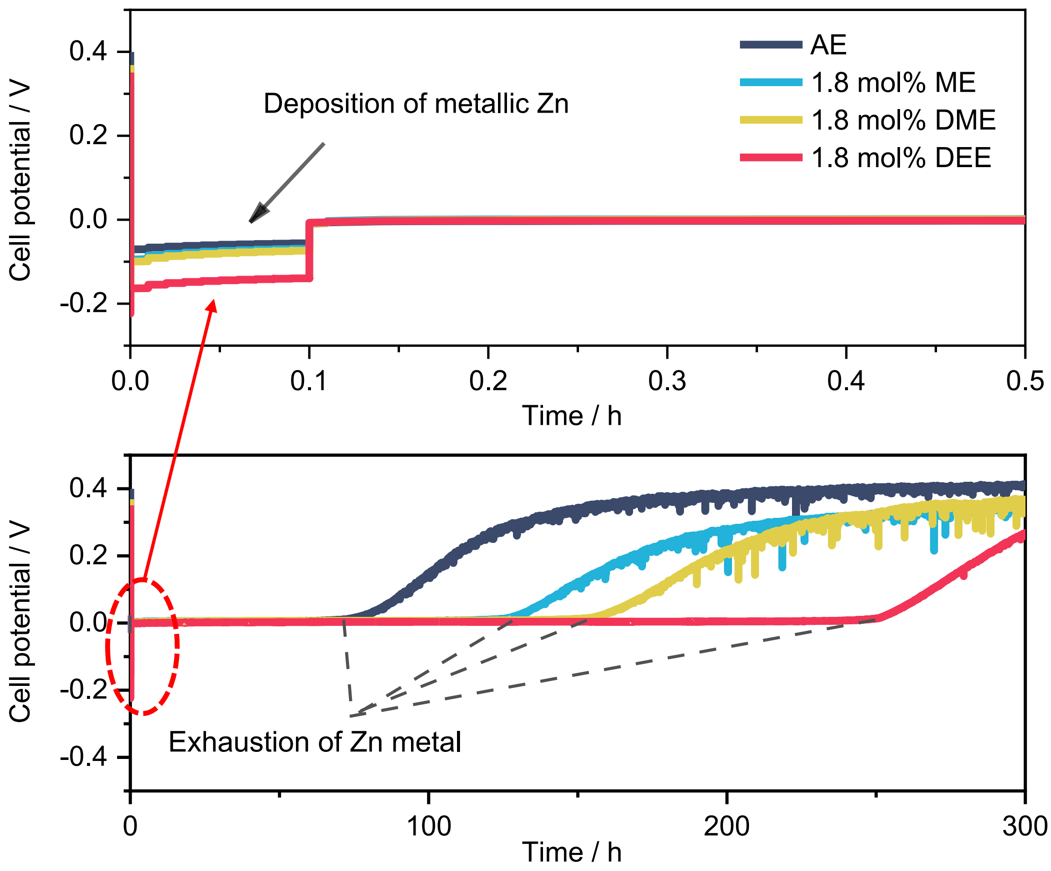

Supplementary Figure 35. Potential-time curves for Zn@Ti electrodes obtained by electroplating 0.565 mAh of metallic Zn on a Ti metal substrate in a Zn||Ti coin cell using the baseline AE, 1.8 mol% ME, 1.8 mol% DME and 1.8 mol% DEE electrolytes.

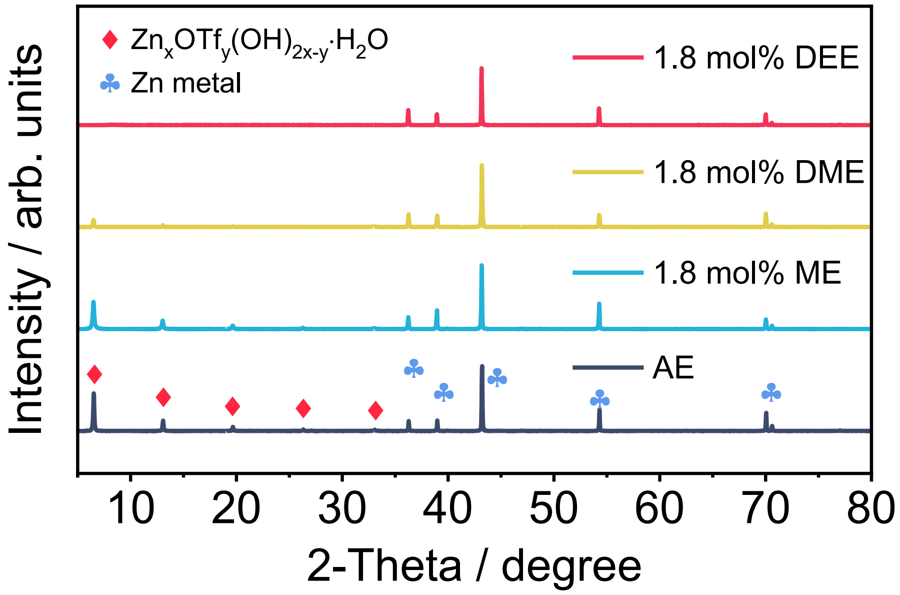

Supplementary Figure 36. Ex situ XRD measurements of Zn metal electrodes after immersion in baseline AE, 1.8 mol% ME, 1.8 mol% DME and 1.8 mol% DEE electrolyte solutions for 7 days at 25 °C in air environment. The immersion was conducted by placing a Zn foil in a vial containing 3 mL of electrolyte.

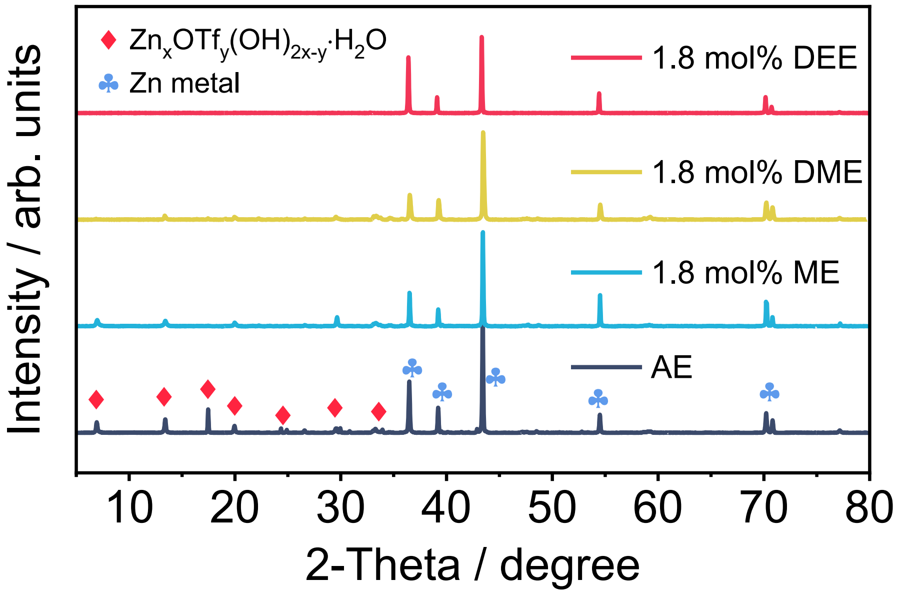

Supplementary Figure 37. Ex situ XRD measurements of Zn metal electrodes after 40 hours of cycles in a symmetric Zn||Zn coin cell configuration at 5 mA cm^{-2}, 5 mAh cm^{-2} and 25 °C using baseline AE, 1.8 mol% ME, 1.8 mol% DME and 1.8 mol% DEE electrolyte solutions.

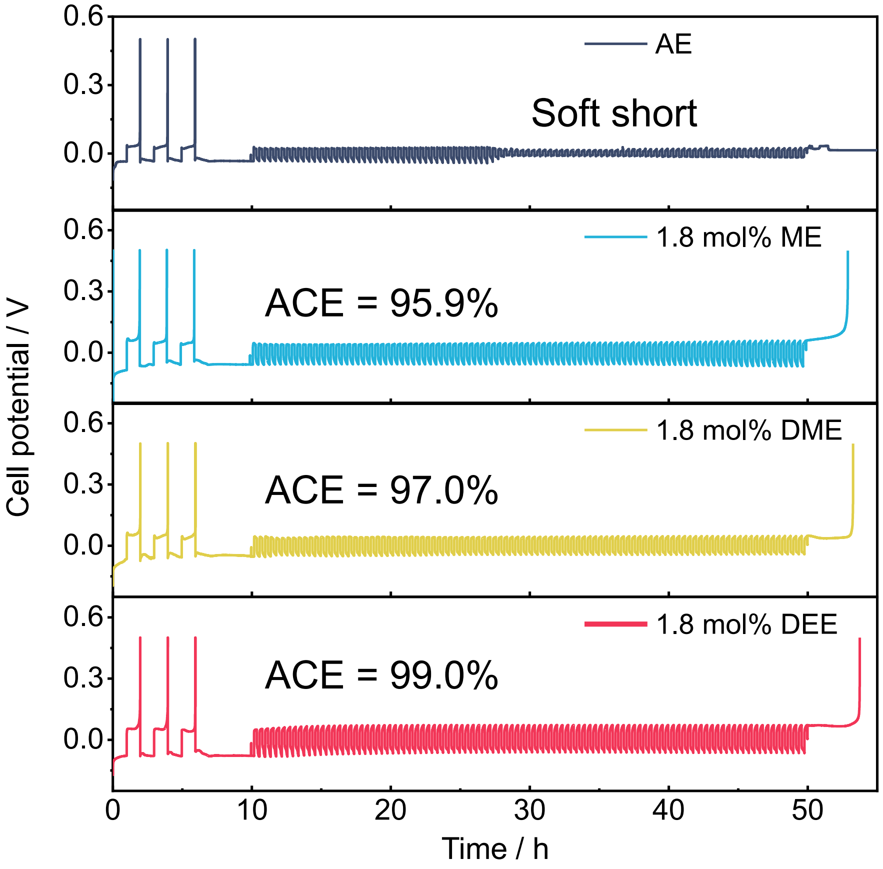

Supplementary Figure 38. Average CE of asymmetric Zn||Cu coin cells with baseline AE, 1.8 mol% ME, 1.8 mol% DME and 1.8 mol% DEE electrolyte solutions obtained by modified Aurbach's method [4] at 5 mA cm^{-2} and 25 °C . The CE were calculated using the equation of $CE = (nQ_c + Q_S)/(nQ_c + Q_r)$ (Eq. 2), where the Zn reservoir Q_r is set to be 20 mAh cm^{-2}, the capacity per cycle (Q_c) is 1 mAh cm^{-2}, Q_r is all the stripping Zn after charging to 0.5 V after the last cycle, and the overall cycle number (n) is 100.

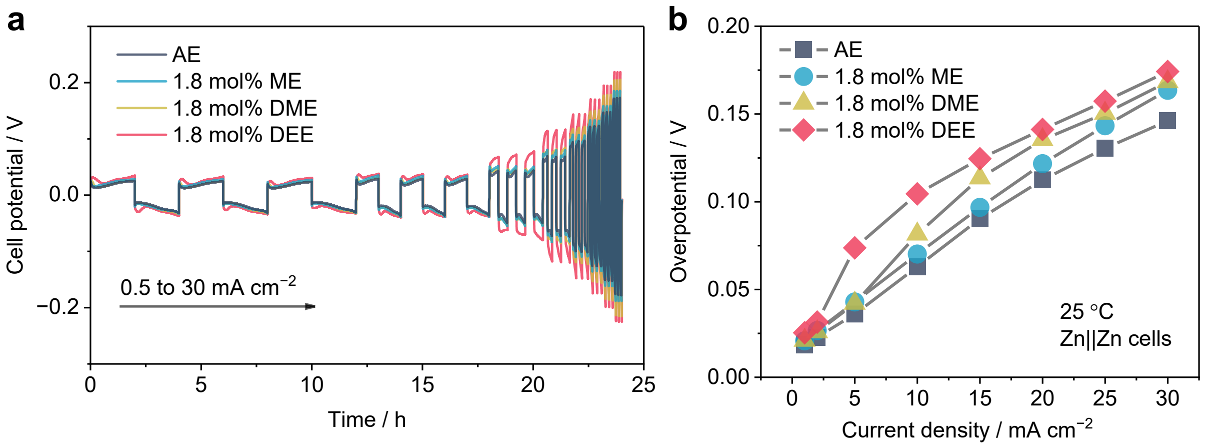

Supplementary Figure 39. Rate capability of Zn||Zn coin cells with baseline AE, 1.8 mol% ME, 1.8 mol% DME and 1.8 mol% DEE electrolyte solutions: (**a**) Time-potential profiles and (**b**) overpotentials. The overpotential is defined as the cell potential at 50% state of charge.

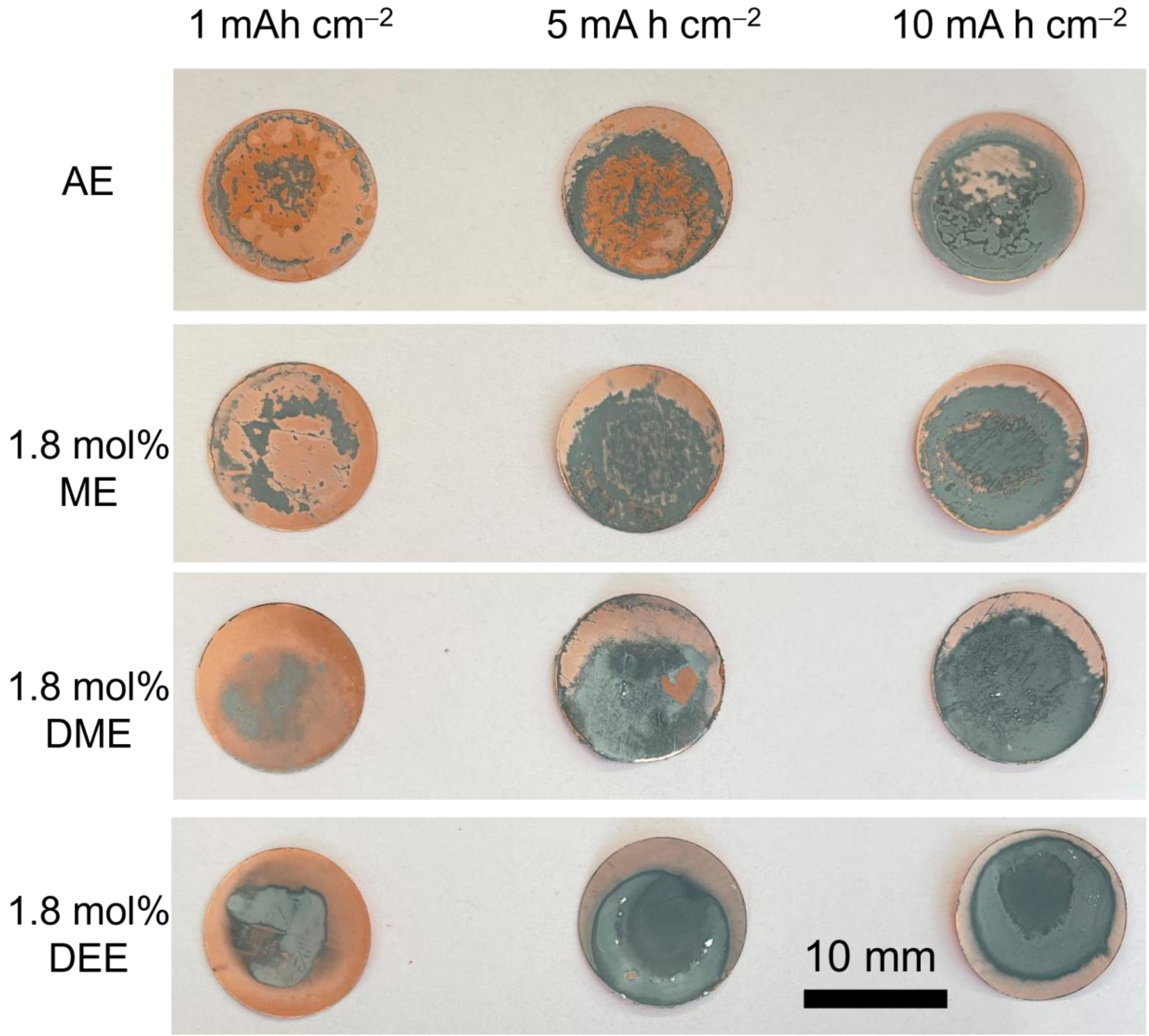

Supplementary Figure 40. Optical photos of electrodeposited Zn on Cu foils using the baseline AE, 1.8 mol% ME, 1.8 mol% DME and 1.8 mol% DEE electrolyte solution to qualitatively evaluate the morphology of the electrodeposited Zn. Dendrite formation is closely correlated with the distorted electric field distribution over the electrode induced by the tip-effect of the coarse metal surface [5,6]. The adsorption of the organic additives could provide an electrostatic shielding effect to eliminate the tip-effect and regulate Zn deposition without dendrite formation [7]. Due to strong adsorption of DEE-LEI, the 1.8 mol% DEE-containing electrolyte solution exhibits the most significant shielding effect, leading to more uniform Zn deposition on Cu substrate.

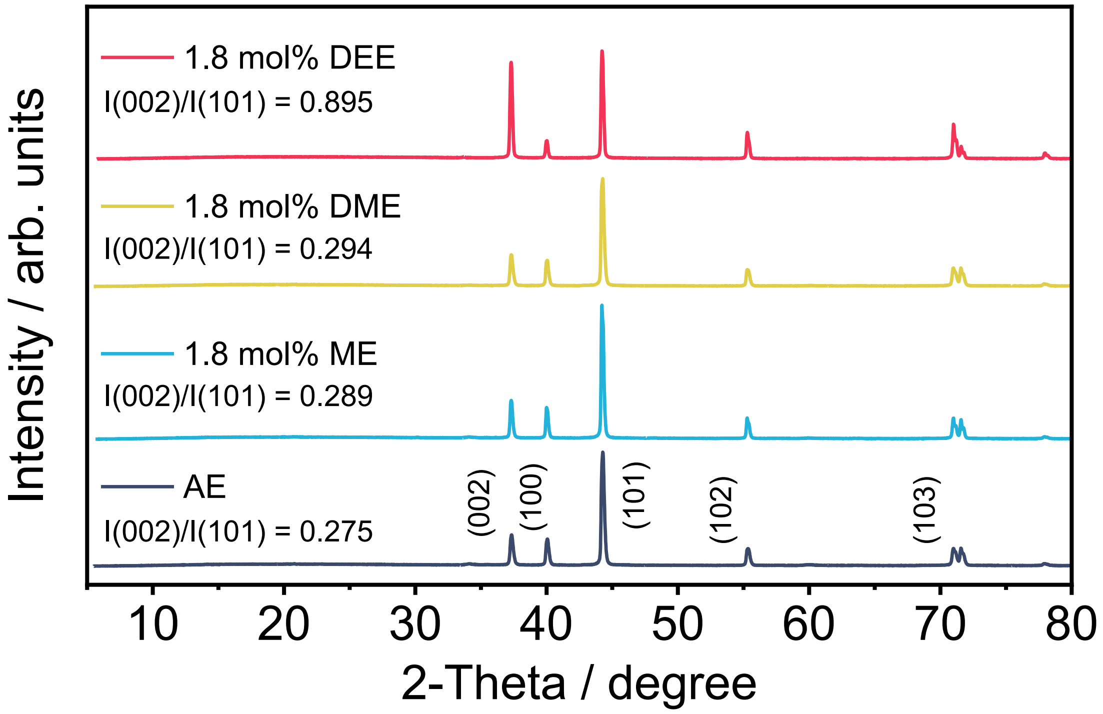

Supplementary Figure 41. Ex situ XRD measurements of Zn metal electrodes after electrodeposition in a Zn||Zn coin cell configuration at 5 mA cm^{-2} with a capacity of 20 mAh cm^{-2} using baseline AE, 1.8 mol% ME, 1.8 mol% DME and 1.8 mol% DEE electrolyte solutions.

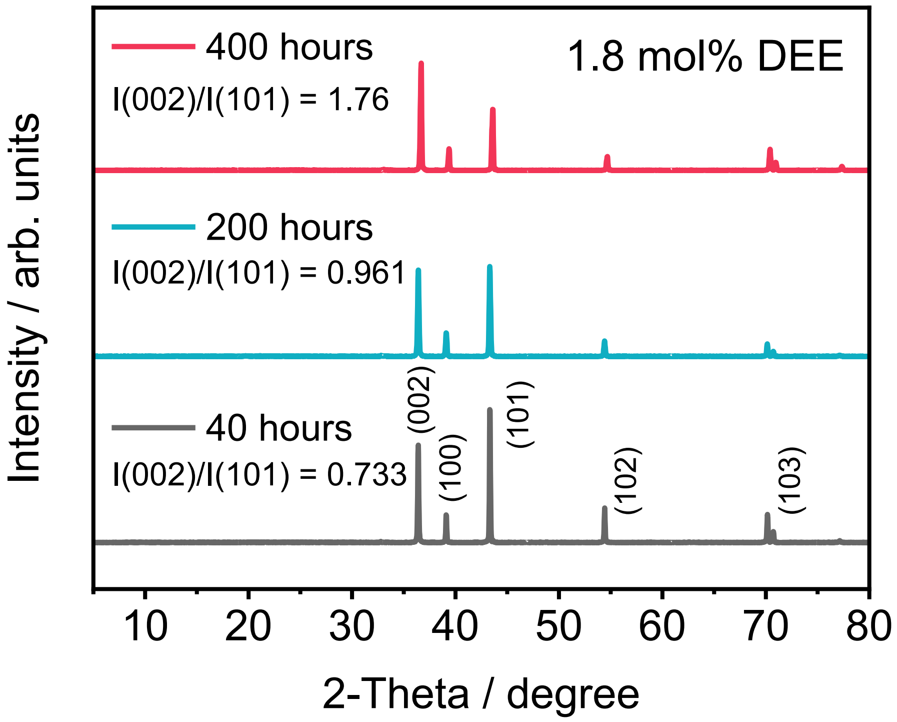

Supplementary Figure 42. Ex situ XRD measurements of Zn metal electrodes after various hours of cycling in a Zn||Zn coin cell configuration at 5 mA cm^{-2} and 5 mAh cm^{-2} using the 1.8 mol% DEE-containing electrolyte solution.

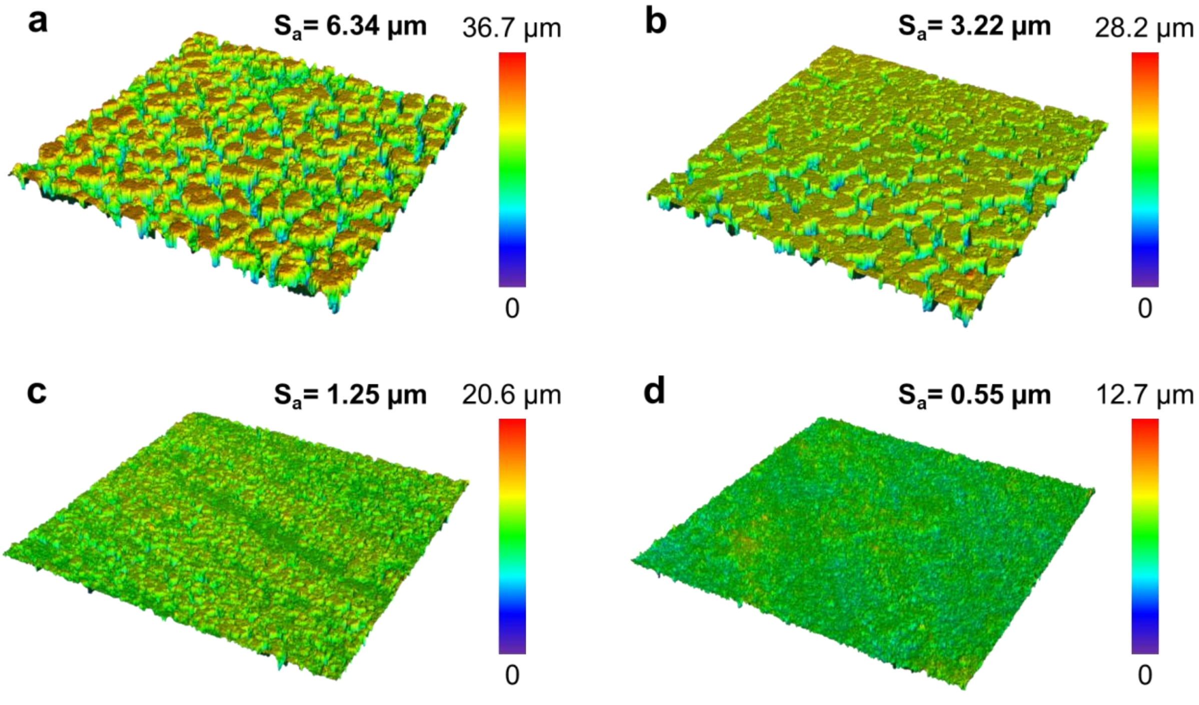

Supplementary Figure 43. Confocal laser microscopy images of Zn electrodes after electroplating at 5 mA cm^{-2} with a capacity of 20 mA h cm^{-2} in various electrolyte solutions: (**a**) baseline AE, (**b**) 1.8 mol% ME, (**c**) 1.8 mol% DME and (**d**) 1.8 mol% DEE. S_a = arithmetic mean height.

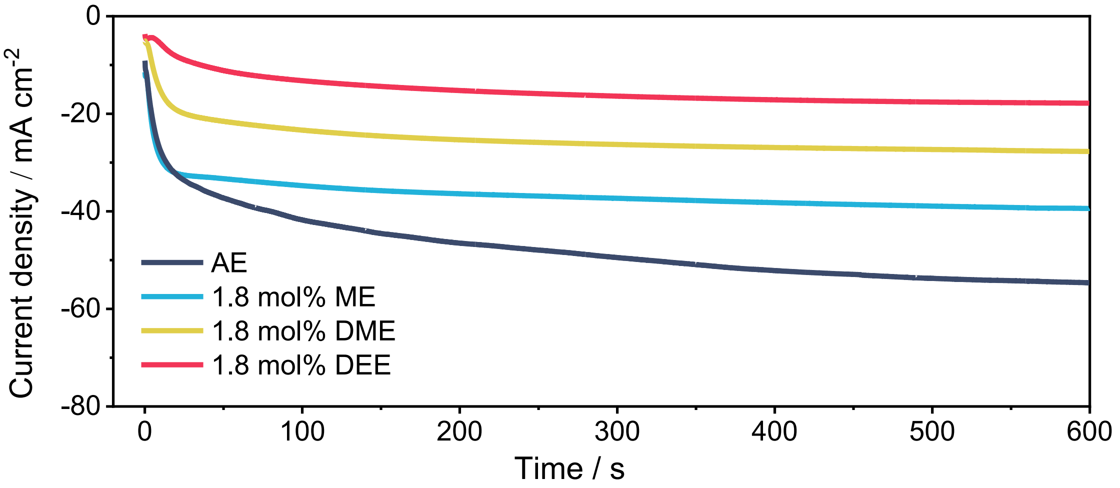

Supplementary Figure 44. Chronoamperogram test of symmetric Zn||Zn coin cells with baseline AE, 1.8 mol% ME, 1.8 mol% DME and 1.8 mol% DEE electrolyte solutions at a constant potential of −150 mV.

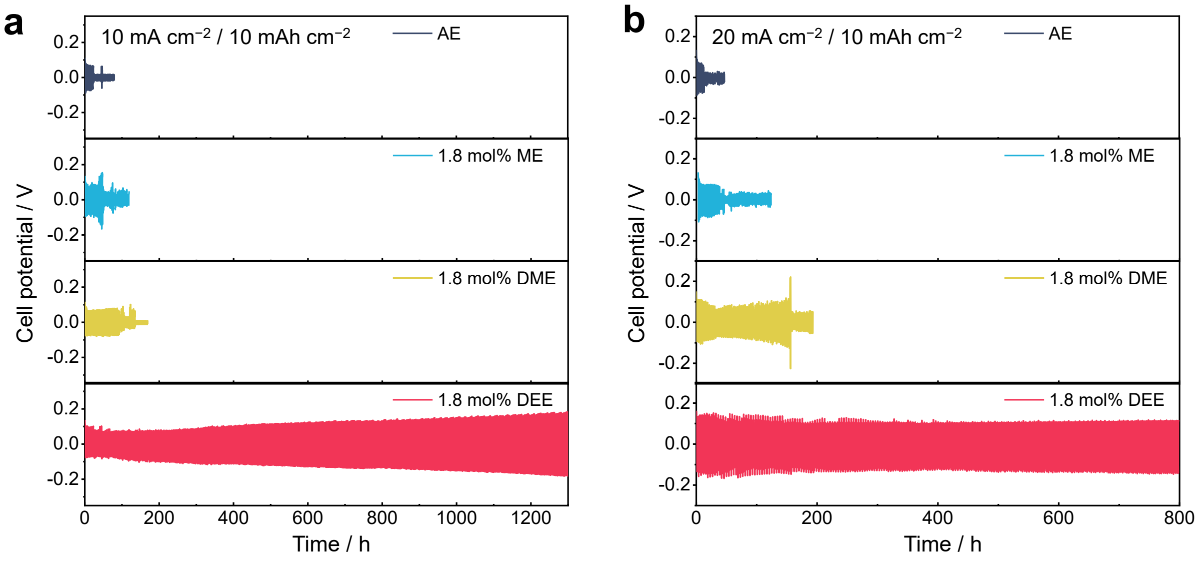

Supplementary Figure 45. Cycling performance of symmetric Zn||Zn coin cells at (**a**) 10 mA cm^{-2} and 10 mAh cm^{-2} and (**b**) 20 mA cm^{-2} and 10 mAh cm^{-2} in baseline AE, 1.8 mol% ME, 1.8 mol% DME and 1.8 mol% DEE electrolyte solutions.

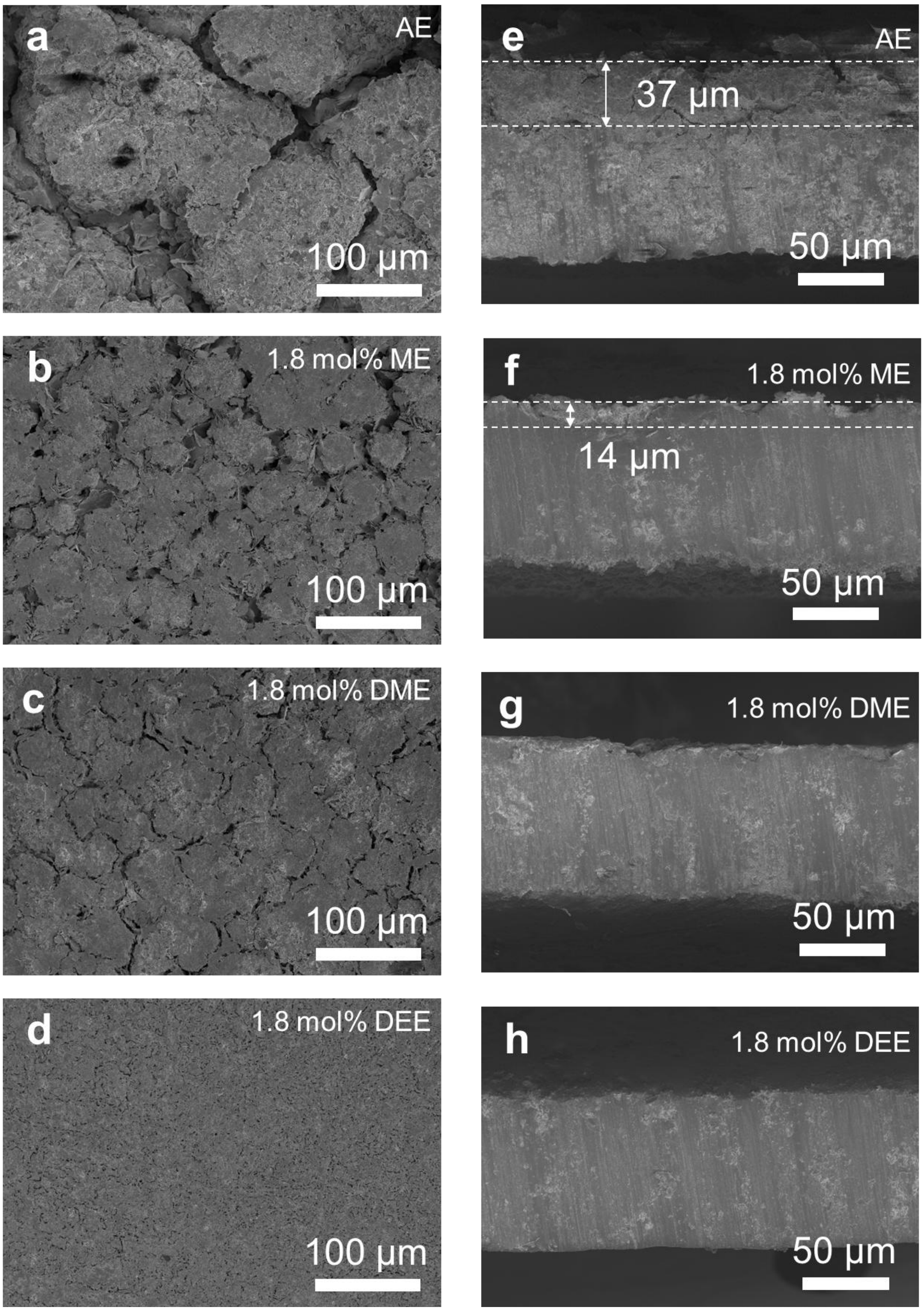

Supplementary Figure 46. (**a** to **d**) Top and (**e** to **h**) cross-section ex situ SEM images of Zn metals after cycling at 5 mA cm^{-2} and 5 mA h cm^{-2} in a Zn||Zn coin cell configuration for 40 hours with baseline AE, 1.8 mol% ME, 1.8 mol% DME and 1.8 mol% DEE electrolyte solutions.

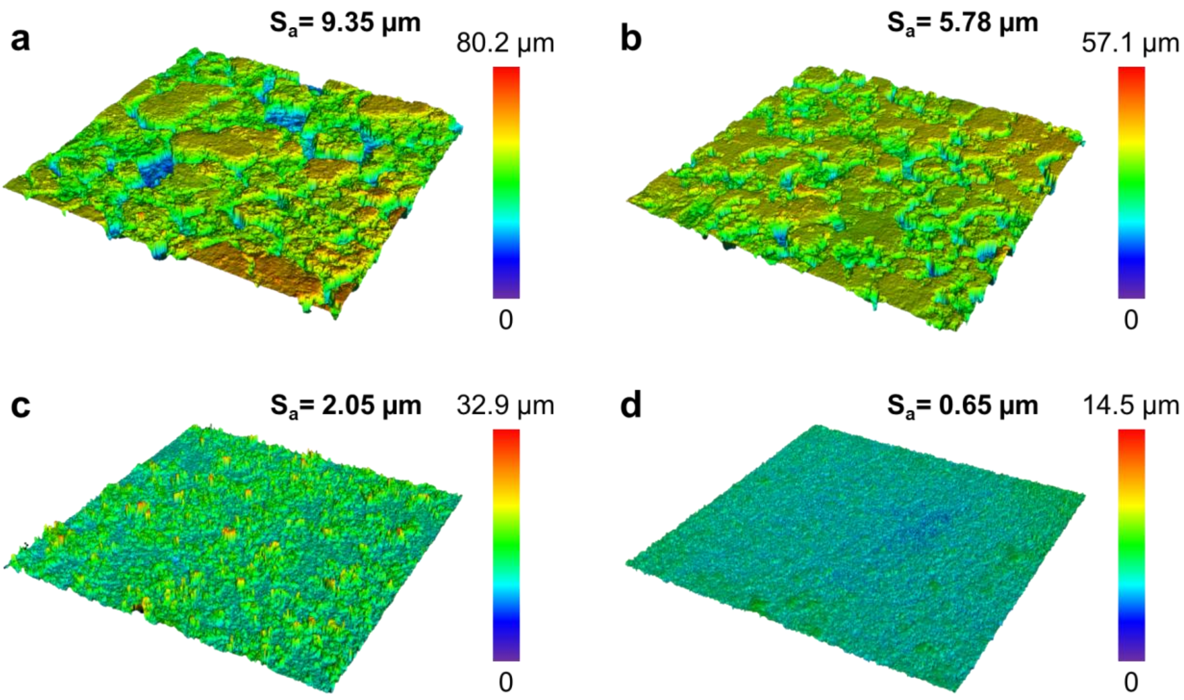

Supplementary Figure 47. Confocal laser microscopy images of Zn metals after cycling at 5 mA cm^{-2} and 5 mA h cm^{-2} in a Zn||Zn coin cell configuration for 40 hours with various electrolyte solutions: (**a**) baseline AE, (**b**) 1.8 mol% ME, (**c**) 1.8 mol% DME and (**d**) 1.8 mol% DEE. S_a = arithmetic mean height.

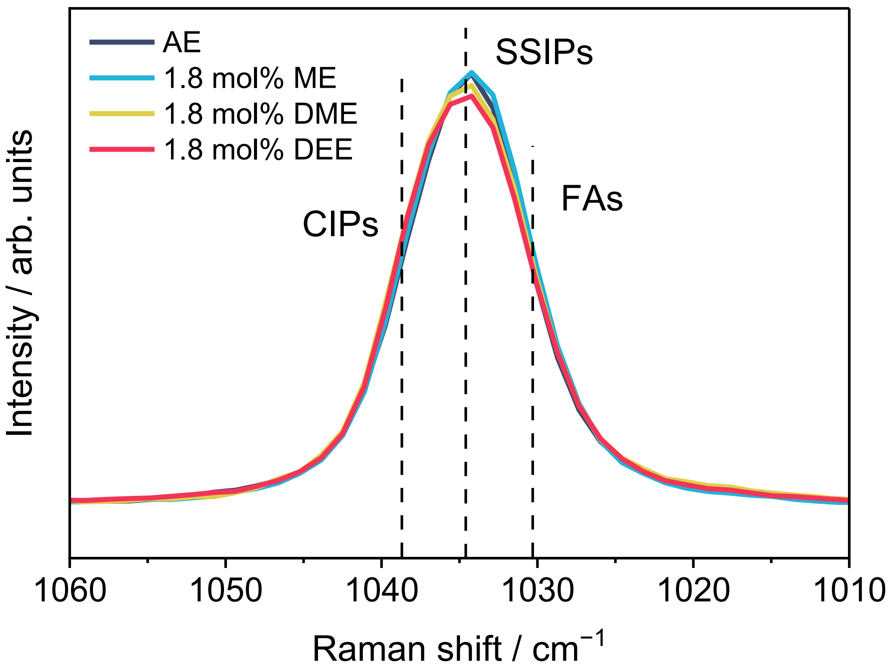

Supplementary Figure 48. Raman spectra of baseline AE, 1.8 mol% ME, 1.8 mol% DME and 1.8 mol% DEE electrolyte solutions, which indicates the $v_s(SO_3)$ shifting of the OTf^- anion. Contact ion pairs: CIPs, aggregate cation-anion pairs: AGGs, Free anions: FAs.

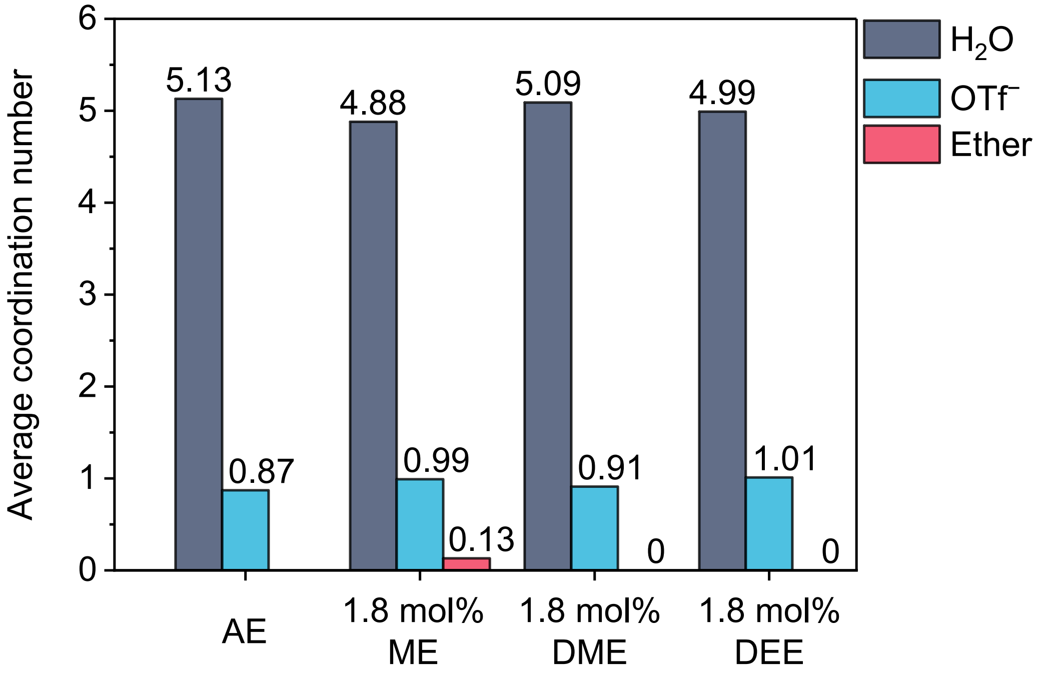

Supplementary Figure 49. Average coordination number of water, OTf^- anion and ethers (ME, DME, and DEE) in the Zn^{2+} solvation sheath, obtained from MD simulations.

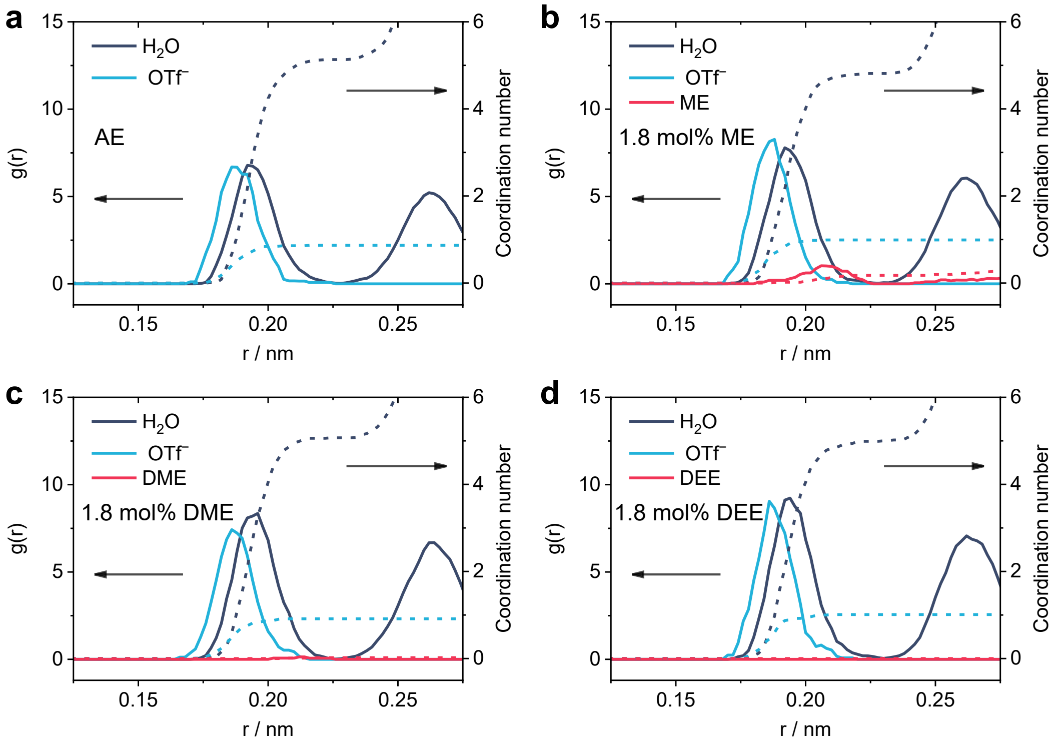

Supplementary Figure 50. Radial distribution function (RDF) of Zn-O (H_2O), Zn-O (OTf^-) and Zn-O (ether) in (**a**) baseline AE, (**b**) 1.8 mol% ME, (**c**) 1.8 mol% DME, (**d**) 1.8 mol% DEE electrolyte solutions.

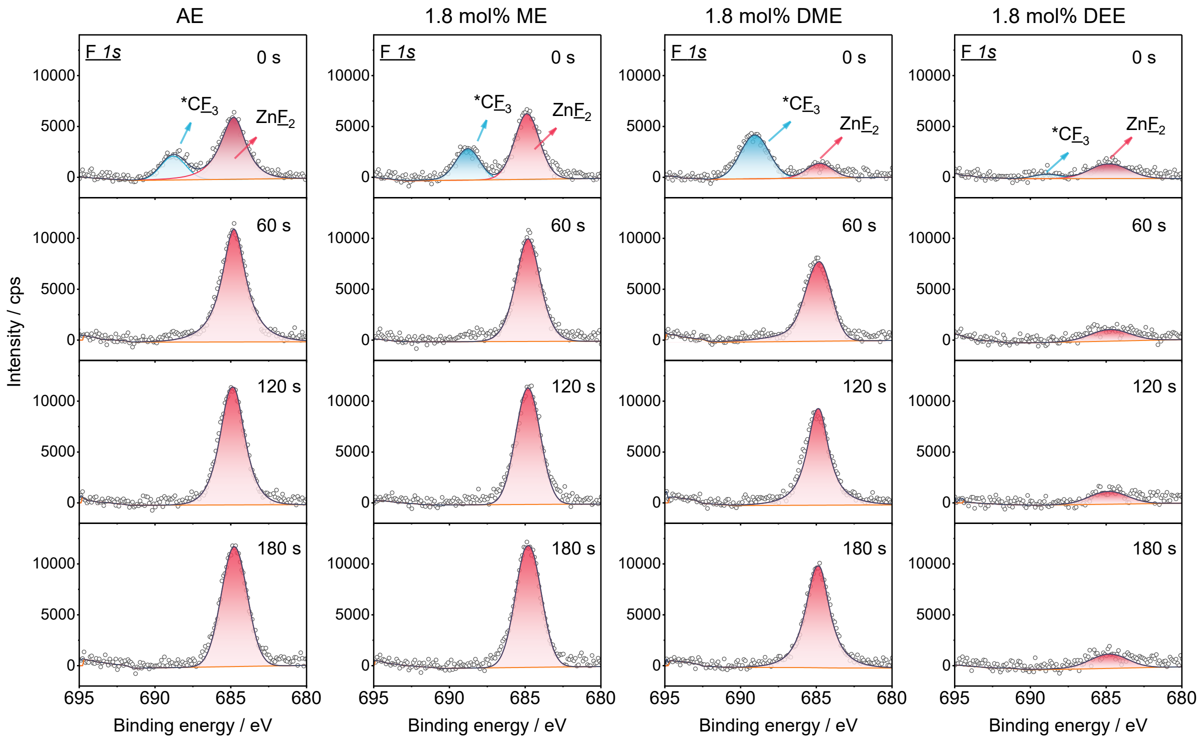

Supplementary Figure 51. Ex situ F *1s* XPS spectra of Zn metal electrodes after 10 cycles at 1 mA cm^{-2} and 1 mA h cm^{-2} in a Zn||Zn coin cell configuration with baseline AE, 1.8 mol% ME, 1.8 mol% DME and 1.8 mol% DEE electrolyte solutions.

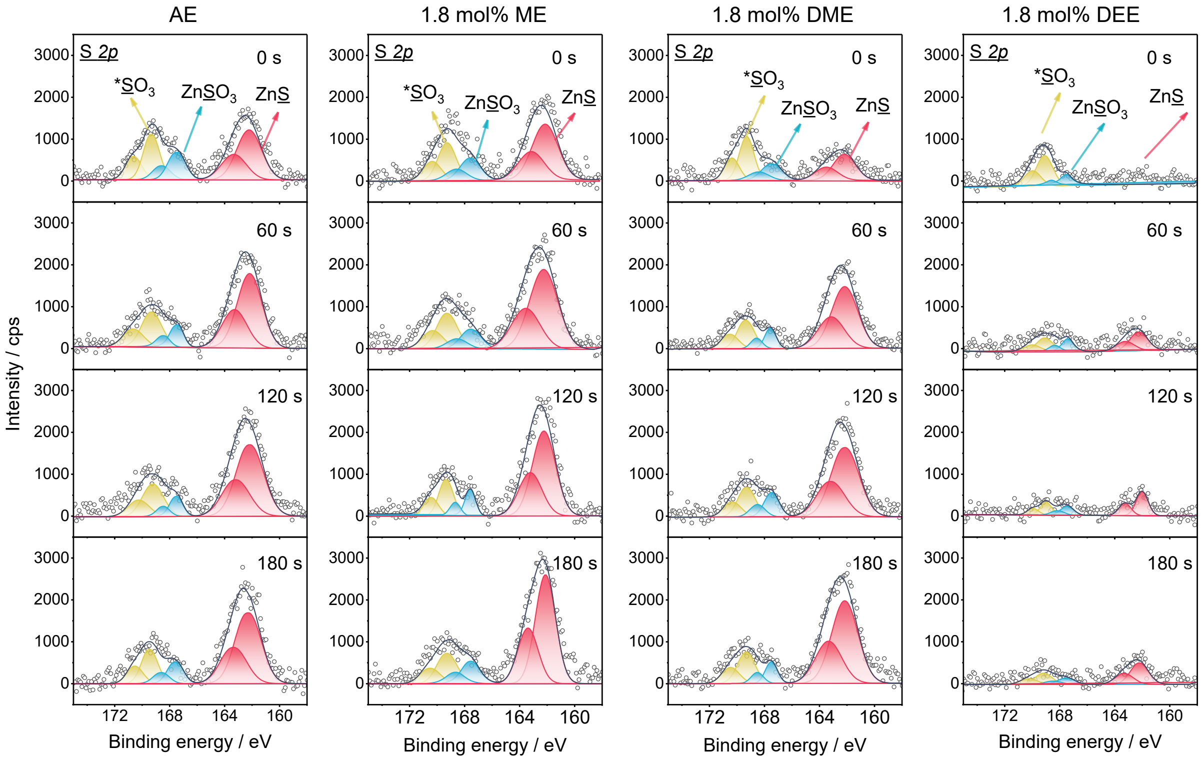

Supplementary Figure 52. Ex situ S *2p* XPS spectra of Zn metal electrodes after 10 cycles at 1 mA cm^{-2} and 1 mA h cm^{-2} in a Zn||Zn coin cell configuration with baseline AE, 1.8 mol% ME, 1.8 mol% DME and 1.8 mol% DEE electrolyte solutions.

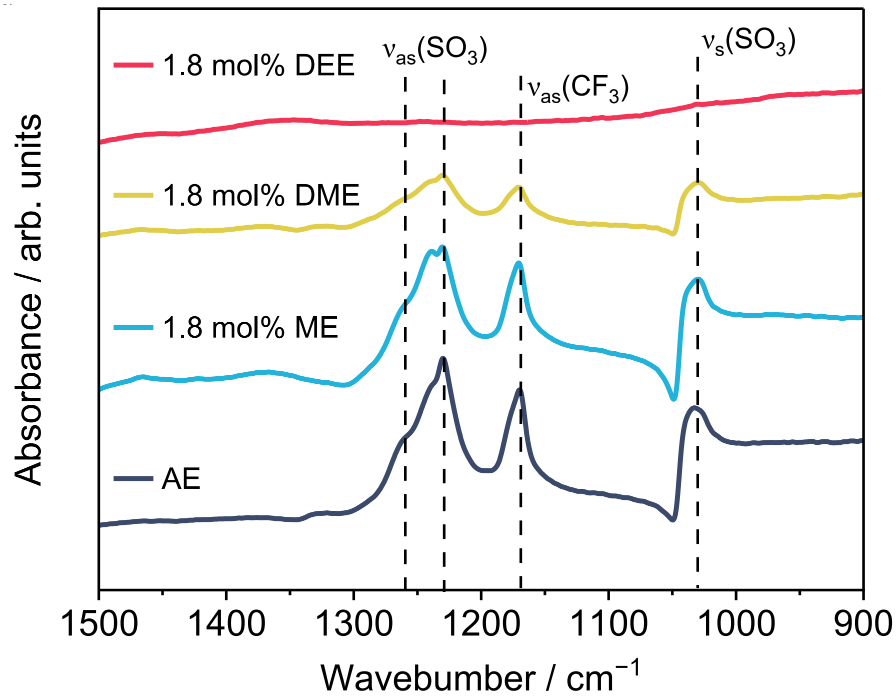

Supplementary Figure 53. Ex situ FTIR spectra of Zn metal electrodes after 10 cycles at 1 mA cm^{-2} and 1 mA h cm^{-2} in a Zn||Zn coin cell configuration with baseline AE, 1.8 mol% ME, 1.8 mol% DME and 1.8 mol% DEE electrolyte solutions, showing characteristic signals ($\nu_{as}(SO_3)$, $\nu_{as}(CF_3)$, and $\nu_s(SO_3)$) from decomposition products of the OTf$^-$ anion.

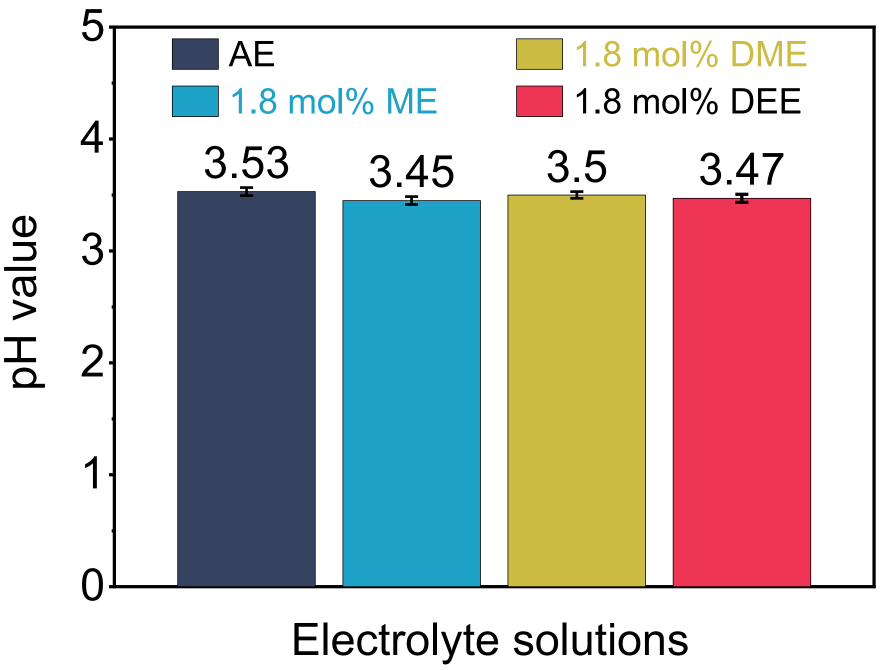

Supplementary Figure 54. pH values of baseline AE, 1.8 mol% ME, 1.8 mol% DME and 1.8 mol% DEE electrolyte solutions. The error bar represents the standard deviation based on three independent measurements.

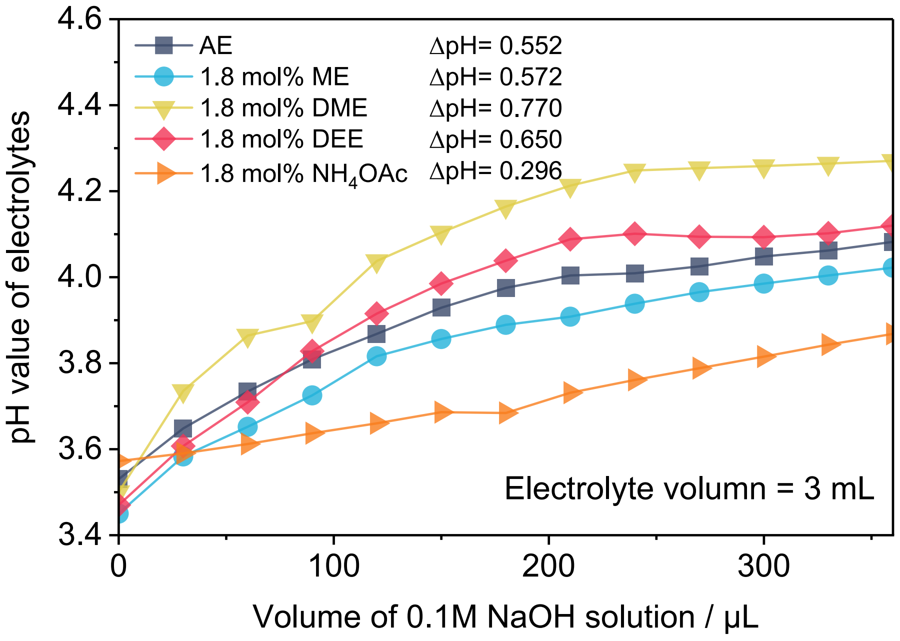

Supplementary Figure 55. Titration test of baseline AE, 1.8 mol% ME, 1.8 mol% DME and 1.8 mol% DEE electrolyte solutions, with NH_4OAc (a pH-buffering additive) used as a benchmark. The ΔpH was defined as the difference between the final and initial pH values measured during the titration.

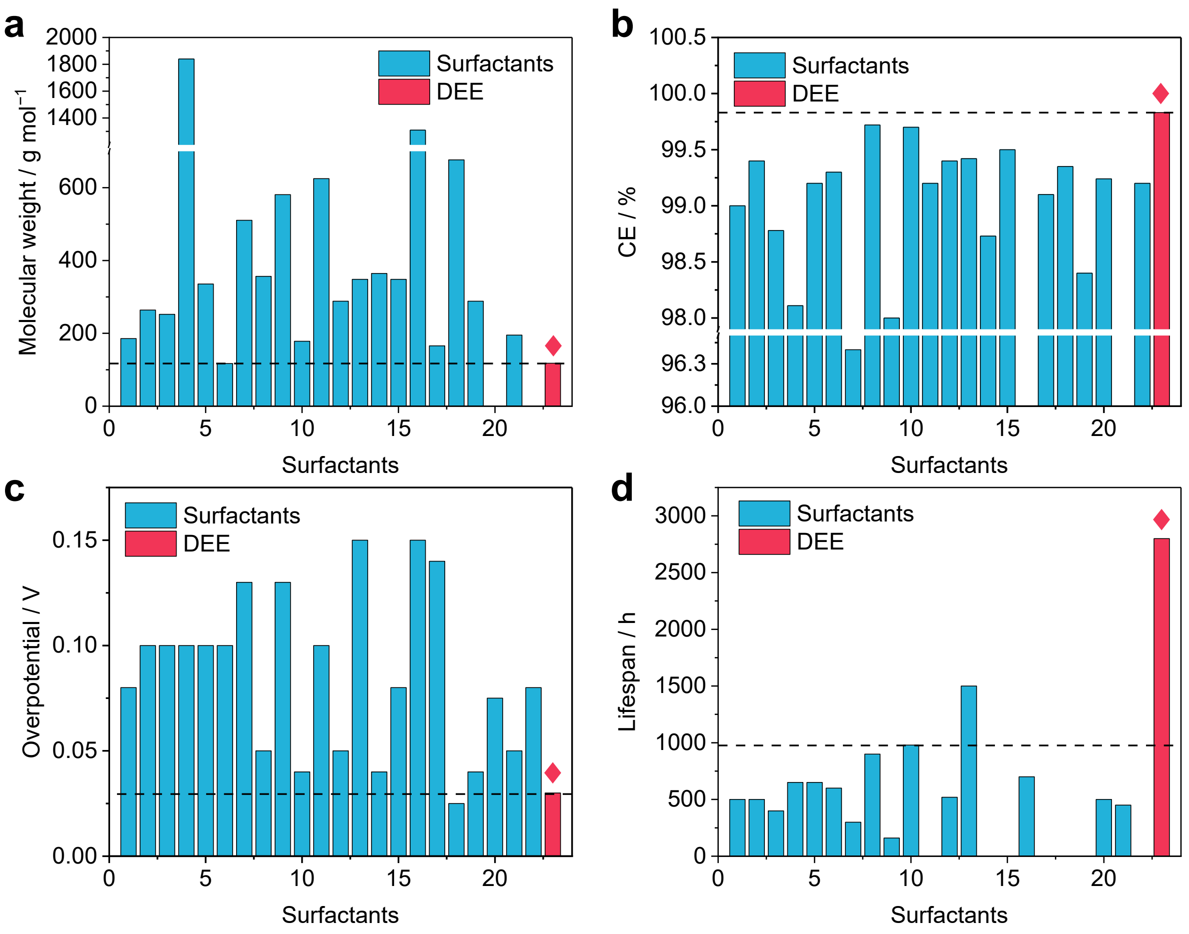

Supplementary Figure 56. Comparison of (**a**) molecular weight, (**b**) Coulombic efficiency, (**c**) overpotential and (**d**) lifespan of Zn metal electrodes tested in a Zn||Zn coin cell configuration with DEE-based LEI-generating electrolyte solution with previously reported electrolytes modified by surfactants. The detailed information of surfactants can be found in Supplementary Table 4 and Supplementary Note 3.

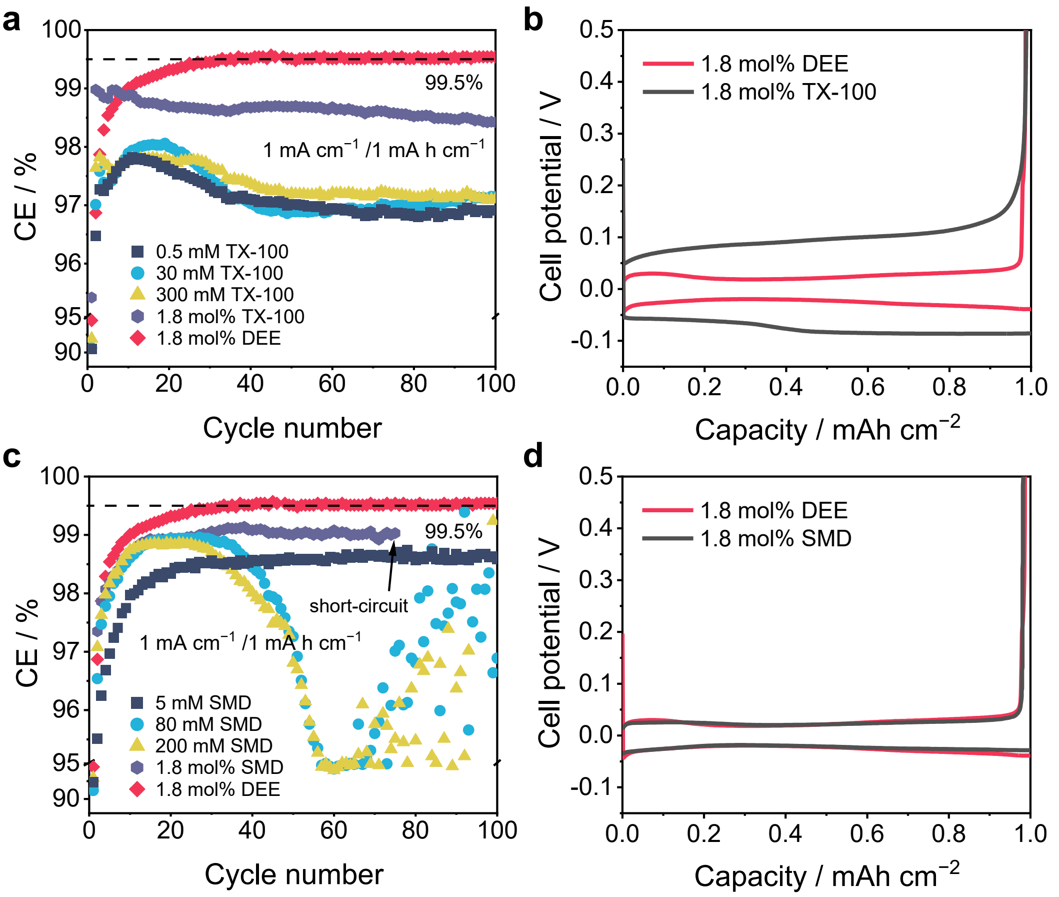

Supplementary Figure 57. Coulombic efficiency and selective capacity-potential profiles of asymmetric Zn||Cu cells using electrolytes containing (**a** and **b**) TX-100 and (**c** and **d**) SMD surfactants at 1 mA cm^{-2} and 1 mA h cm^{-2}. TX-100: Triton X-100, SMD: Sodium methylenedinaphthalene disulphonate. The surfactant concentrations are presented in mM to remain consistent with the format used in the referenced study. The concentration of 1.8 mol% corresponds approximately to 480 mM for TX-100 and 470 mM for SMD.

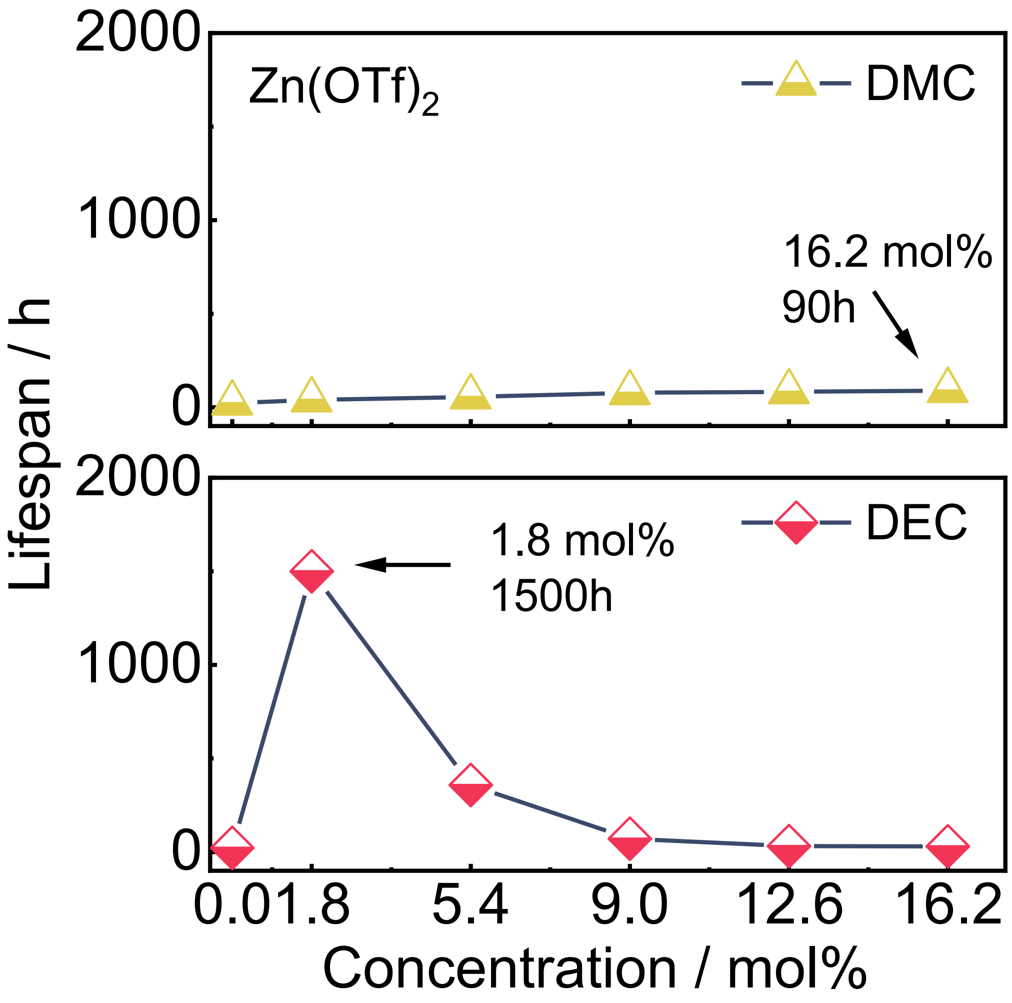

Supplementary Figure 58. Correlation between the lifespan of symmetric Zn||Zn coin cells and concentrations of DMC and DEC organic additives in the 3 m $Zn(OTf)_2$ aqueous electrolyte solution.

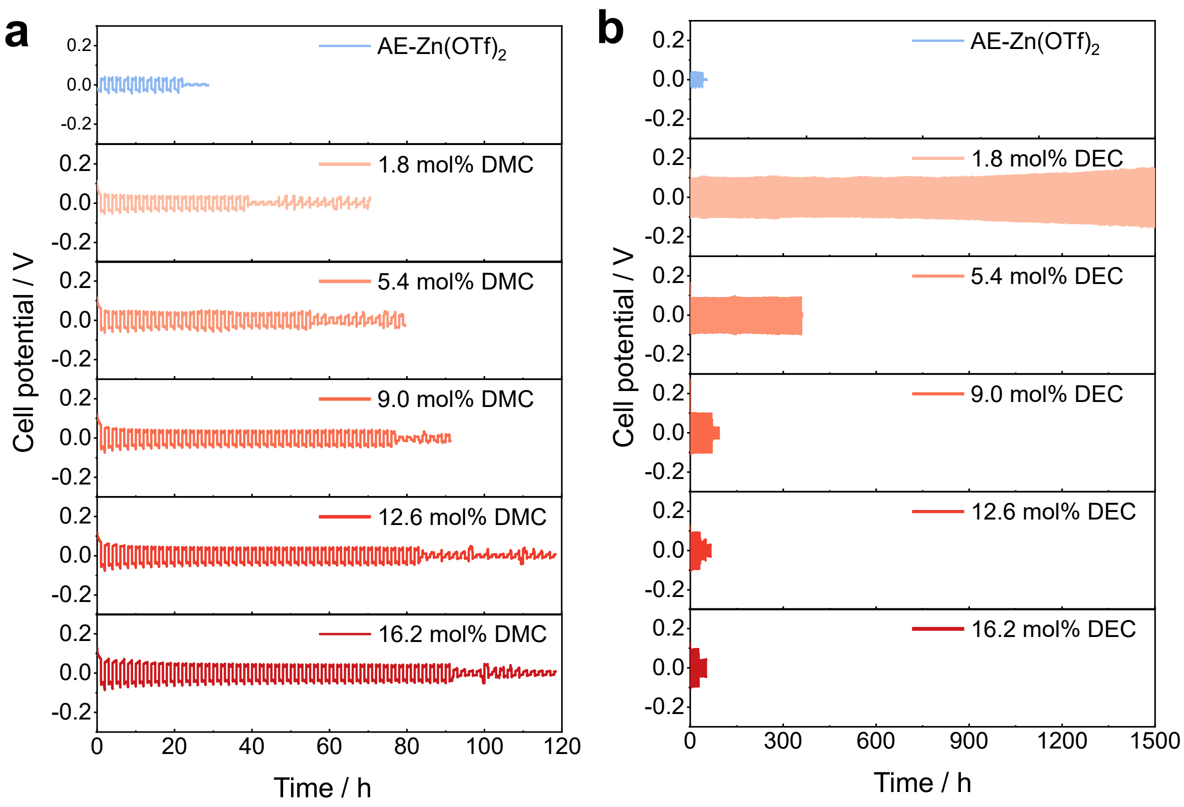

Supplementary Figure 59. Cycle performance of symmetric Zn||Zn coin cells at 5 mA cm^{-2} and 5 mA h cm^{-2} in 3m $Zn(OTf)_2$ electrolyte solutions containing (**a**) DMC and (**b**) DEC organic additives in various concentrations.

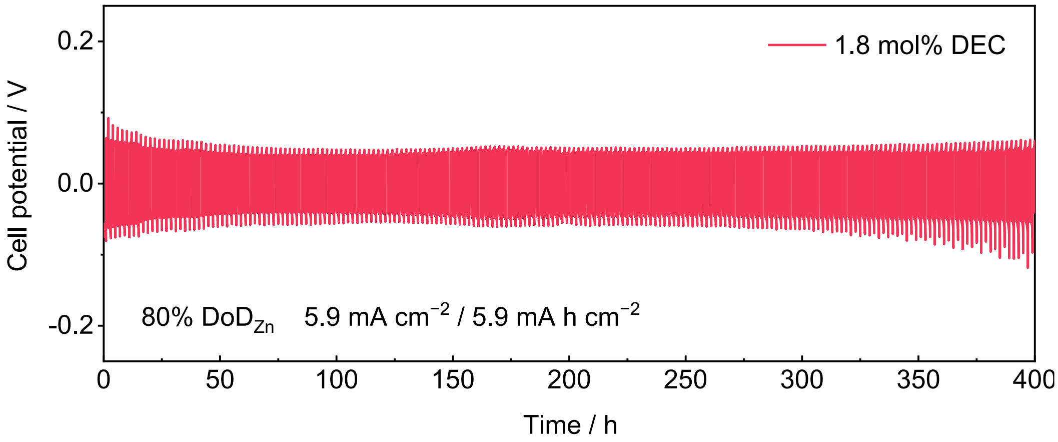

Supplementary Figure 60. Cycle performance of symmetric Zn||Zn coin cells with a high DoD$_{Zn}$ of 80% in 1.8 mol% DEC-containing electrolyte solution. The applied current density is 5.9 mA cm^{-2} with a total capacity of 5.9 mA h cm^{-2}.

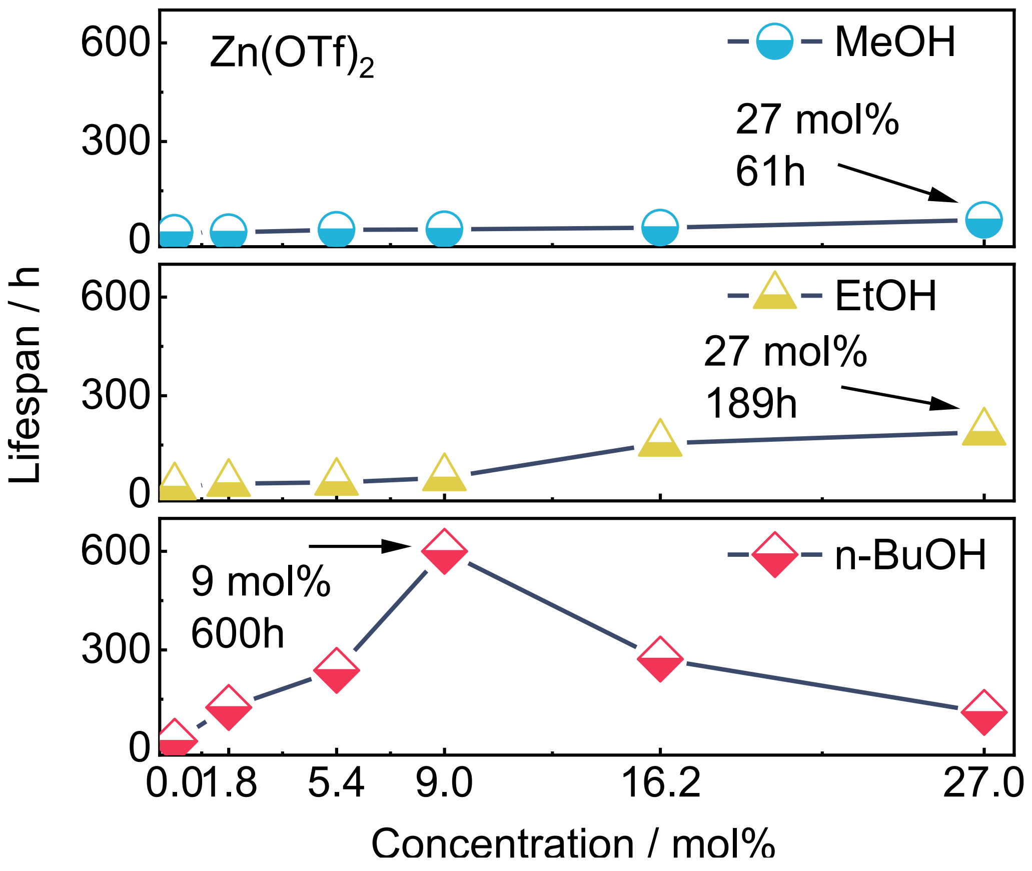

Supplementary Figure 61. Correlation between cycling performance of symmetric Zn||Zn coin cells and concentrations of MeOH, EtOH and n-BuOH organic additives in the 3 m $Zn(OTf)_2$ aqueous electrolyte solution.

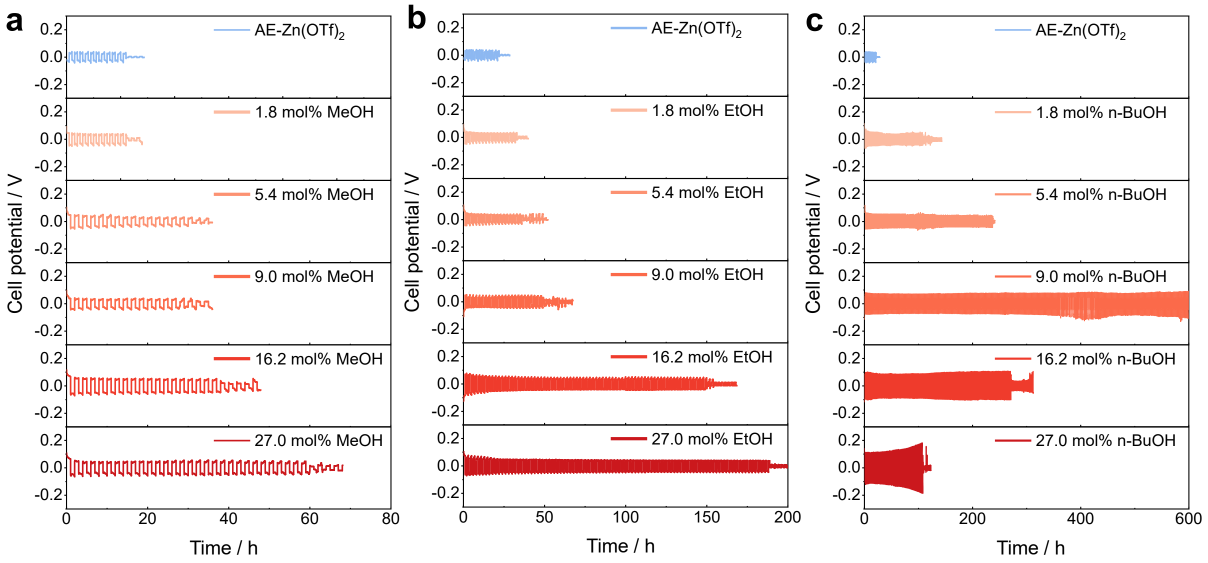

Supplementary Figure 62. Cycle performance of symmetric Zn||Zn cells at 5 mA cm^{-2} and 5 mA h cm^{-2} in the 3 m Zn(OTf)$_2$ aqueous electrolyte solutions containing various organic additives in different concentrations: (**a**) MeOH, (**b**) EtOH and (**c**) n-BuOH.

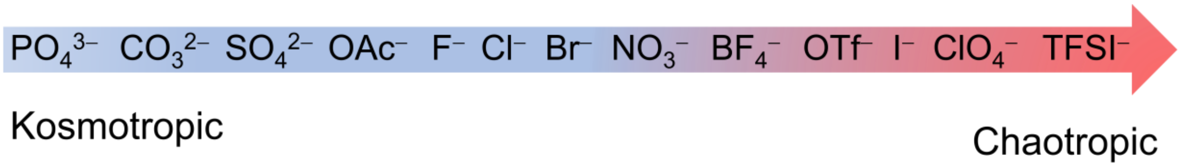

Supplementary Figure 63. Hofmeister series of representative anions, illustrating their classification from kosmotropic (salting-out) to chaotropic (salting-in) behaviour. Kosmotropic anions (e.g., SO_4^{2-}) strongly interact with water molecules, enhancing water structure and promoting the exclusion of organic species (salting-out effect), whereas chaotropic anions (e.g., ClO_4^-) exhibit weaker hydration and disrupt the water structure, thereby increasing the solubility of organic species (salting-in effect). Most anions fall between these two extremes and exhibit intermediate behaviour.

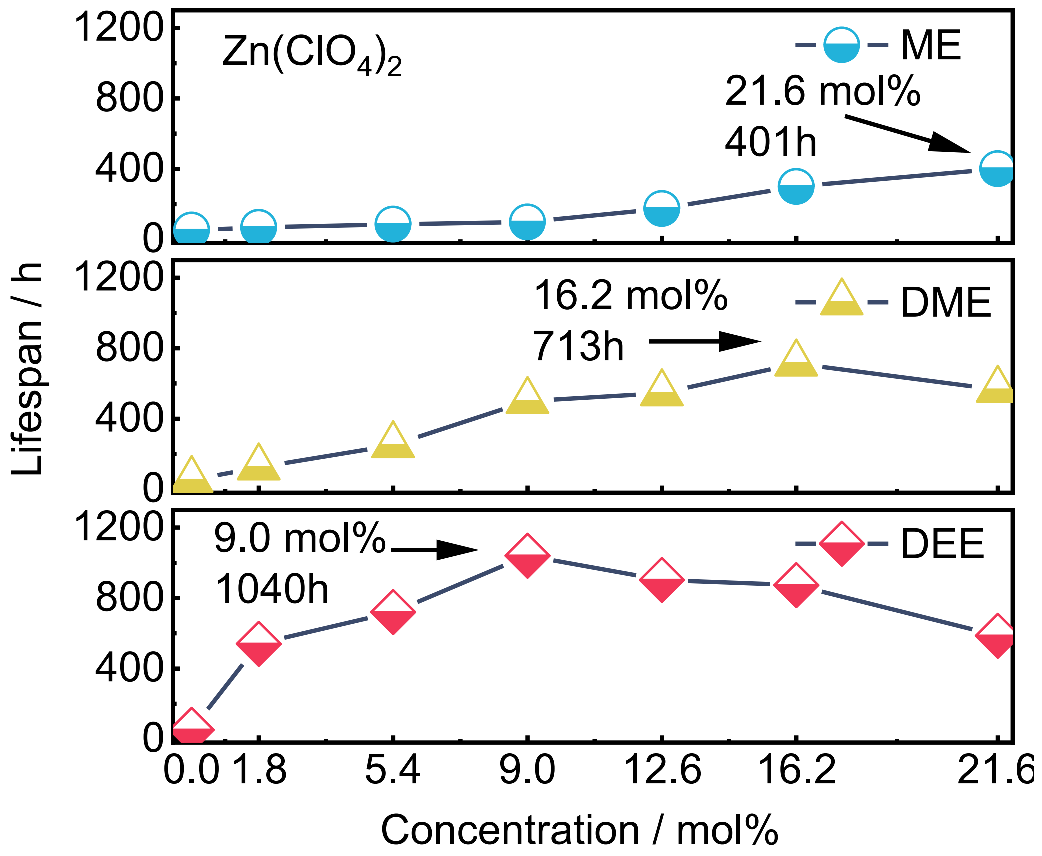

Supplementary Figure 64. Correlation between the cycling performance of symmetric Zn||Zn coin cells and concentrations of ME, DME and DME organic additives in aqueous 3 m $Zn(ClO_4)_2$ electrolyte solutions.

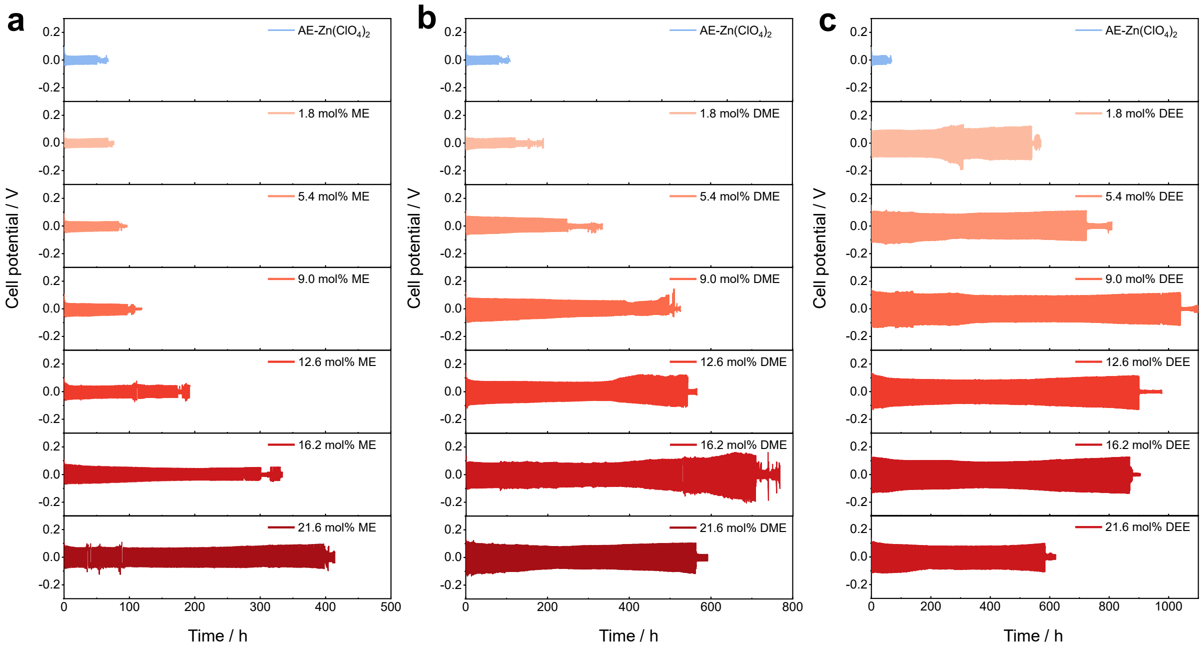

Supplementary Figure 65. Cycle performance of symmetric Zn||Zn cells at 5 mA cm^{-2} and 5 mAh cm^{-2} with 3 m $Zn(ClO_4)_2$ aqueous electrolyte solutions containing various organic additives in different concentrations: (**a**) ME, (**b**) DME and (**c**) DEE.

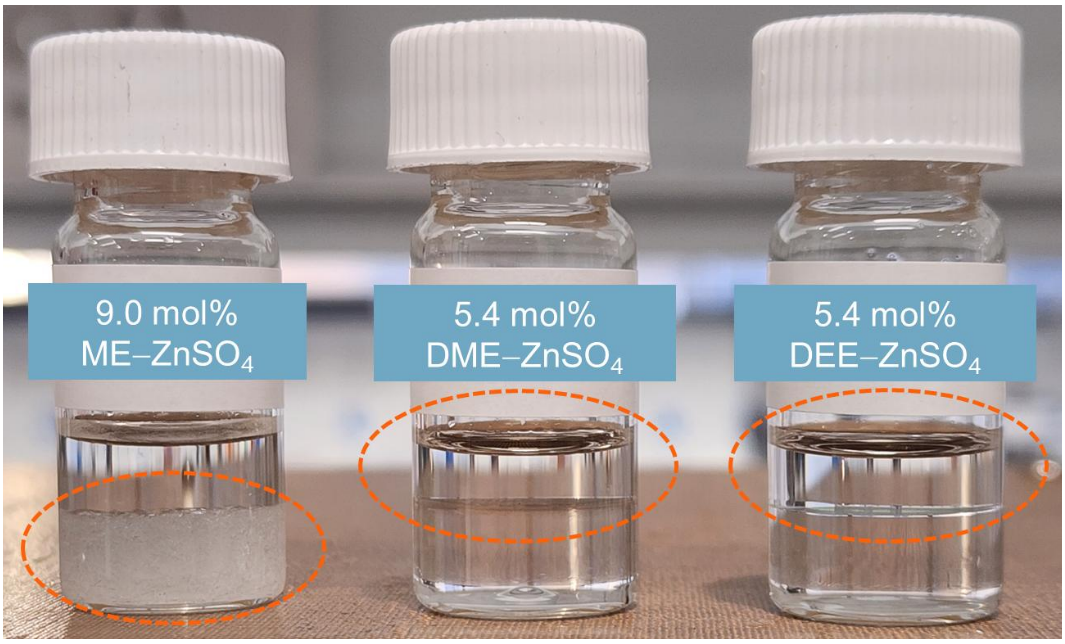

Supplementary Figure 66. Photographic pictures of the 3 m $ZnSO_4$ electrolyte solutions containing ME, DME and DEE organic additives in different concentrations, showing the maximum ether additive concentration in the 3 m $ZnSO_4/H_2O$ electrolyte system. In water-salt-organic ternary phase systems, the $ZnSO_4$-derived ionic species and organic additive both compete to coordinate with water molecules. This competition derives from the strong hydration ability of SO_4^{2-} anion and affects the overall phase stability of the ternary phase system. As shown in Supplementary Fig. 66, DME and DEE phases physically separate from the water solution as $ZnSO_4$-derived species occupy almost all coordination sites of water molecules and weaken the solvation capacity of water with other molecules, such as organic species. In contrast, $ZnSO_4$ salt precipitation occurs in the ME system because ME exhibits a stronger hydration ability, enabling preferential coordination with water molecules.

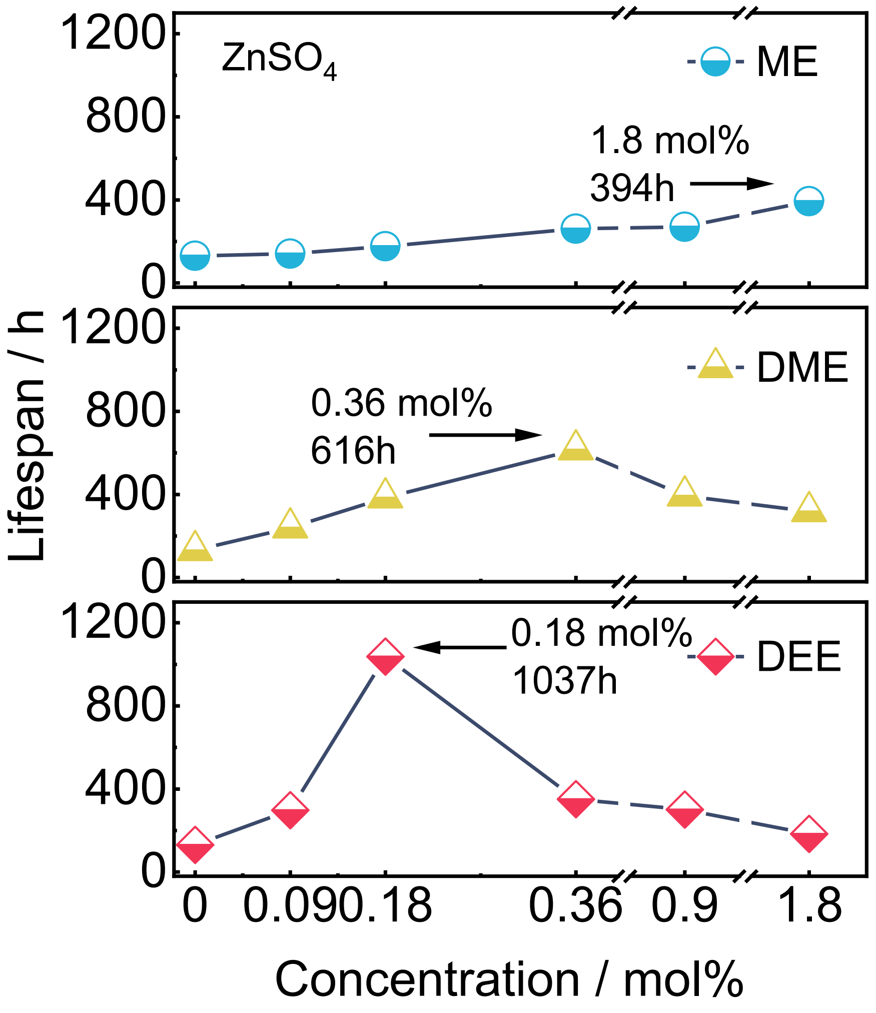

Supplementary Figure 67. Correlation between the cycling performance of symmetric Zn||Zn coin cells and concentrations of ME, DME and DME organic additives in aqueous 3 m $ZnSO_4$ electrolyte solutions.

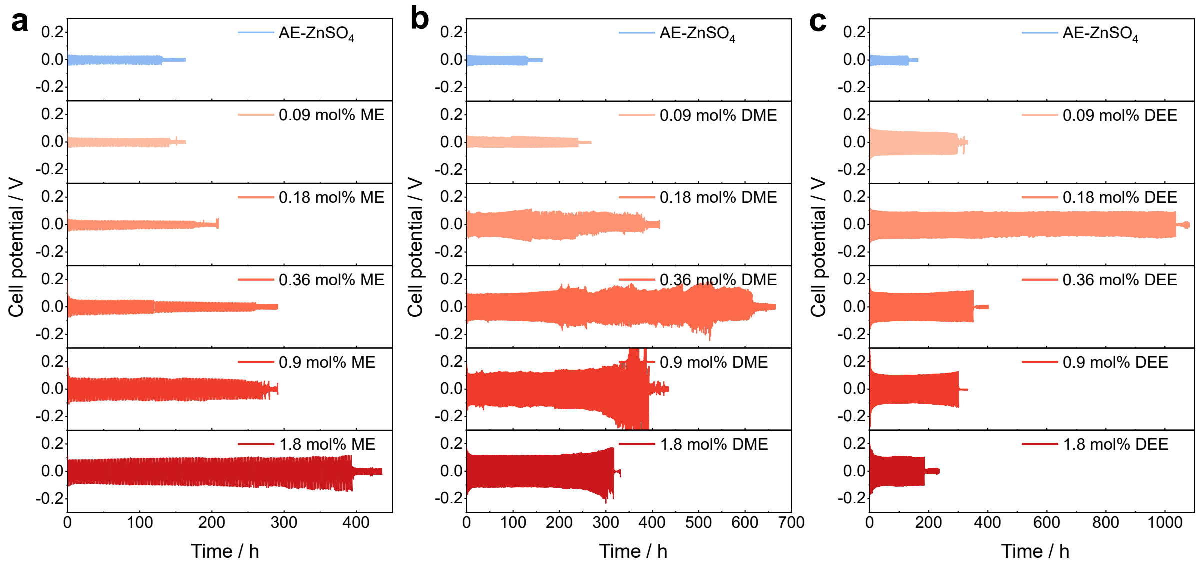

Supplementary Figure 68. Cycle performance of symmetric Zn||Zn cells at 5 mA cm^{-2} and 5 mA h cm^{-2} with 3 m $ZnSO_4$ aqueous electrolyte solutions containing various organic additives in different concentrations: (**a**) ME, (**b**) DME and (**c**) DEE.

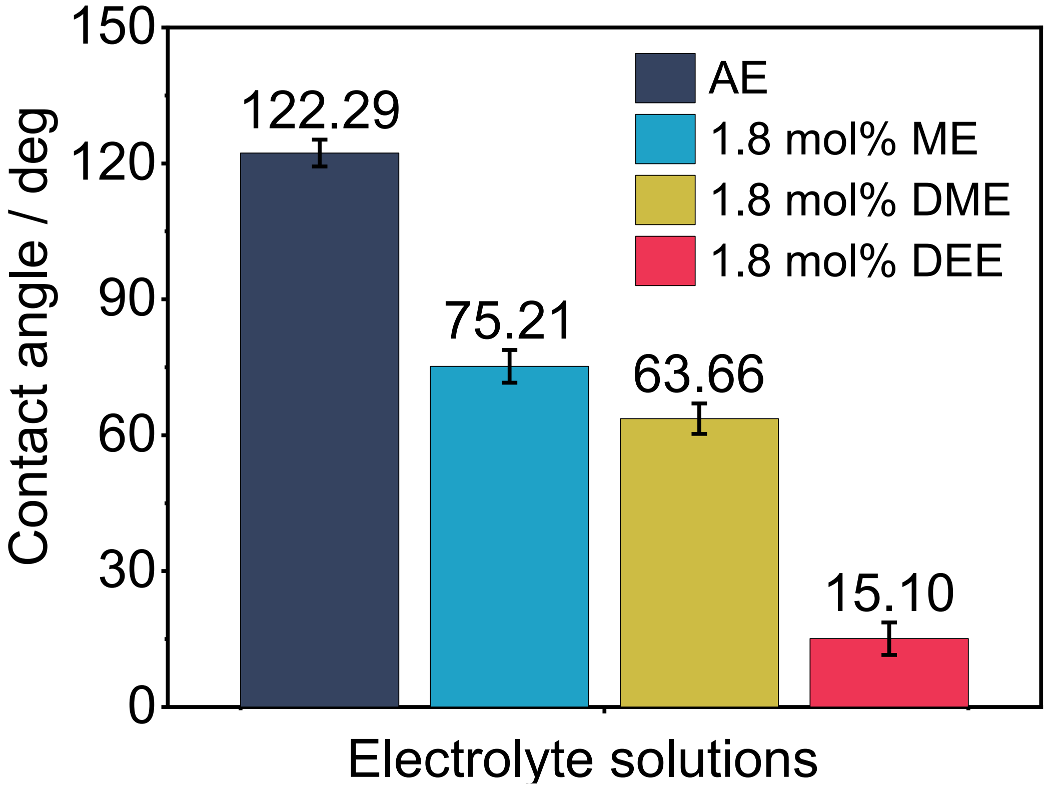

Supplementary Figure 69. Contact angle values obtained between NaV_3O_8 electrode and 3 m $Zn(OTf)_2$ electrolyte solutions with ME, DME and DEE organic additives at a concentration of 1.8 mol%. The error bar represents the standard deviation based on three independent measurements.

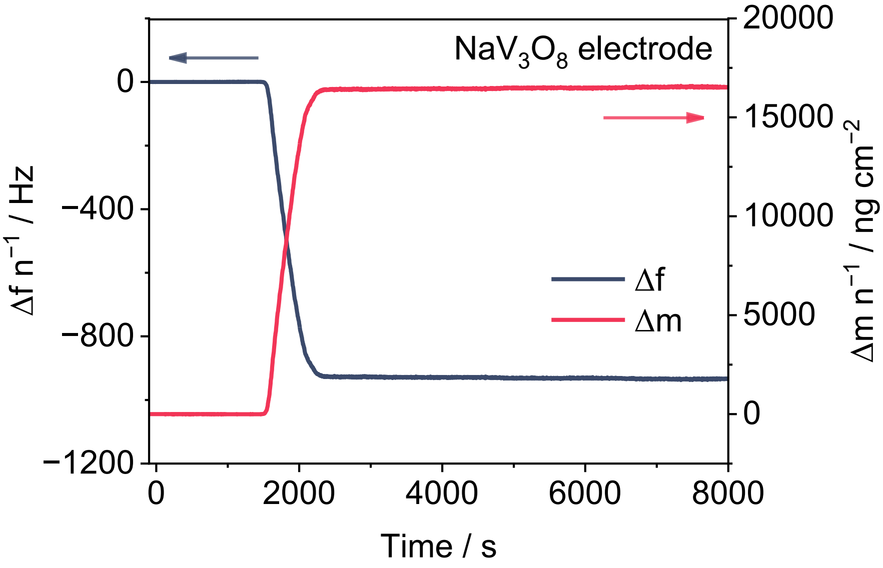

Supplementary Figure 70. QCM analysis of the NaV_3O_8-based positive electrode in the 3 m $Zn(OTf)_2$ electrolyte solution with 1.8 mol% DEE additive. Δf, the frequency change and Δm, the mass change after the electrolyte injection.

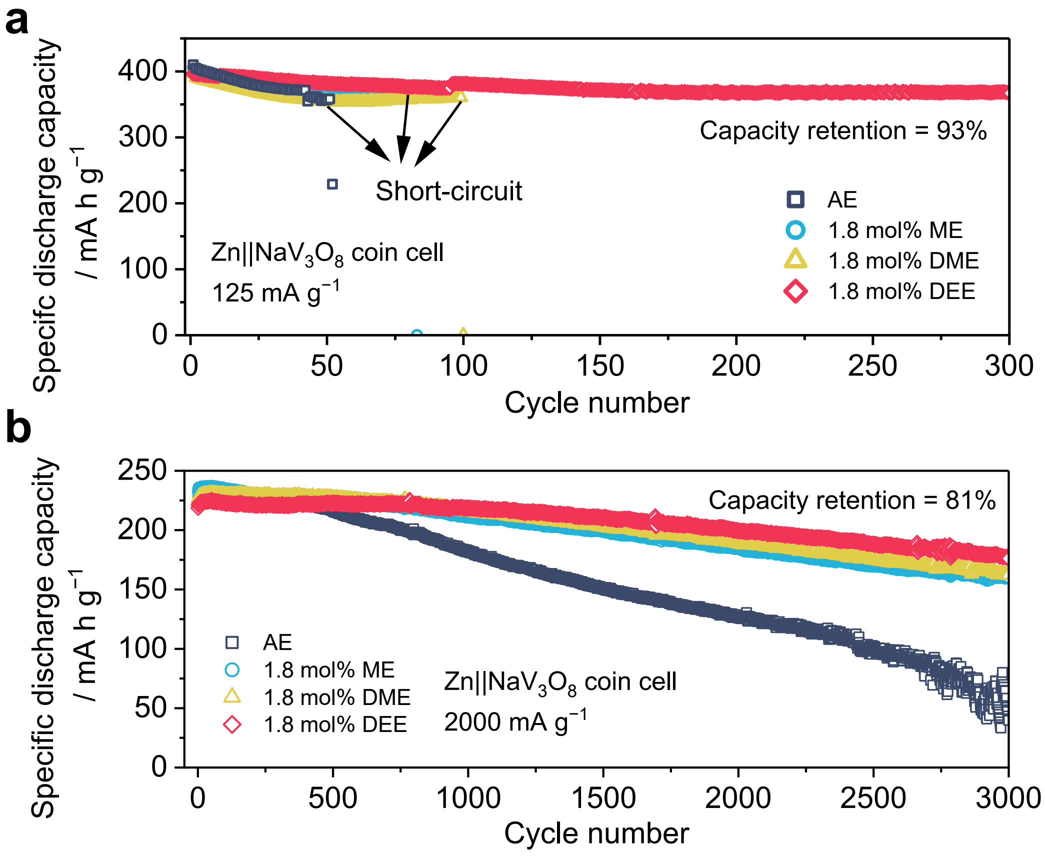

Supplementary Figure 71. Cycle performance of Zn||NaV_3O_8 coin cells at (**a**) 125 mA g^{-1} and (**b**) 2000 mA g^{-1} in AE, 1.8 mol% ME, 1.8 mol% DME and 1.8 mol% DEE electrolyte solutions at 25 °C. The capacity retention is calculated based on the maximum discharge capacity during cycling, because of the activation process commonly observed in Zn metal cells with vanadium-based positive electrodes, where the capacity gradually increases during the initial cycles. In panel a, for the coin cell with the 1.8 mol% DEE electrolyte solution, an increase in discharge capacity is observed after about 90 cycles. This effect is due to a temperature change in the test room following a power outage.

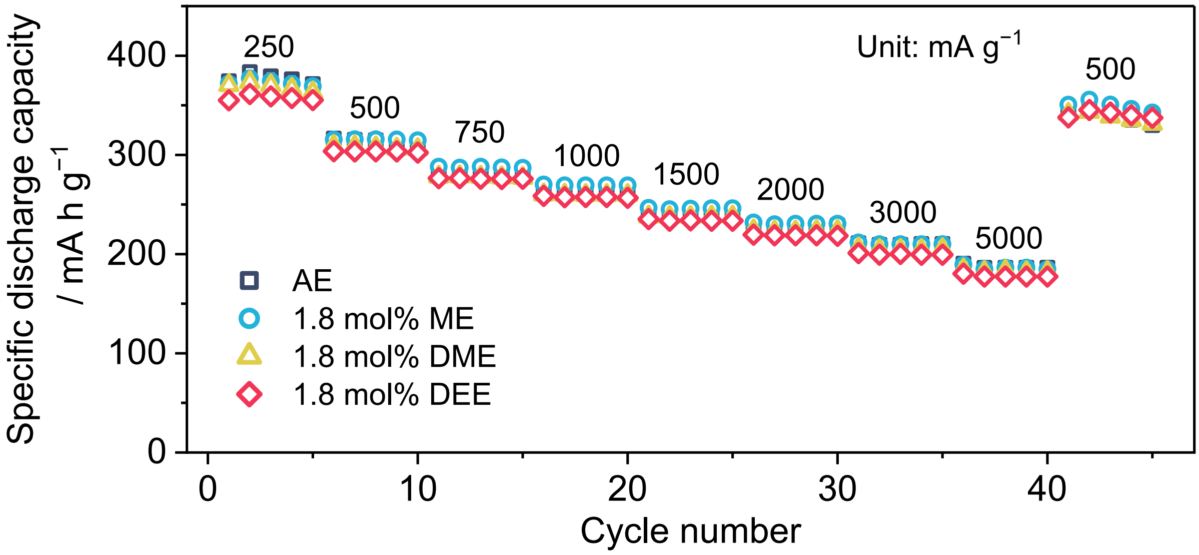

Supplementary Figure 72. Rate capability of Zn||NaV_3O_8 coin cells in AE, 1.8 mol% ME, 1.8 mol% DME and 1.8 mol% DEE electrolyte solutions at 25 °C.

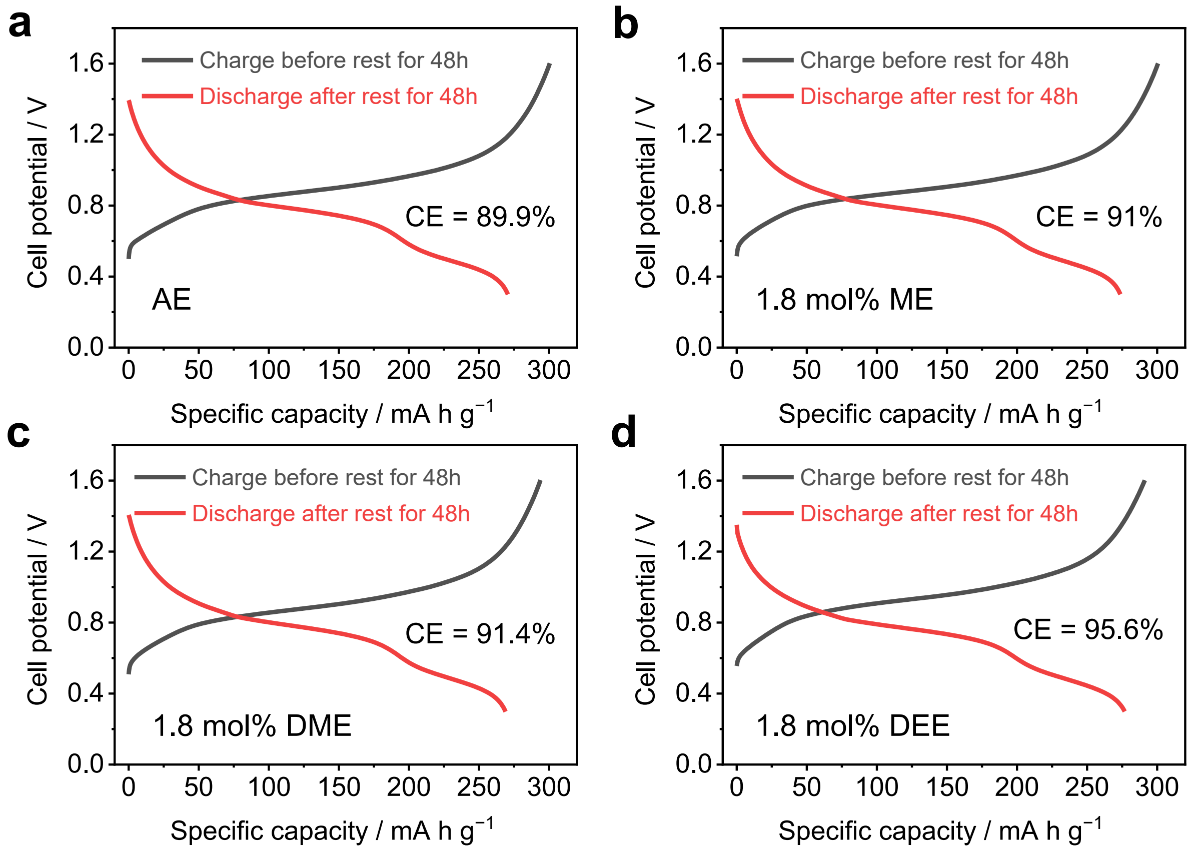

Supplementary Figure 73. Charge/discharge profile at 25 °C of Zn||NaV_3O_8 coin cells during the self-discharge using various electrolyte solutions: (**a**) baseline AE, (**b**) 1.8 mol% ME, (**c**) 1.8 mol% DME and (**d**) 1.8 mol% DEE.

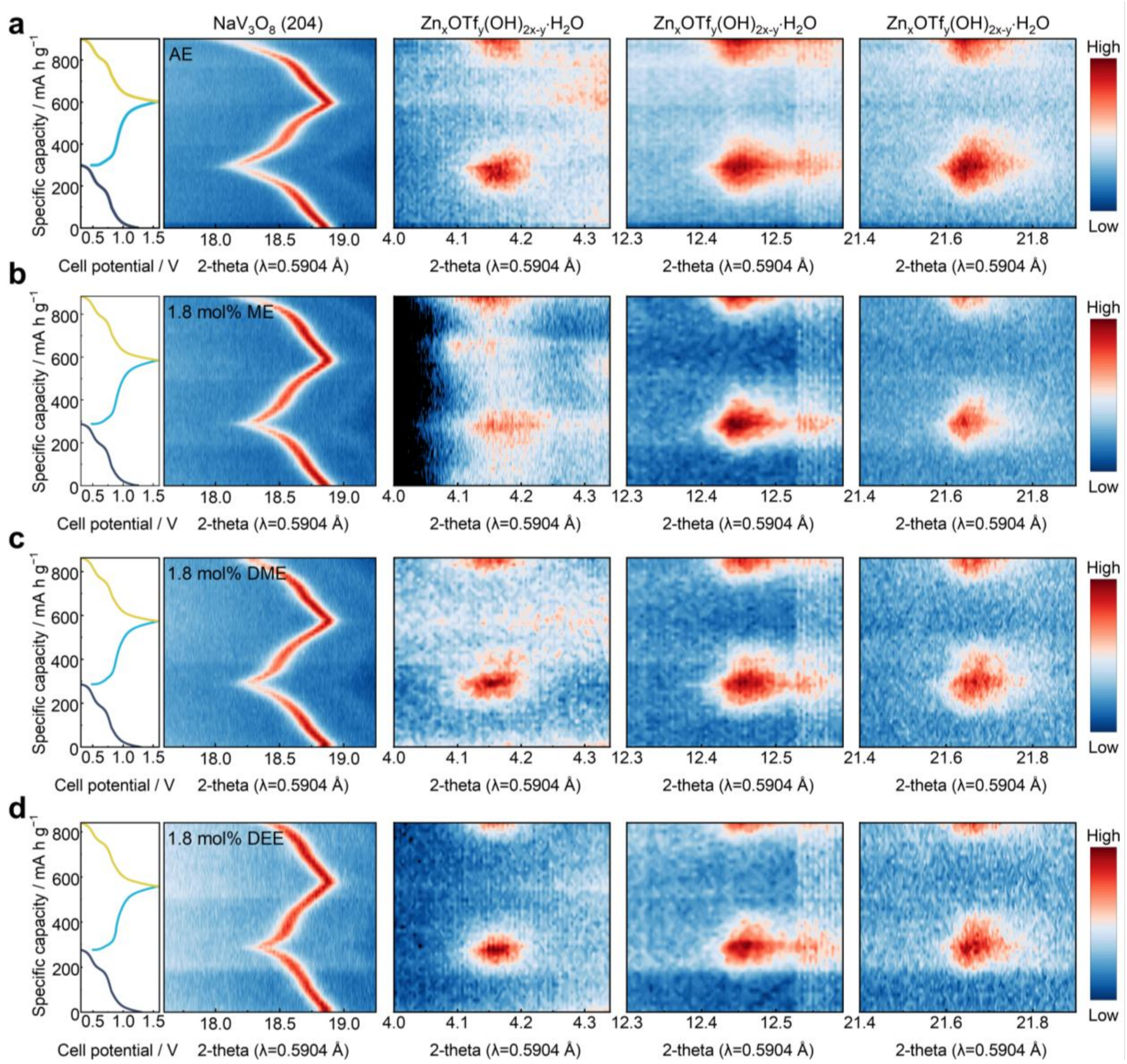

Supplementary Figure 74. Contour plot of NaV_3O_8 (204) and $Zn_xOTf_y(OH)_{2x-y}$ H_2O (BZS) reflections evolution obtained via operando Synchrotron-based XRPD measurements carried out during Zn||NaV_3O_8 coin cell cycling using (**a**) AE, (**b**) 1.8 mol% ME, (**c**) 1.8 mol% DME and (**d**) 1.8 mol% DEE electrolyte solutions. The black, light blue and dark yellow potential profile curves correspond to the initial discharge, initial charge and second discharge, respectively. The colour bar represents the intensity of the XRPD patterns, with red indicating high intensity and blue indicating low intensity.

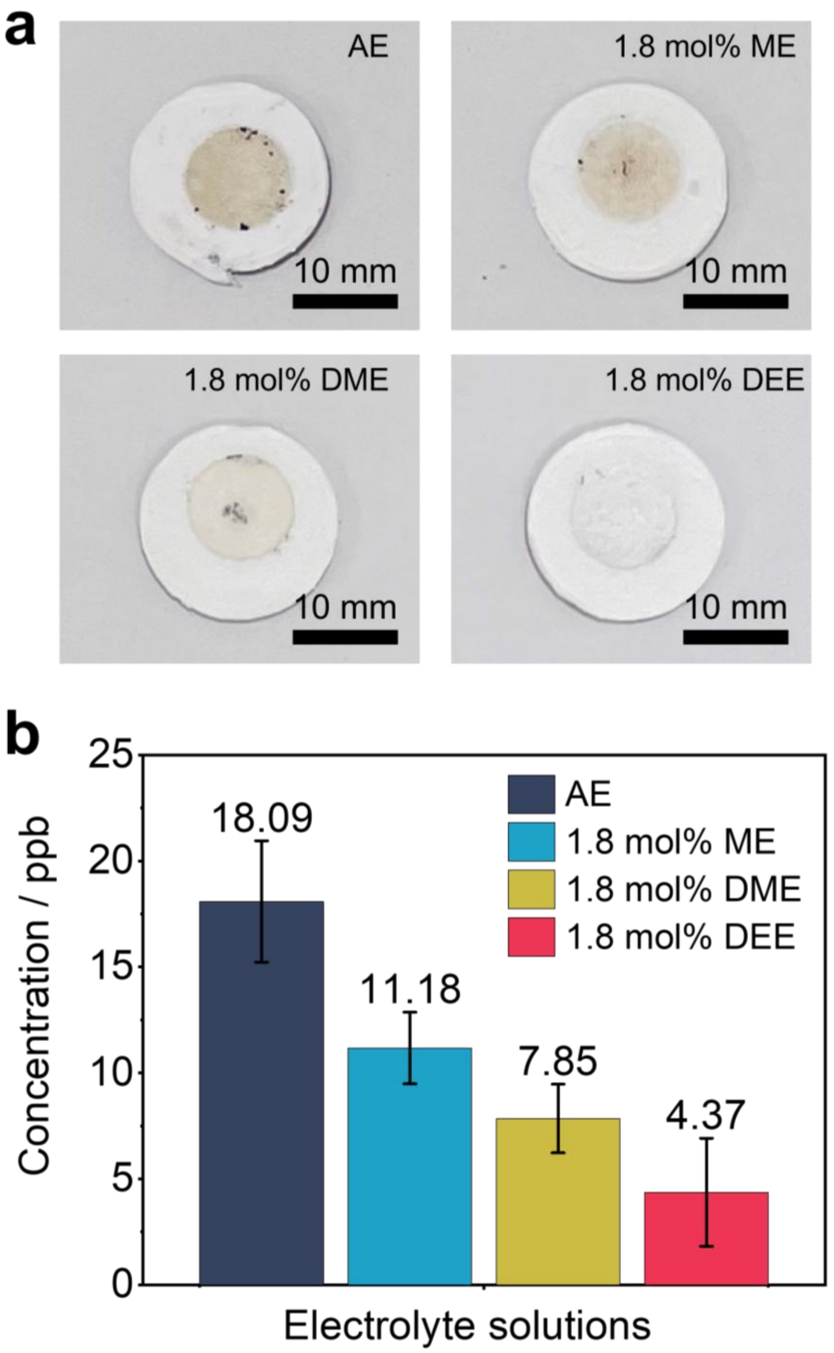

Supplementary Figure 75. (**a**) Photographic pictures of the glass fibre separators harvested from Zn||NaV_3O_8 coin cells after 200 cycles at 500 mA g^{-1} and 25 °C; (**b**) the concentration of dissolved vanadium ions (*e.g.*, VO_2^+, $[VO_2(OH)_2]^-$) in the separator. The error bar represents the standard deviation based on three independent measurements. The dissolved vanadium from the NaV_3O_8 in the positive electrode deposits on the separator, as suggested by the yellow deposits. The separator harvested from the coin cell cycled using the 1.8 mol% DEE electrolyte solution maintains its white colour, suggesting that vanadium dissolution is mitigated to some extent, as also supported by the results of inductively coupled plasma mass spectrometry measurements.

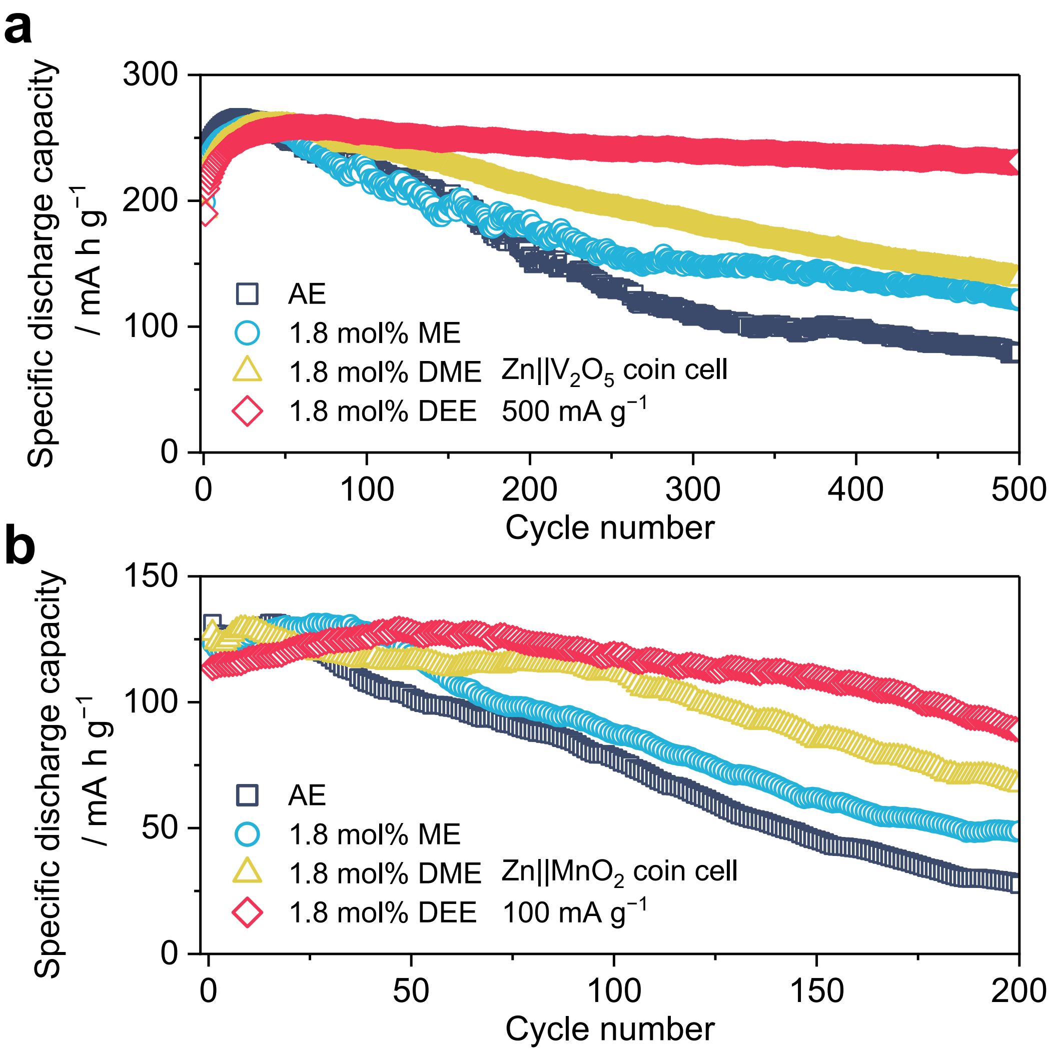

Supplementary Figure 76. Cycle performance of (**a**) Zn||V_2O_5 coin cells at 500 mA g^{-1} and 25 °C and (**b**) Zn||MnO_2 coin cells at 100 mA g^{-1} and 25 °C in AE, 1.8 mol% ME, 1.8 mol% DME and 1.8 mol% DEE electrolyte solutions. As shown in panel a, the Zn||V_2O_5 cell shows an improved cycling stability when using the 1.8 mol% DEE electrolyte solution, retaining 89% of its initial specific discharge capacity after 500 cycles at 500 mA g^{-1} and 25 °C, compared to the 30%, 47%, and 53% for the same cell configuration with the baseline AE, 1.8 mol% ME, and 1.8 mol% DME, respectively. Similarly, in panel b, the Zn||MnO_2 coin cell exhibits a higher capacity retention of 69% when the 1.8 mol% DEE electrolyte is used, whereas the retention with the baseline AE is only 21% under the same conditions.

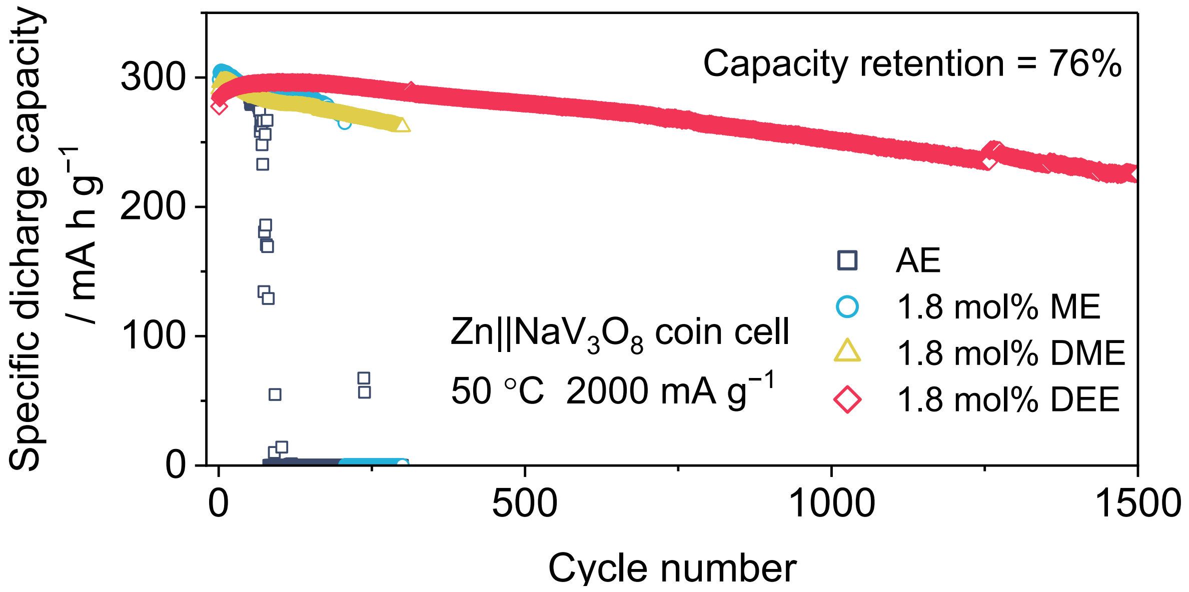

Supplementary Figure 77. Cycle performance of Zn||NaV_3O_8 coin cells at 2000 mA g^{-1} and 50 °C using the baseline AE, 1.8 mol% ME, 1.8 mol% DME, and 1.8 mol% DEE electrolyte solutions. The capacity retention is calculated based on the maximum discharge capacity during cycling. After about 1250 cycles, for the coin cell with the 1.8 mol% DEE electrolyte solution, it can be observed an increase in discharge capacity. This effect is due to a temperature change in the test room following a power outage.

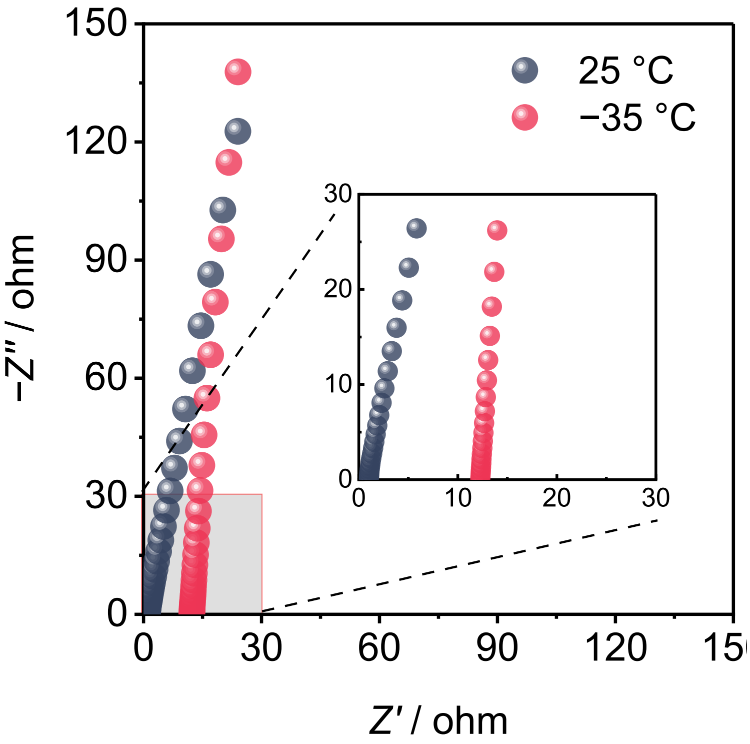

Supplementary Figure 78. Nyquist plot of SS||SS symmetric cells containing the 1.8 mol% DEE electrolyte solution. Electrochemical impedance measurements are carried out at 25 °C and −35 °C. The bulk ionic conductivity of the electrolyte solution at −35 °C was calculated using the following equation (Eq. 3):

$$\sigma = \frac{L}{SR} = \frac{K_{cell}}{R} \qquad (3)$$

where K_{cell} is the cell constant determined from the bulk ionic conductivity measured at 25 °C using a conductivity meter, L is the distance between two SS electrodes, S is the contact area of the SS electrodes, and R is the resistance value in Ohms extrapolated at the intersection between the raw EIS data and the real impedance axis.

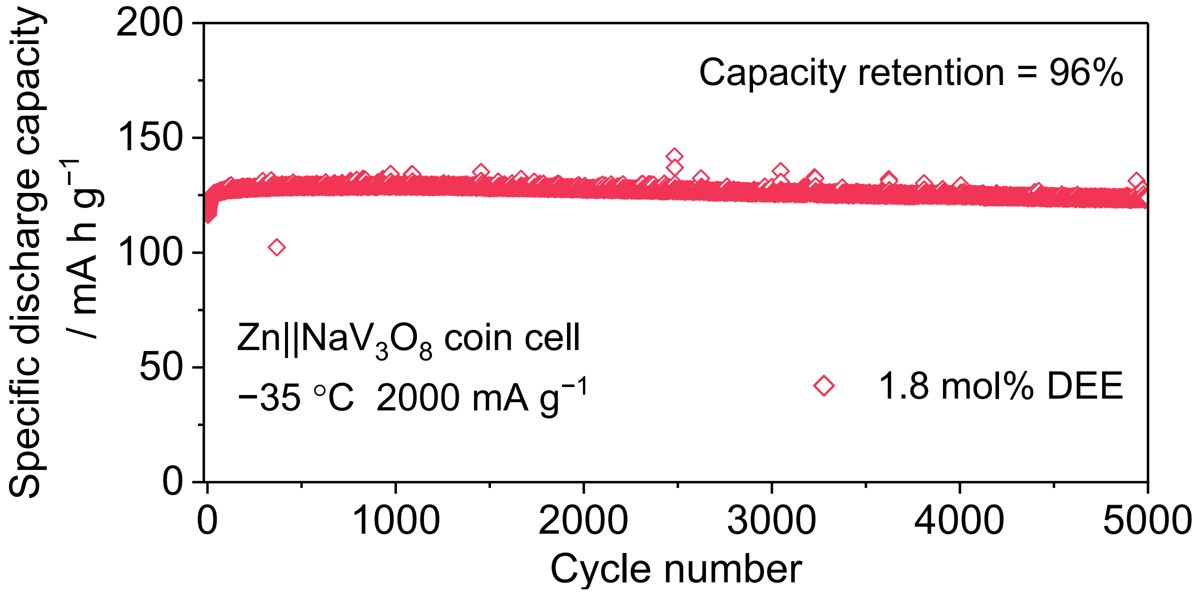

Supplementary Figure 79. Cycle performance of Zn||NaV_3O_8 coin cells at 2000 mA g^{-1} and −35 °C with the 1.8 mol% DEE electrolyte solution.

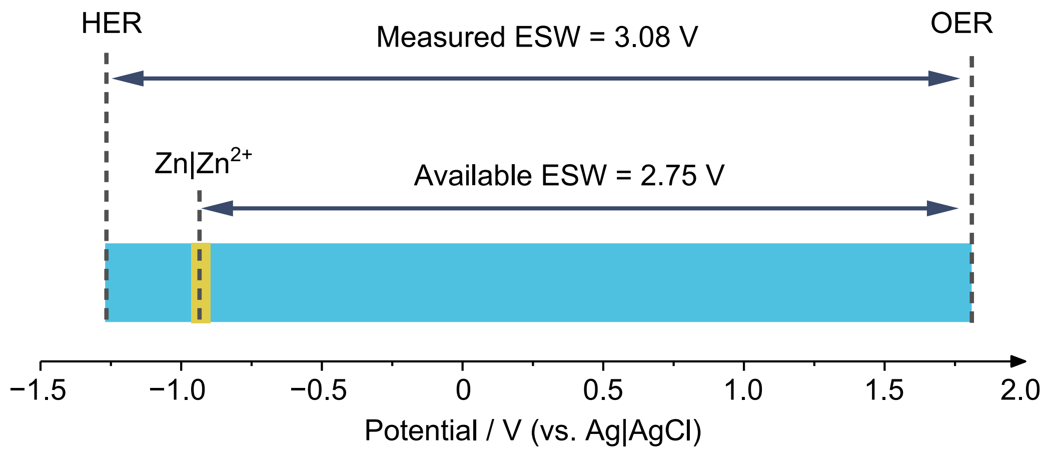

Supplementary Figure 80. Schematic illustration of the available ESW of the LEI electrolyte (1.8 mol% DEE) in AZBs using Zn metals as the negative electrode. The measured ESW and $Zn|Zn^{2+}$ electrode potentials were determined by DEMS measurements (Supplementary Fig. 30).

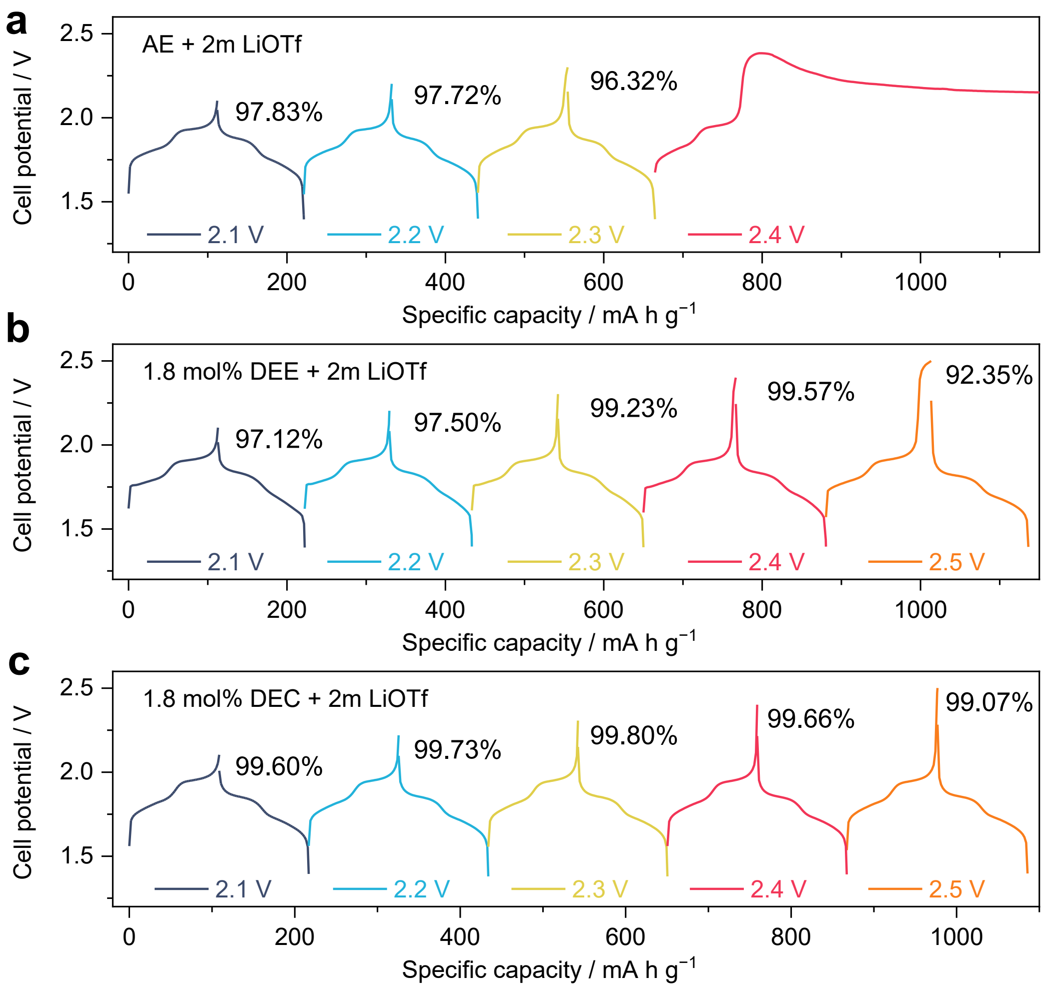

Supplementary Figure 81. Charge-discharge profile of Zn||$LiMn_2O_4$ coin cells under different upper cut-off potentials at 300 mA g^{-1} and 25 °C using various electrolyte solutions: (a) baseline AE + 2 m LiOTf, (**b**) 1.8 mol% DEE + 2 m LiOTf and (**c**) 1.8 mol% DEC + 2 m LiOTf.

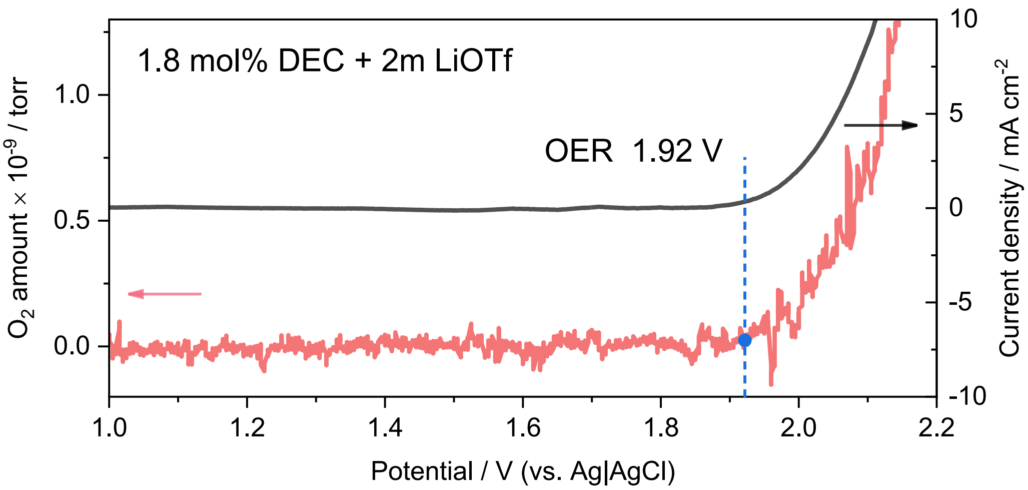

Supplementary Figure 82. Oxygen evolution potential of the 1.8 mol% DEC + 2m LiOTf electrolyte solution, measured using a gold working electrode, an Ag|AgCl reference electrode, and a platinum counter electrode at a scan rate of 5 mV s^{-1} via DEMS.

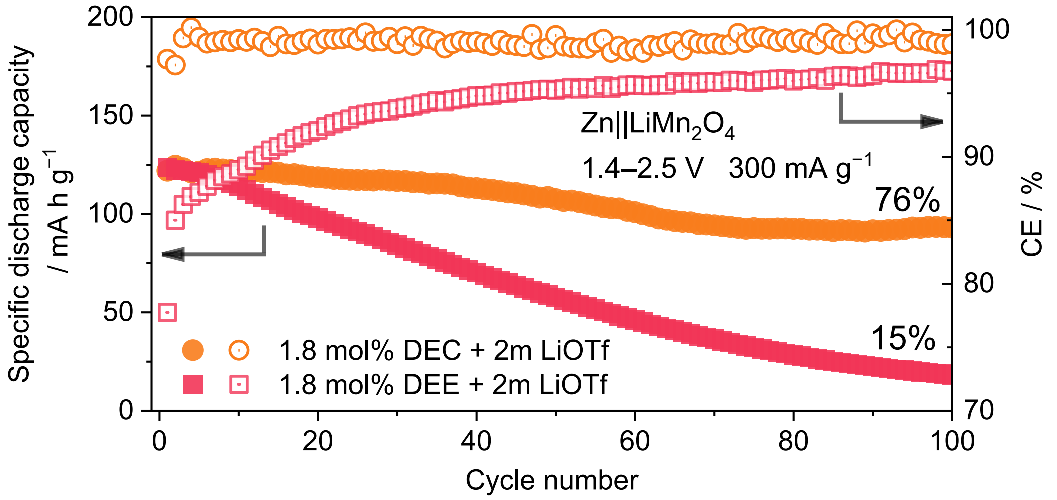

Supplementary Figure 83. Cycle performance of Zn||LiMn$_2$O$_4$ coin cells at 300 mA g^{-1} and 25 °C using 1.8 mol% DEE + 2 m LiOTf and 1.8 mol% DEC + 2 m LiOTf electrolyte solutions. Zn||LiMn$_2$O$_4$ coin cells with 1.8 mol% DEC + 2 m LiOTf this electrolyte demonstrates 76% specific discharge capacity retention after 100 cycles, which is higher than 15% obtained using 1.8 mol% DEE + 2 m LiOTf.

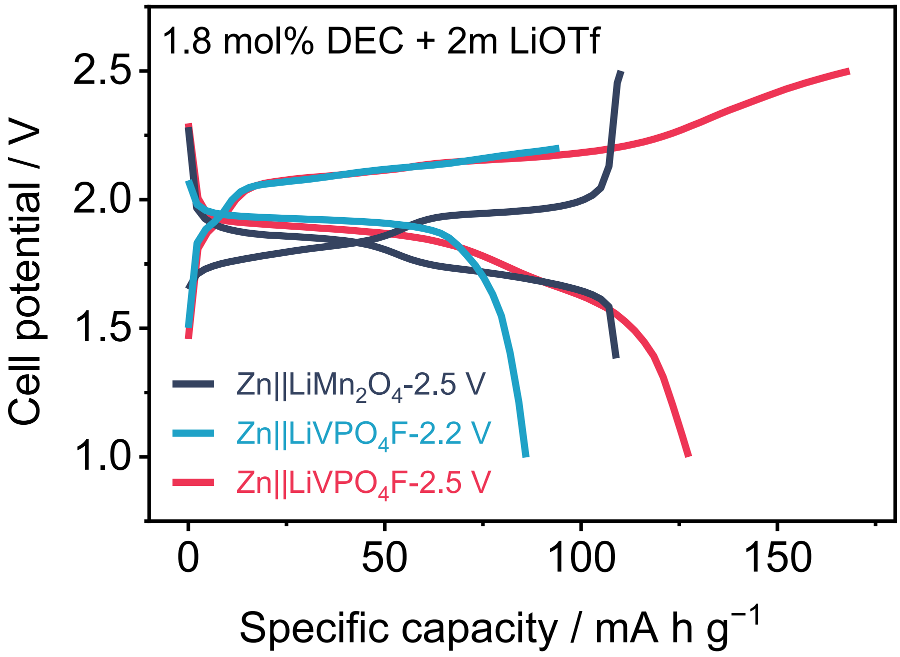

Supplementary Figure 84. Potential profiles of Zn||$LiMn_2O_4$ and Zn||$LiVPO_4F$ coin cells under different upper cut-off potentials at 300 mA g^{-1} and 25 °C using the 1.8 mol% DEC + 2 m LiOTf electrolyte solution.

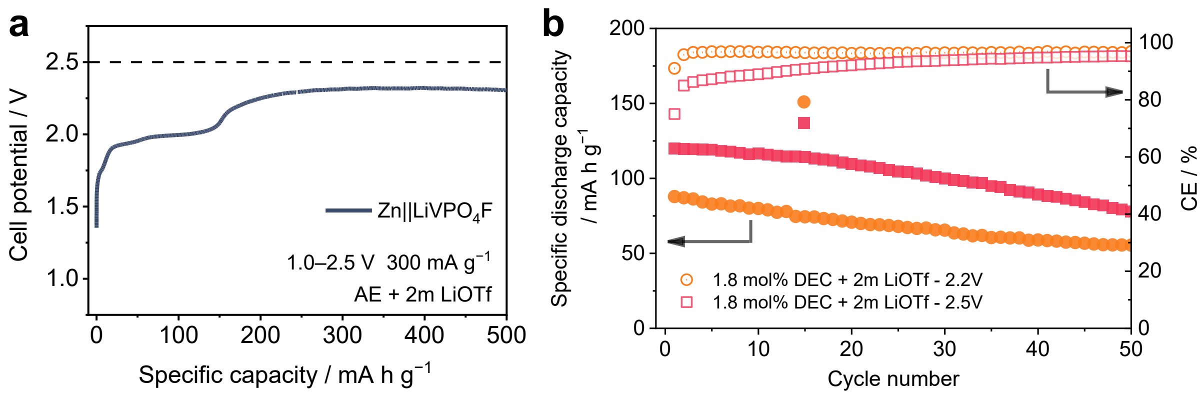

Supplementary Figure 85. Cycle performance of Zn||$LiVPO_4F$ coin cells using (a) AE + 2 m LiOTf and (**b**) 1.8 mol% DEC + 2 m LiOTf electrolyte solutions under different upper cut-off potentials at 300 mA g^{-1} and 25 °C.

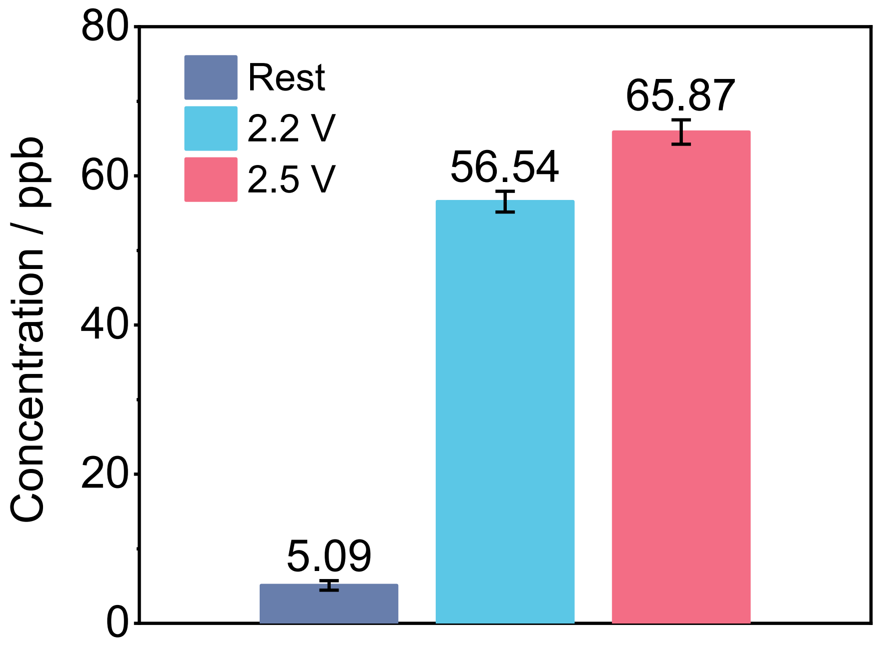

Supplementary Figure 86. ICP-MS results of the concentration of dissolved vanadium ions in the glass fibre separators harvested from Zn||$LiVPO_4F$ coin cells after 50 cycles at 300 mA g^{-1} within potential ranges of 1.0–2.2 V or 1.0–2.5 V using the 1.8 mol% DEC + 2 m LiOTf electrolyte solution.

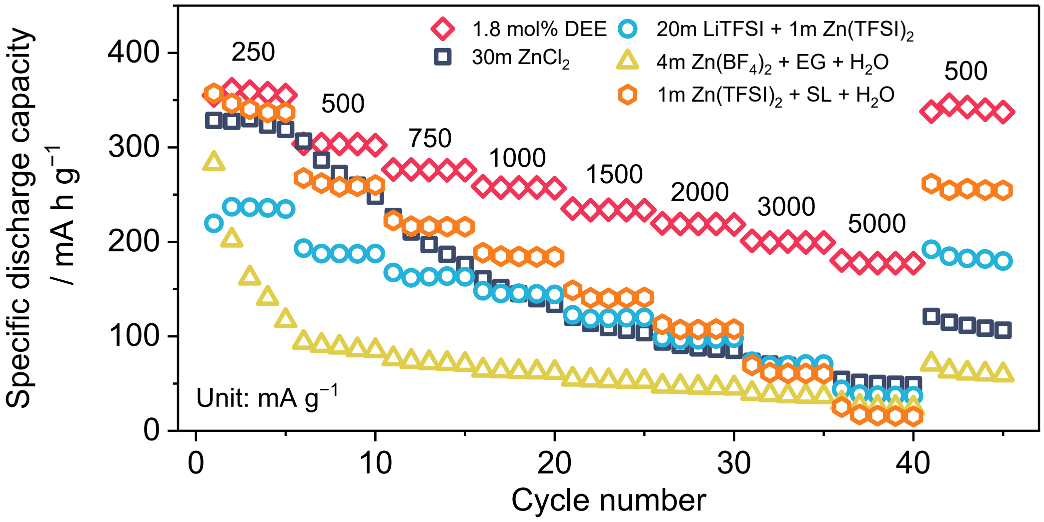

Supplementary Figure 87. Rate capability of Zn||NaV_3O_8 coin cells in 1.8 mol% DEE electrolyte solution, HCEs (30 m $ZnCl_2$, 20 m LiTFSI + 1 m $Zn(TFSI)_2$) and OAHEs (4 m $Zn(BF_4)$ + EG + H_2O, 1 m $Zn(TFSI)_2$ + SL + H_2O) tested at 25 °C.

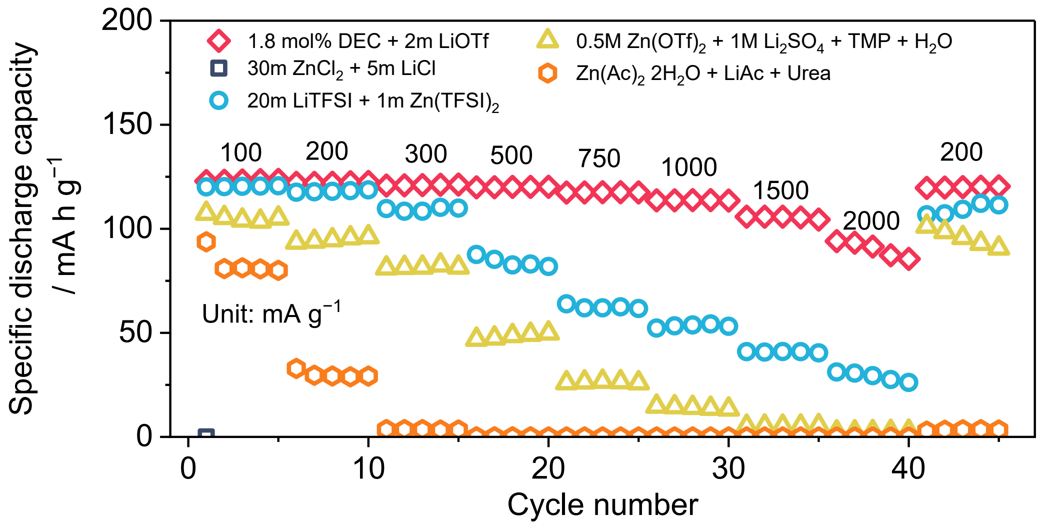

Supplementary Figure 88. Rate capability of Zn||$LiMn_2O_4$ coin cells in 1.8 mol% DEC + 2 m LiOTf electrolyte solution, HCEs (30 m $ZnCl_2$ + 5m LiCl, 20 m LiTFSI + 1 m $Zn(TFSI)_2$) and OAHEs (0.5M $Zn(OTf)_2$ + 1M Li_2SO_4 + trimethyl phosphate (TMP) + H_2O, $Zn(Ac)_2$ $2H_2O$ + LiAc + Urea) tested at 25 °C.

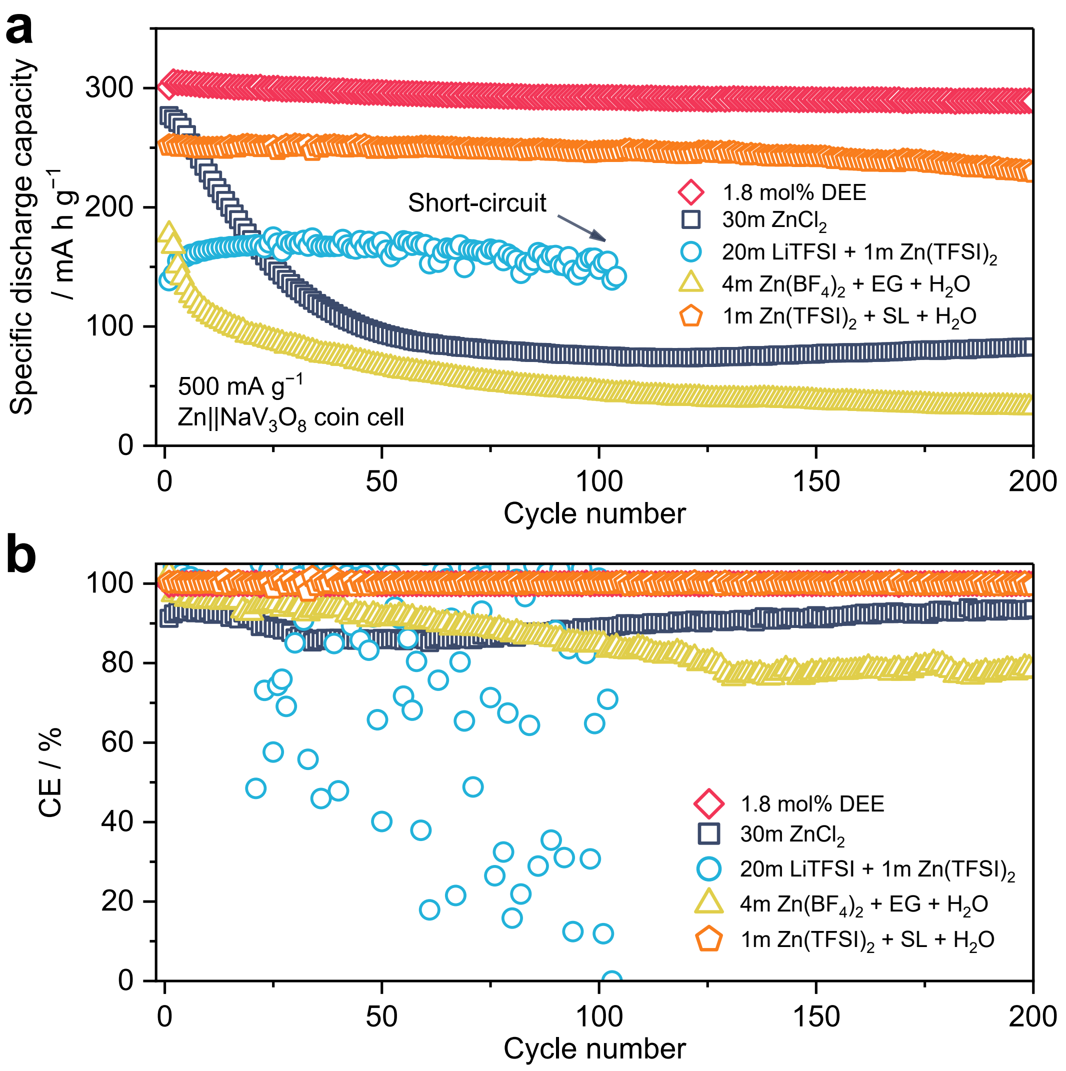

Supplementary Figure 89. (**a**) Cycle performance and (**b**) corresponding CE of Zn||NaV_3O_8 coin cells tested at 500 mA g^{-1} and 25 °C using 1.8 mol% DEE electrolyte solution, HCEs (30 m $ZnCl_2$, 20 m LiTFSI + 1 m $Zn(TFSI)_2$) and OAHEs (4 m $Zn(BF_4)$ + EG + H_2O, 1 m $Zn(TFSI)_2$ + SL + H_2O).

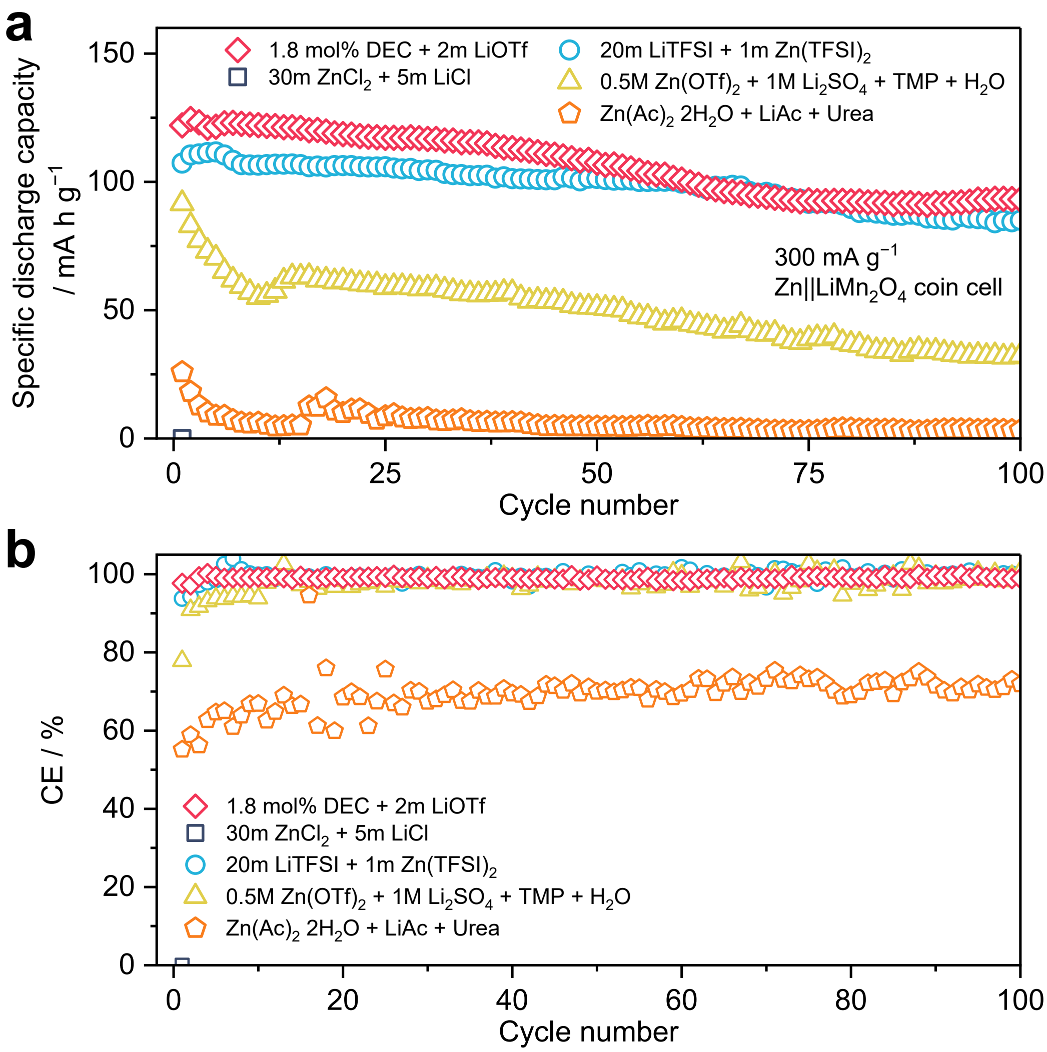

Supplementary Figure 90. (**a**) Cycle performance and (**b**) corresponding CE of Zn||$LiMn_2O_4$ coin cells tested at 300 mA g^{-1} and 25 °C using 1.8 mol% DEC + 2 m LiOTf electrolyte solution, HCEs (30 m $ZnCl_2$ + 5m LiCl, 20 m LiTFSI + 1 m $Zn(TFSI)_2$) and OAHEs (0.5M $Zn(OTf)_2$ + 1M Li_2SO_4 + TMP + H_2O, $Zn(Ac)_2$ $2H_2O$ + LiAc + Urea).

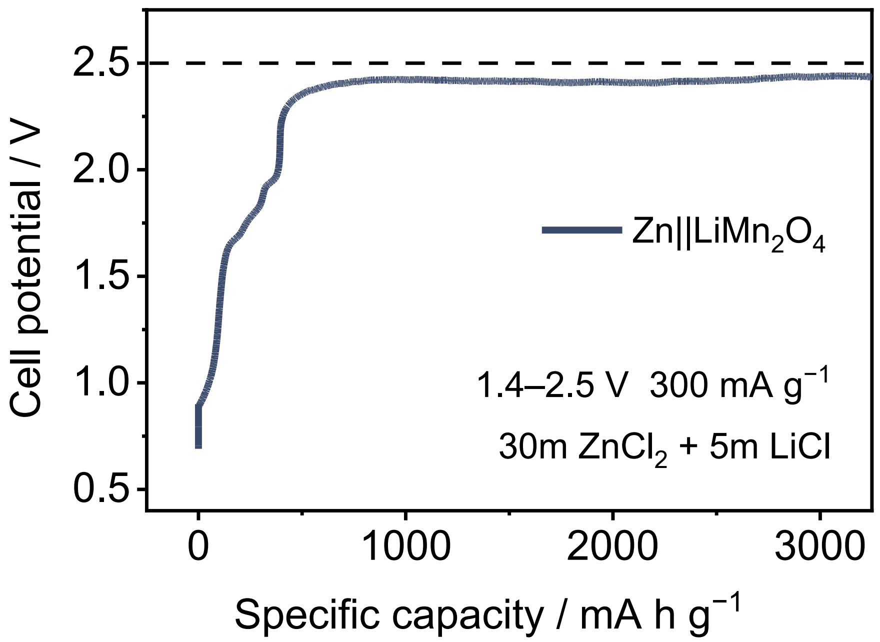

Supplementary Figure 91. First charge potential profile of Zn||$LiMn_2O_4$ coin cells using 30 m $ZnCl_2$ + 5 m LiCl with an upper cut-off potential of 2.5 V at 300 mA g^{-1} and 25 °C.

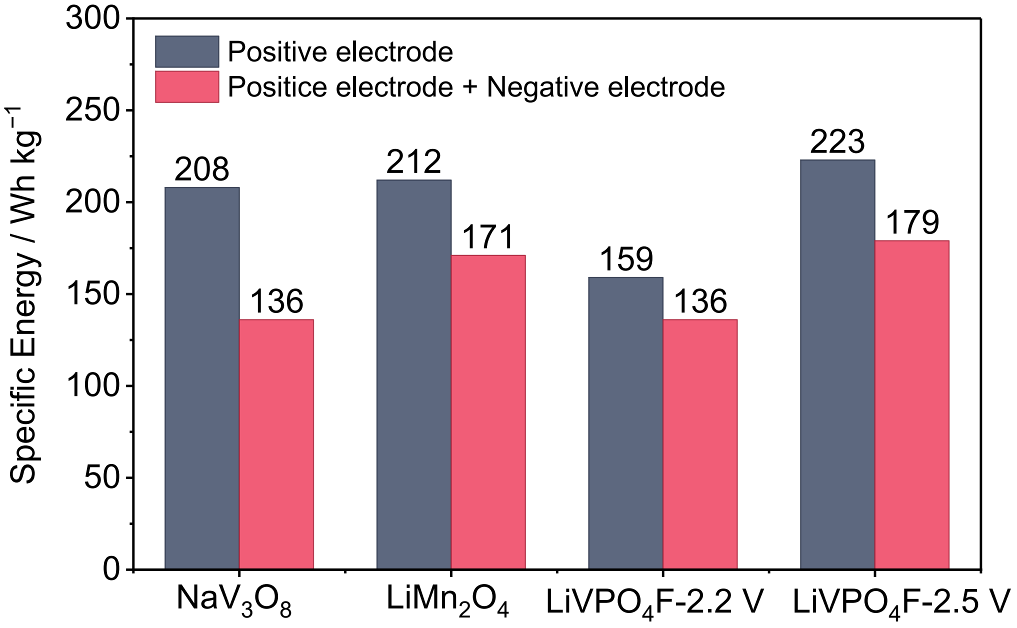

Supplementary Figure 92. Calculated electrode-level specific energy of Zn||NaV_3O_8, Zn||$LiMn_2O_4$ and Zn||$LiVPO_4F$ cells based on a N/P ratio of 1.60. The electrode-level specific energy was calculated considering only the active materials in the positive electrodes. For Zn||NaV_3O_8 cells (pouch cells), the values were derived from the initial charge–discharge cycle at 25 °C within a potential range of 0.3–1.6 V at a current density of 500 mA g^{-1}. For Zn||$LiMn_2O_4$ cells (coin cells), the values were obtained from the initial cycle at 25 °C within a potential range of 1.4–2.5 V at 300 mA g^{-1}. For Zn|| $LiVPO_4F$ cells (coin cells), the values were obtained from the initial cycle at 25 °C within a voltage range of 1.0–2.2 V or 1.0–2.5 V at 300 mA g^{-1}.

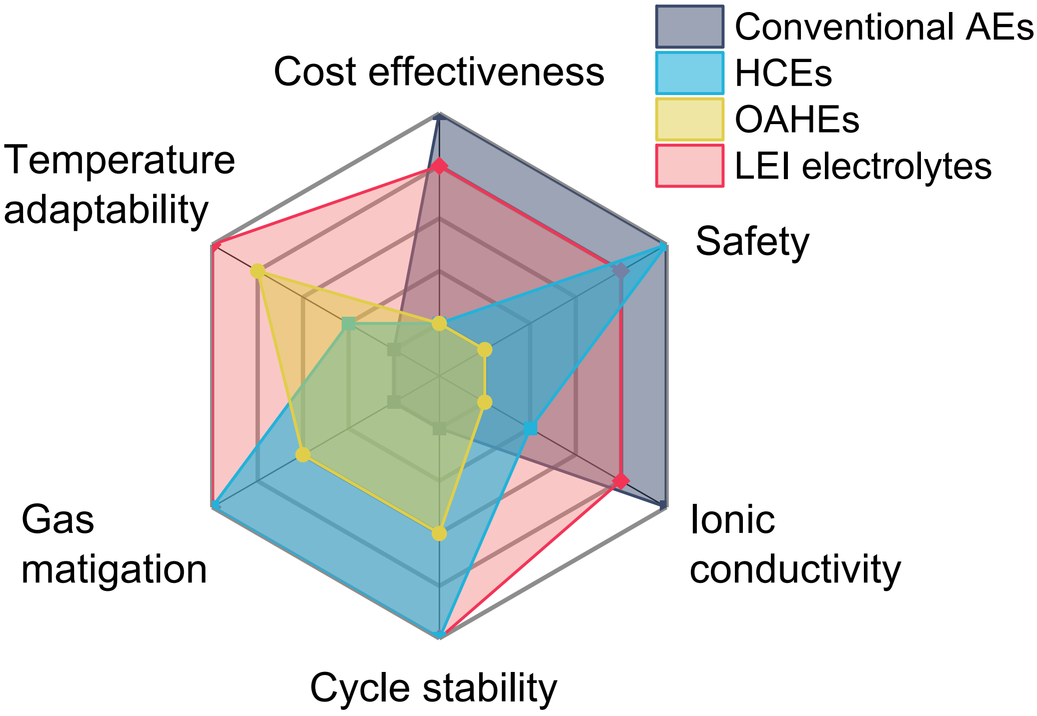

Supplementary Figure 93. Radar plot showing the advantages of DEE-supported LEI-based electrolyte compared to conventional aqueous electrolytes (AEs), HCEs and OAHEs.

Supplementary Table 1. Physical properties of the organic additives studied in this work, measured or taken from the literature at 25 °C. The average CE was obtained from asymmetric Zn||Cu coin cells using glass fibre membranes tested at 1 mA cm^{-2}, 1 mA h cm^{-2} and 25 °C for 100 cycles using 3 m $Zn(OTf)_2$ electrolyte solutions containing various organic additives at a concentration of 1.8 mol% for a single organic additive. The contact angle between the zinc metal negative electrode and electrolyte solutions was determined at 25°C in air.

**Solvent**	**Contact angle / °**	**Log $P_{oct/wat}$**	**Donor number (kcal mol^{-1})**	**Dielectric constant**	**Average CE / %**
Water	89.75	/	18	78.5	74.06
2-Methoxyethanol (ME)	85.85	-0.77	19.7	16.9	85.27
1,2-Dimethoxyethane (DME)	70.74	-0.21	20	7.2	98.27
1,2-Diethoxyethane (DEE)	54.28	0.66	13.2	3.9	99.32
Diglyme (G2)	74.97	-0.36	19.5	7.2	98.13
1,4-Dioxane (1,4-DX)	83.47	-0.42	14.8	2.2	97.22
1,3-Dioxolane (DOL)	88.05	-0.37	18	7.1	90.68
Methanol (MeOH)	88.94	-0.82	19	32.6	75.66
Ethanol (EtOH)	85.59	-0.32	19	24.6	80.25
1-Butanol (n-BuOH)	43.96	0.88	19.5	17.8	98.53
Dimethyl carbonate (DMC)	86.95	0.23	15.1	3.1	87.56
Diethyl carbonate (DEC)	58.57	1.21	16	3.1	99.15
Ethylene carbonate (EC)	83.87	0.11	16.4	89.1	96.5
Propylene carbonate (PC)	87.85	-0.41	15.1	64	89.95
γ-Butyrolactone (GBL)	81.4	-0.64	18	39	96.9
N,N-Dimethylformamide (DMF)	81.97	-0.74	26.6	36.1	98.18
Acetonitrile (AN)	86.88	-0.34	14	38.8	86.55
Trimethyl phosphate (TMP)	76.55	-0.78	23	21.6	97.71
Dimethyl sulfoxide (DMSO)	77.51	-1.35	29.8	45	96.42
Sulfolane (SL)	75.15	-0.77	14.8	42	98.18

Supplementary Table 2. The ratio of C-O-C_{ether}/H_2O_{ads} obtained from O *1s* NAP-XPS spectra of Zn metal electrodes immersed with 3 m $Zn(OTf)_2$ electrolyte solutions containing 1.8 mol % of ME, DME and DEE additives. The ratio is calculated based on the integral area of C-O-C_{ether} and H_2O_{ads} deconvoluted peaks.

**Electrolytes**	**S(C-O-C_{ether})**	**S(H_2O_{ads})**	**S(C-O-C_{ether})/S(H_2O_{ads})**
3 m $Zn(OTf)_2$ + 1.8 mol% ME	100.5	130.4	0.77
3 m $Zn(OTf)_2$ + 1.8 mol% DME	126.8	99.8	1.27
3 m $Zn(OTf)_2$ + 1.8 mol% DEE	297.9	86.4	3.45

Supplementary Table 3. Comparison of optimal dosage, bulk ionic conductivity, lifespan and cumulative capacity of the DEE-containing LEI-forming electrolyte solution with previously reported electrolytes modified using organic additives.

Organic additive	Electrolyte solution	Optimal dosage of organic additive	Bulk ionic conductivity at 25 °C / mS cm^{-1}	Lifespan of Zn‖Zn coin cells/ h (mA cm^{-2} / mA h cm^{-2})	Cumulative capacity / Ah cm^{-2}	Ref.
Diethyl carbonate (DEC)	2m $Zn(OTf)_2$ + H_2O/DEC	45% in weight	29.3	750h (5 / 5)	3.75	8
Sulfolane (SL)	3m $Zn(OTf)_2$ + H_2O/SL	63% in weight	10.5	1100h (5 / 5)	5.5	9
Sulfolane (SL)	3m $Zn(OTf)_2$ + H_2O/SL/HAc	50% in weight	12.4	300h (5 / 5)	1.5	10
Dimethyl methylphosphonate (DMMP)	0.5m $Zn(OTf)_2$ + H_2O/DMMP	91% in weight	4.9	2500h (1 / 1)	2.5	11
Ethanol (EtOH)	1m NaOTf + 0.1m $Zn(OTf)_2$ + H_2O/EtOH	95% in weight	10.5	650h (0.5 / 0.5)	0.325	12
Acetonitrile (AN)	1m $Zn(OTf)_2$ + H_2O/AN	78% in weight	29.1	300h (5 / 2.5)	1.5	13
Dimethyl carbonate (DMC)	1m $Zn(OTf)_2$ + H_2O/DMC	56% in weight	25.4	800h (5 / 2.5)	4	14
N,N-Dimethylformamide (DMF)	2M $Zn(OTf)_2$ + H_2O/DMF	33% in volume	/	2000h (4.1 / 4.1)	8.2	15
Propylene carbonate (PC)	Saturated $Zn(OTf)_2$ + H_2O/PC	50% in volume	16	1600h (1 / 1)	1.6	16
Polyethylene glycol dimethyl ether (PEGDME)	2M $Zn(OTf)_2$ + H_2O/PEGDME	30% in volume	40.6	500h (5 / 5)	2.5	17
Triethyl phosphate (TEP)	0.5m $Zn(OTf)_2$ + H_2O/TEP	70% in volume	6.48	600h (1 / 5)	0.6	18

γ-Valerolactone	2m $Zn(OTf)_2$ + H_2O/γ-Valerolactone	65% in weight	41% of the aqueous electrolyte	500h (5 / 5)	2.5	19
2-methoxyethanol (ME)	3m $Zn(OTf)_2$ + H_2O/ME	41% in weight	23.86	2039h (5 / 5)	10.2	This work
1,2-dimethoxyethane (DME)	3m $Zn(OTf)_2$ + H_2O/DME	39% in weight	26.64	2154h (5 / 5)	10.8	This work
**1, 2-diethoxyethane (DEE)**	**3m $Zn(OTf)_2$ + H_2O/DEE**	**11% in weight**	**54.46**	**2800h (5 / 5)**	**14**	**This work**

Supplementary Table 4. Comparison of optimal dosage, molecular weight, Coulombic efficiency, overpotential and lifespan of the DEE-containing LEI-forming electrolyte solution with previously reported electrolytes modified by surfactants. The average CE was obtained from asymmetric Zn||Cu coin cells using glass fibre membranes tested at 25 °C (unless otherwise specified). M = mol L^{-1}; m = mol kg^{-1}.

	Surfactant	Optimal dosage	Molecular weight / g mol^{-1}	Electrolyte solution	Average CE / %	Overpotential / V (1 mA cm^{-2})	Lifespan of Zn‖Zn cells / h (5 mA cm^{-2})	Ref.
1	Benzyltrimethylammonium chloride	0.5 g L^{-1}	185.69	2M $ZnSO_4$	99 (0.5 mA cm^{-2}, 1 mA cm^{-2}, Zn‖Ti)	0.08	500	20
2	Dodecyltrimethylammonium chloride	10 mM	263.89	2M $ZnSO_4$	99.4 (5 mA cm^{-2}, 1 mA cm^{-2})	0.1	500	21
3	n-Octyltrimethylammonium bromide	0.2 M	252.24	2M $ZnSO_4$	98.78 (2 mA cm^{-2}, 1 mA cm^{-2})	0.1	400	22
4	Tween-85	2 g L^{-1}	1840	2M $ZnSO_4$	98.11 (1 mA cm^{-2}, 1 mA cm^{-2}, Zn‖Ti)	0.1	650	23
5	N-octyl-N,N-dimethyl-3-ammonio-1-propanesulfonate	1 mM	335.55	2M $ZnSO_4$	99.2 (2 mA cm^{-2}, 1 mA cm^{-2})	0.1	650	24
6	Betaine	5 m	117.15	2M $ZnSO_4$	99.3 (1 mA cm^{-2}, 1 mA cm^{-2})	0.1 (3 mA cm^{-2})	600	25
7	Alkyl polyglycoside	0.5 v.%	510.62	2M $ZnSO_4$	96.4 (1 mA cm^{-2}, 1 mA cm^{-2})	0.13	300	26
8	Sodium 3,3′-dithiodipropane sulfonate	10 mM	356.42	1M $ZnSO_4$	99.72 (1 mA cm^{-2}, 1 mA cm^{-2})	0.05	900	27
9	Tetrabutylammonium sulfate	0.05 mM	580.99	2M $ZnSO_4$	98 (10 mA cm^{-2}, 10 mA cm^{-2})	0.13	160	28
10	Sodium 3-mercapto-1-propanesulfonate	20 mM	178.21	2M $ZnSO_4$	99.7 (1 mA cm^{-2}, 1 mA cm^{-2})	0.04	980	29
11	Triton X-100 (TX-100)	5 wt.%	625	1m $Zn(OTf)_2$	99.2 (3 mA cm^{-2}, 3 mA cm^{-2})	0.1	/	30

12	Sodium dodecyl sulfate	0.1 g L^{-1}	288.38	2M $ZnSO_4$	99.4 (1 mA cm^{-2}, 0.5 mA cm^{-2})	0.05	520	31
13	Dodecyltrimethyl ammonium chloride	0.05 m	348.48	2m $ZnSO_4$	99.42 (5 mA cm^{-2}, 1 mA cm^{-2})	0.15	1500	21
14	Hexadecyl trimethylammonium bromide	0.5 g L^{-1}	364.45	$Zn(OTf)_2$	98.73 (2 mA cm^{-2}, 1 mA cm^{-2}, Zn‖Ti)	0.04	/	32
15	Sodium dodecyl benzenesulfonate	1000 ppm	348.48	2M $ZnSO_4$	99.5 (2 mA cm^{-2}, 1 mA cm^{-2})	0.08	/	33
16	Tween-80	0.1 mM	1310	2M $ZnSO_4$	/	0.15	700	34
17	Tetraethyl ammonium chloride	0.05 m	165.7	1M $ZnSO_4$	99.1 (1 mA cm^{-2}, 1 mA cm^{-2})	0.14	/	35
18	Sodium methylenedinaphthalene disulphonate (SMD)	80 mM	676.5	2M $ZnSO_4$	99.35 (2 mA cm^{-2}, 1 mA cm^{-2})	0.025	/	36
19	Sodium dodecyl sulfate	/	288.38	1M $Zn(OTf)_2$	98.4 (2 mA cm^{-2}, 1 mA cm^{-2})	0.04	/	37
20	Poloxamer	/	/	2M $ZnSO_4$	99.24 (5 mA cm^{-2}, 2.5 mA cm^{-2}, Zn‖Ti)	0.075	500	38
21	2-(N-morpholino)ethanesulfonic acid	0.05 M	195.24	2M $ZnSO_4$	/	0.05	450	39
22	Potassium polyacrylate	0.2 g L^{-1}	/	2M $ZnSO_4$	99.2 (1 mA cm^{-2}, 1 mA cm^{-2}, filter paper)	0.08	/	40
**23**	**1, 2-diethoxyethane (DEE)**	**1.8 mol%**	**118**	**3m $Zn(OTf)_2$**	**99.83 (1 mA cm^{-2}, 1 mA cm^{-2})**	**0.03**	**2800**	**This work**

Supplementary Table 5. Comparison of cycle performance of aqueous Zn metal-based pouch cells at 25 °C using the DEE-containing LEI-forming electrolyte solution with previously reported electrolyte solutions.

**Electrolyte solution**	**Positive electrode**	**Mass loading / mg cm^{-2}**	**N/P ratio**	**E/C ratio**	**Cell configuration, electrode size and external pressure**	**Specific current**	**Discharge capacity retention (cycle number)**	**Reference**
$Zn(OTf)_2$ in TEP/H_2O binary electrolyte	$Al_xV_2O_5$	10	3	3 mL Ah^{-1}	Multilayered stacking<br>/<br>/	100 mA g^{-1}	83.8<br>(50 cycles, vs. 2nd-cycle capacity)	41
2M $Zn(OTf)_2$ + H_2O/DME (3: 2 in volume)	$V_2O_5·nH_2O$	9.33	2.81	/	Single-layer<br>5 × 6 cm^2<br>/	300 mA g^{-1}	83.5<br>(100 cycles, vs. 1st-cycle capacity)	42
2m $Zn(OTf)_2$ + H_2O/1m 12-C-4 (4: 1 in weight)	$Zn_{0.25}V_2O_5$ nH_2O	50	2.2	8 g Ah^{-1}	Bipolar<br>10.2 × 9.2 cm^2<br>0.1 MPa	44 mA g^{-1}	~65<br>(160 cycles, vs. maximum capacity)	43
2M $Zn(OTf)_2$ + H_2O/HMPA (9: 1 in volume)	V_2O_5	12	2.5	/	Single-layer<br>5 × 6 cm^2<br>/	~83 mA g^{-2}	71.4<br>(300 cycles, vs. 1st-cycle capacity)	44
3m $Zn(OTf)_2$ + H_2O/SL (37: 63 in weight)	$Zn_{0.25}V_2O_5$ nH_2O	20	~3.2	7 mL Ah^{-1}	Bipolar<br>9 × 10 cm^2<br>0.1 MPa	56 mA g^{-1}	80<br>(390 cycles, vs. maximum capacity)	9
1M $Zn(PS)_2$ + 0.2 g L^{-1} TBATS	PANI	1.2	>44	90 mL Ah^{-1}	Single-layer<br>/<br>/	200 mA g^{-1}	93<br>(1000 cycles, vs. maximum capacity)	45

1M $ZnSO_4$ + 0.01M SPC	$V_2O_5·nH_2O$	7	4.23	/	Single-layer<br>3 × 5 cm^2<br>/	~142 mA g^{-1}	82.8<br>(200 cycles, vs. 1st-cycle capacity)	27
2M Li_2SO_4 + 1 M $ZnSO_4$ + 0.5 wt.% ZrP	$Li_2Mn_2O_4$	10	3	/	Single-layer<br>7.5 × 5.5 cm^2<br>/	~74 mA g^{-1}	90<br>(100 cycles, vs. 1st-cycle capacity)	46
2M $ZnSO_4$ + 0.1% N,S-CDs	NaV_3O_8	16.47	1.46	11.7 mL Ah^{-1}	Single-layer<br>6.5 × 8 cm^2<br>/	1000 mA g^{-1}	89.4<br>(120 cycles, vs. 1st-cycle capacity)	47
LiTFSI: $Zn(TFSI)_2$: urea: water = 1: 0.05: 3.8: 2.0	$Li_2Mn_2O_4$	9.6	9.8	/	Multilayered stacking<br>/<br>/	74 mA g^{-1}	84.8<br>(160 cycles, vs. 1st-cycle capacity)	48
30m $ZnCl_2$ + 5m LiCl + 10m TMACl + DMC	$VOPO_4$	22.5	2.3	15 g Ah^{-1}	Single-layer<br>3 × 3 cm^2<br>/	40 mA g^{-1} (First 250 cycles)<br>50 mA g^{-1} (Following 250 cycles)	~73<br>(500 cycles, vs. maximum capacity)	50
**3m $Zn(OTf)_2$ + H_2O/1.8 mol% DEE**	**NaV_3O_8**	**12.5**	**1.61**	**6 g Ah^{-1}**	**Single-layer<br>3 × 4 cm^2<br>0.1 MPa**	**500 mA g^{-1}**	**80<br>(500 cycles, vs. maximum capacity)**	**This work**

Supplementary Table 6. Comparison with state-of-the-art HCEs and OAHEs. All tests were carried out in coin cell configurations at 25°C. The suffixes in the ESW and bulk ionic conductivity at 25 °C columns refer to the reference list and do not denote exponential values.

**Electrolyte solution**	**ESW (V)**	**Bulk ionic conductivity at 25 °C (mS cm^{-1})**	**Cycle stability**		**Rate performance**	
			**Zn||NaV_3O_8 (200 cycle at 500 mA g^{-1})**	**Zn||$LiMn_2O_4$ (100 cycle at 200 mA g^{-1})**	**Zn||NaV_3O_8 (5000 mA g^{-1})**	**Zn||$LiMn_2O_4$ (2000 mA g^{-1})**
**LEI-forming electrolyte**	**3.08**	**54.46**	**96%**	**76%**	**180.2 mAh g^{-1}**	**91.4 mAh g^{-1}**
30m $ZnCl_2$	2.30[51]	8.0[50]	30%	/	54.4 mAh g^{-1}	/
20m LiTFSI + 1m $Zn(TFSI)_2$	~3.11[52]	2.74[53]	81% (100 cycle, short-circuit)	79%	43.8 mAh g^{-1}	31.2 mAh g^{-1}
4m $Zn(BF_4)$ + EG + H_2O	/	4.5[54]	18%	/	29.6 mAh g^{-1}	/
1m $Zn(TFSI)_2$ + SL + H_2O	~3.00[55]	10.00[55]	91%	/	29.3 mAh g^{-1}	/
30m $ZnCl_2$ + 5m LiCl	/	10.00[50]	/	Incomplete charging	/	0 mAh g^{-1}
0.5M $Zn(OTf)_2$ + 1M Li_2SO_4 + TMP + H_2O	/	2.23	/	35%	/	2.3 mAh g^{-1}
$Zn(Ac)_2$ $2H_2O$ + LiAc + Urea	3.50[56]	2.34[56]	/	13%	/	0 mAh g^{-1}

Supplementary Note 1: The correlation between the CEs of Zn||Cu coin cells using the aqueous electrolyte solutions with the organic additives and their physicochemical properties

Statistical analysis was conducted to understand the correlation between coulombic efficiency (CE) of asymmetric Zn||Cu coin cells in electrolyte solutions with selected organic additives and their physicochemical properties, including adsorption ability, hydrophobicity and solvation ability. The concentration of organic additive in the aqueous electrolyte solutions was set at 1.8 mol%. According to these three target properties, four descriptors were identified: contact angle, log $P_{oct/wat}$, donor number (DN), and dielectric constant.

The contact angle (θ) between the Zn foil and various electrolyte solutions correlates with the adsorption of the organic additives on the Zn electrode. In the pure aqueous electrolyte solution, the water molecule is the main adsorbed species on the Zn metal electrode surface, with a contact angle of around 90°. While in organic additive-containing electrolyte solutions, the adsorption of organic molecules can partially replace the adsorbed water molecules and reduce the surface tension at the solid electrode|liquid electrolyte interface, thereby decreasing the contact angle, in accordance with Young's equation [57,58]. For Zn metal, a mildly polar surface with a contact angle around 90°, the change in the contact angle shows a nearly linear correlation to the surface excess number density, that is, the number of adsorbents per unit area of the interface [59]. Therefore, the contact angle between the Zn metal electrode and the electrolyte solution can be a reliable descriptor of the adsorption ability of organic molecules on the Zn metal surface; a smaller contact angle indicates stronger adsorption.

The log $P_{oct/wat}$ of solvents represents their intrinsic hydrophobicity; the higher the value, the stronger the hydrophobicity. A lower DN value indicates weaker Zn^{2+} solvation ability, and a lower dielectric constant relates to a weaker zinc salt-dissociation ability [60]. Three statistical models were utilized to understand the correlation between CE of Zn||Cu coin cells and these four descriptors, including Pearson correlation coefficient (R), mutual information (MI), and permutational feature importance (FI). R, MI, and FI represent a linear correlation, a non-linear correlation, and an important score based on a machine learning algorithm, respectively.

Supplementary Fig. 1 shows the CE of asymmetric Zn||Cu coin cells at 1 mA cm^{-2}, 1 mA h cm^{-2} and 25 °C. Supplementary Fig. 2 presents the contact angles between Zn foil and electrolyte solutions containing various organic additives. Physical properties of the investigated organic additives are reported in Supplementary Table 1. The correlation coefficient between CE and four descriptors are shown in Supplementary Figs. 3 and 4. Among these, the contact angle shows a strong correlation with CE, followed by log $P_{oct/wat}$ with a moderate correlation, while donor number and dielectric constant show a weak correlation. Noted, CE does not exhibit a simple linear correlation with these four descriptors. Instead, organic additives were categorized into three groups based on contact angle parameters, representing their adsorption ability: weak-adsorption solvents (zone I, contact angle > 85°), moderate-adsorption solvents (zone II, 85° > contact angle > 65°), and strong-adsorption solvents (zone III, contact angle < 65°) (Supplementary Fig. 5a). Organic compounds in different zones operate through distinct mechanism to enhance the electrochemical reversibility

of Zn electrode, as evidenced by the CEs (Zone I: CE < 92%, Zone II: 96% < CE < 98.5% and zone III: CE > 98.5%).

In Zone I, the organic additives, characterized by weak adsorption abilities, primarily influence the electrolyte solution at the bulk level. For example, they can stabilize the H_2O molecule by forming strong H-bonds or replace the H_2O molecule in the Zn^{2+} solvation sheath (Supplementary Fig. 5c). This mechanism requires solvents with high polarity and strong solvation capabilities. In this case, to improve electrochemical performance, a substantial amount of zone I solvent is due to the high concentration of water and Zn^{2+} in the bulk electrolyte solution.

Organic additives in Zone II, with moderate adsorption ability, can replace the H_2O molecule in the inner Helmholtz plane (IHP) of the Zn electrode (Supplementary Fig. 5d). Here, the electrochemical performance improvement is favoured when the organic additive exhibit greater hydrophobicity, as evidenced by the strengthened linear correlation between CE and solvent hydrophobicity (R value increases from 0.266 to 0.654, Supplementary Fig. 5b). However, due to their limited adsorption ability and hydrophobicity, a considerable dosage of organic additive in Zone II is needed to achieve satisfactory performance (CE > 99.0%).

For organic additives in the zone III, the electrochemical performance in Zn||Cu coin cell was not only influenced by adsorption ability and hydrophobicity, as suggested by the weak correlation of CE with these two descriptors (Supplementary Figs. 6a and 7). Instead, CE exhibits a nearly linear correlation with the donor number with an R value of −0.968, suggesting the importance of solvation capability for organic additives in zone III. This reflects how the solvation ability of the organic additive affects the compactness of the solvent adsorption layer, specifically the liquid electrolyte interphase (LEI) on the Zn metal electrode. Despite high hydrophobicity, solvents with strong solvation energies tend to solvate with Zn^{2+} ions, dispersing them throughout the bulk solution and forming a less compact adsorption layer with a partially water-expulsing region (Supplementary Fig. 6b). In contrast, weakly solvated organic additives in zone III exist as free molecules in the bulk aqueous electrolyte solution. Driven by hydrophobicity forces, these "free" organic molecules aggregated into clusters similar to solvent-in-water micelles in the water-rich region [61-63]. As such, the adsorption of these organic molecule clusters on the Zn surface forms a compact and continuous LEI that fully covers the Zn surface, creating a compact water-proof region that enhances reduction stability (Supplementary Fig. 6c). According to the contribution of three descriptors, the CE of zone II and zone III solvents can be described by the following equation:

$$CE = -0.127 * x_1 + 0.093 * x_2 - 0.037 * x_3 + 5.074 * \log(x_1) + 0.152 * \log(x_3) + 85.680$$

where x_1 is the contact angle, x_2 the log $P_{oct/wat}$ and x_3 the donor number. This equation was obtained from "Ridge Regression" machine learning algorithm with a regularization parameter (alpha) of 0.01 and can be utilized for the seeking of a more competitive solvent for LEI formation in the future.

The statistical analysis reveals that Zn||Cu CE is correlated with our proposed three descriptors (contact angle, log $P_{oct/wat}$ and DN). To understand the underlying mechanism of

LEI formation, we therefore focus our subsequent analysis on DEE (zone III) and its homologous: 2-methoxyethanol (ME, zone I) and 1,2-dimethoxyethane (DME, zone II). These three ether molecules were chosen due to their same molecular backbone but the incremental amount of $-CH_2-$ group. As the molecular size increases from ME to DME to DEE, their adsorption ability and hydrophobicity increase, while their solvating capability decreases, suggesting an enhanced propensity for LEI formation.

Supplementary Note 2: Evaluation of solvation structure and pH effects in DEE-containing electrolytes

We prove that neither the solvation structure nor the pH accounts for the improved Zn reversibility in the 1.8 mol% DEE electrolyte. Specifically, adding 1.8 mol% DEE does not alter the Zn^{2+} solvation structure in the baseline AE, and the water-solvated Zn^{2+} clusters remain dominant with an average coordination number of 4.99, close to that in AE (5.13) (Supplementary Figs. 48–50). Therefore, no anion-derived SEI layer is formed on Zn cycled in 1.8 mol% DEE. Instead, the surface decomposition products are reduced compared to baseline AE, 1.8 mol% ME- and 1.8 mol% DME- containing electrolytes, as the DEE-derived LEI effectively passivates the Zn surface (Supplementary Figs. 51–53). Furthermore, the initial pH of the 1.8 mol% DEE-containing electrolyte solution (3.47) is almost the same as that of AE (3.53), and neither electrolyte exhibits notable pH-buffering capability, indicating that pH also does not contribute to the improved Zn reversibility (Supplementary Figs. 54 and 55).

Supplementary Note 3: Fundamental differences between surfactant additives and the LEI strategy

Surfactants have been widely used as anti-corrosion agents for metals and electrolyte additives in aqueous zinc batteries (AZBs) to protect the Zn electrode from HER and corrosion. Similar to DEE, surfactants can adsorb onto the Zn surface as single or double molecular layers due to their strong adsorption, passivating the Zn electrode. However, surfactants are typically large molecules with bulky molecular structure (Supplementary Fig. 56a and Table 4), leading to a loose adsorption structure that allows water penetration[64,65]. This compromises their ability to suppress HER, as reflected by their unsatisfactory CE for Zn plating/striping (Supplementary Figs. 56b and 57). Furthermore, their long alkyl chains hinder Zn^{2+} transport (Supplementary Fig. 56c), disrupting the dynamic zinc plating/stripping process and ultimately leading to unstable cycling of the battery (Supplementary Figs. 56d and 57). In contrast, the small molecular size and Zn^{2+} coordination ability of DEE molecules enable the formation of compact and Zn^{2+} conductive LEI by DEE clusters. This LEI effectively blocks water penetration and regulates Zn^{2+} flux, thereby improving zinc plating/stripping CE and extending cell cycle life. These findings further demonstrate that our LEI strategy is fundamentally distinct from the conventional one using surfactant additives.

Supplementary Note 4: Broadness of the LEI design principle

In addition to DEE, we examined the performance of two other organic additives in zone III (Fig. 2a in the main text), namely carbonates (diethyl carbonate (DEC)) and alcohols (1-butanol (n-BuOH)), and compared them with their non-LEI-forming homologous compounds (dimethyl carbonate (DMC); methanol (MeOH) and ethanol (EtOH)). Identifying a low optimal additive concentration is crucial for successful LEI formation. Supplementary Figs. 58 and 59 show that the electrolyte solution using DMC (zone I) exhibits an optimal concentration above 16.2 mol% but achieves a shorter Zn||Zn cells lifespan of 90 hours at 5 mA cm^{-2} and 5 mA h cm^{-2}. In contrast, the DEC-containing electrolyte solution at a much lower concentration of 1.8 mol% achieves a prolonged lifespan of 1500 hours in Zn||Zn cells due to the formation of a passivated DEC-LEI layer. The 1.8 mol% DEC-containing electrolyte also enhances the lifespan of the Zn electrode at a high DoD_{Zn} of 80% in Zn||Zn cell configuration (over 400 hours, Supplementary Fig. 60). Similarly, among the selected three ethanol, *n*-BuOH-containing electrolytes enables better Zn||Zn cell performance (600 hours) with a low dosage of 9.0 mol% *n*-BuOH, while MeOH and EtOH (zone I) enable lifespans of less than 200 hours even at high concentration exceeding 27.0 mol% (Supplementary Figs. 61 and 62). The substantial performance improvement in Zn||Zn cells when using DEC and n-BuOH, compared with their non-LEI-forming homologs, highlights the effectiveness of LEI-based design strategies and our organic additive screening metrics. Notably, both DEC and n-BuOH in zone III demonstrate poorer Zn||Zn cell performance compared to DEE, highlighting the crucial role of solvation ability in LEI formation as outlined for zone III solvents.

Different application scenarios require electrolyte solutions with diverse functionalities, driving the development of electrolytes with varied salt chemistries. Therefore, we expand the broadness of the LEI strategy by using two commonly used salts for preparing battery electrolyte solutions: $ZnSO_4$ and $Zn(ClO_4)_2$. $ZnSO_4$ and $Zn(ClO_4)_2$ are salts with strong salting-out effect (kosmotropes) and salting-in effect (chaotropes), respectively. These salting-in/out effects influence interactions among salt-derived ions, water, and organic solvents, aligning with the Hofmeister series (Supplementary Fig. 63) [66-68]. Most anions fall between SO_4^{2-} and ClO_4^{-}, including OTf^{-}, which exhibits a moderate salting-in effect within $Zn(OTf)_2$.

In contrast to $Zn(OTf)_2$, chaotropic $Zn(ClO4)_2$ exhibits weaker hydration, which facilitates salt dissolution and the dispersion of organic additives with low water miscibility. This requires forming DEE micelles with a compact LEI at a higher DEE dosage. Our finding indicates that the optimized concentration of DEE was increased from 1.8 mol% (using $Zn(OTf)_2$ salt) to 9.0 mol% (using $Zn(ClO_4)_2$ salt), with a Zn||Zn cell lifetime of 1040 hours. However, this performance still exceeds that for the Zn||Zn cells using the aqueous electrolytes with 21.6% mol% ME and 16.2 mol% DME in the $Zn(ClO_4)_2$ salt systems (see Supplementary Figs. 64 and 65). For kosmotropic $ZnSO_4$, its strong hydration capacity reduces the solubility of organic additives, pushing them out of the water phase and forming a separate organic phase (Supplementary Fig. 66). This facilitates DEE micelle formation with an LEI layer on the Zn metal electrode surface at a lower DEE concentration. We observed that the DEE-containing aqueous electrolyte solution at a dosage of 0.18 mol% in the $ZnSO_4$ system shows improved Zn||Zn cell cycling performance (1037 hours) when compared to those electrolyte solutions

with the 1.8 mol% ME (394 hours) and 0.36 mol% DME (616 hours) additives in the $ZnSO_4$ salt systems (see, Supplementary Figs. 67 and 68). Our findings demonstrate the impact of salt chemistry on the formation of the LEI layer, emphasising the wide applicability of our design.

Supplementary Note 5: Formation and reversibility of BZS and their impact on cycling stability of the NaV_3O_8 cathode

It is observed that, for all the electrolyte solutions used, diffraction peaks corresponding to BZS ($Zn_xOTf_y(OH)_{2x-y}$ H_2O), a characteristic by-product of proton insertion, emerge when the cell potential decreases to ~0.8 V (Supplementary Fig. 74), indicating that NaV_3O_8 also follows a Zn^{2+}/H^+ co-intercalation mechanism when these electrolytes are used. These BZS phases are fully reversible during cycling, forming upon discharge and vanishing upon charging, with their peaks disappearing completely upon charging to 1.0 V as the protons are released back into the electrolyte solution, leading to corresponding BZS dissolution. These processes can be described by the following chemical equations:

$$xZn^{2+} + NaV_3O_8 + 2xe^- \leftrightarrow Zn_xNaV_3O_8 \text{ (}Zn^{2+}\text{ (de-)intercalation, above 0.8 V) (1)}$$

$$Zn_xNaV_3O_8 + yH_2O + ye^- \leftrightarrow Zn_xH_yNaV_3O_8 + yOH^- \text{ (}H^+\text{ (de-)intercalation, below 0.8 V) (2)}$$

$$xZn^{2+} + yOTf^- + (2x-y)OH^- + H_2O \leftrightarrow Zn_xOTf_y(OH)_{2x-y}\ H_2O \text{ (BZS formation/dissolution) (3)}$$

This reversible behaviour, which has also been reported for other vanadium-based positive electrode active materials (V_2O_5 and VO_2),[69,70] suggests that BZS formation is not responsible for the capacity fading of Zn||NaV_3O_8 cells.

Supplementary Note 6: Electrochemical characterizations of the electrolyte solutions with high-potential positive electrodes

To verify the ability of the LEI to hinder oxygen evolution reactions, we assembled and tested Li-Zn hybrid cells using high-potential Li-ion intercalation positive electrode active materials, such as $LiMn_2O_4$ and $LiVPO_4F$. These Li^+-intercalation positive electrodes exhibit higher theoretical operating potentials (> 2.0 V vs $Zn|Zn^{2+}$) than most V-based and Mn-based positive electrode active materials used in current AZBs, which typically operate below 2.0 V (vs $Zn|Zn^{2+}$), a value within the ESW of conventional aqueous electrolyte solutions. For these tests, 2 mol kg^{-1} (2 m) LiOTf was added to the Zn-based electrolyte solutions to support Li^+ intercalation/de-intercalation in the positive electrode.

Since the $Zn|Zn^{2+}$ potential is 0.25 V higher than the cathodic limit of the ESW of the DEE-based electrolyte solutions, it reduces the claimed ESW (3.08 V) to ~2.75 V (Supplementary Fig. 80). The charge-discharge tests on $Zn||LiMn_2O_4$ coin cells show that the baseline AE + 2 m LiOTf electrolyte cannot be operated with a cut-off potential over 2.4 V (Supplementary Fig. 81). In contrast, 1.8 mol% DEE + 2 m LiOTf electrolyte allows reversible Li^+ intercalation/de-intercalation up to 2.5 V, confirming the expanded oxidative stability provided by the LEI layer. However, the cell exhibits a CE of 92.35% at an upper cut-off potential of 2.5 V (Supplementary Fig. 81), attributed to the moderate oxidation stability of DEE, which is cathodically stable against Zn metal but not fully stable with high-potential positive electrodes.[71,72] To address this issue, DEE additive was replaced with DEC, a carbonate solvent with higher oxidative stability (6.44 V vs $Li|Li^+$) compared to 4.87 V for DEE.[73] Due to the adequate electrode-philicity, hydrophobicity and weak solvation ability, the DEC can work similarly to DEE in forming a LEI on electrodes. DEMS analysis reveals that 1.8 mol% DEC + 2 m LiOTf exhibits an expanded OER onset potential of 1.92 V (*vs.* Ag|AgCl) (Supplementary Fig. 82), compared to 1.81 V for its DEE counterpart. Using this electrolyte, the CE at 2.5 V improved to 99.07% at an upper cut-off potential of 2.5 V (Supplementary Fig. 81), demonstrating the critical role of solvent selection in optimizing the LEI. Long-term cycling of $Zn||LiMn_2O_4$ coin cells with 1.8 mol% DEC + 2 m LiOTf electrolyte demonstrates 76% specific discharge capacity retention after 100 cycles, which is higher than that obtained using the DEE-based system (Supplementary Fig. 83).

Compared with $LiMn_2O_4$, the Zn metal coin cells with $LiVPO_4F$ exhibit a higher redox platform, suggesting a possible increase in the cell energy content (Supplementary Fig. 84). Using the 1.8 mol% DEC + 2 m LiOTf electrolyte solution, the $Zn||LiVPO_4F$ coin cell enables reversible Li^+ intercalation/de-intercalation up to 2.5 V with an average CE of ~95%. In contrast, the AE-containing coin cell fails to charge above 2.4 V due to water oxidation (Supplementary Fig. 85). The $Zn||LiVPO_4F$ coin cell retains 65% capacity after 50 cycles at 2.5 V and 78.0 mA g^{-1}, with specific discharge capacity decay mainly attributed to vanadium dissolution rather than electrolyte oxidation.[74] This is supported by the comparable cycling stability at 2.2 V and by clear evidence of vanadium deposition on the Zn negative electrode after cycling, as revealed by inductively coupled plasma mass spectrometry (ICP-MS) analysis (Supplementary Fig. 86).

Supplementary Note 7: Comparison of our electrolyte design strategy with conventional HCEs and OAHEs

For practical large-scale applications, an extended ESW alone is not sufficient; rate performance and cycle stability are equally critical. To further benchmark the LEI-forming electrolyte solutions against state-of-the-art electrolytes, we compared their rate capability and cycle performance with those of representative high-concentration electrolytes (HCEs) and organic-aqueous hybrid electrolytes (OAHEs) in Zn||NaV_3O_8 and Zn||$LiMn_2O_4$ coin cells. The 1.8 mol% DEE- and 1.8 mol% DEC- containing LEI-forming electrolyte solutions are used in Zn||NaV_3O_8 and Zn||$LiMn_2O_4$ coin cells, respectively. For HCEs, we choose previously validated electrolyte formulations, such as 30 m $ZnCl_2$ and 20 m LiTFSI + 1 m $Zn(TFSI)_2$. 4 m $Zn(BF_4)$ + ethylene glycol (EG) + H_2O and 1 m $Zn(TFSI)_2$ + sulfolane (SL) + H_2O are selected as state-of-the-art OAHEs electrolytes. These electrolytes exhibit wide ESW for use in high-potential zinc batteries (Supplementary Table 6).[50-56]

Previous works indicate that ionic conductivities of these HCEs and OAHEs are generally below 10.00 mS cm^{-1} at 25 °C, which is lower than that of the 1.8 mol% DEE-containing electrolyte (54.46 mS cm^{-1}), and detrimentally affect the rate performance of batteries (Supplementary Figs. 87 and 88). For instance, the Zn||NaV_3O_8 coin cell using the 1.8 mol% DEE-containing electrolyte solution delivers a specific discharge capacity of 180.2 mAh g^{-1} at 5000 mA g^{-1}, while the Zn||$LiMn_2O_4$ cell maintains 94.1 mAh g^{-1} at 2000 mA g^{-1}, corresponding to 58% and 76% of their respective initial specific discharge capacities at 500 mA g^{-1} and 200 mA g^{-1}, respectively. In contrast, cells using HCEs and OAHEs exhibit poorer rate performance, with specific discharge capacity retention below 30% (e.g., 18% for the cell using the 30 m $ZnCl_2$ at 5000 mA g^{-1}, and 26% for the cell using the 20 m LiTFSI + 1 m $Zn(TFSI)_2$ at 2000 mA g^{-1}). Interestingly, Zn||$LiMn_2O_4$ coin cells with deep eutectic electrolytes ($Zn(Ac)_2$ $2H_2O$ + LiAc + Urea) can only operate at specific currents below 300 mA g^{-1}. These results highlight the improved ability of the LEI-forming electrolyte solution to support both high specific discharge capacity and fast charge-discharge rates.

In terms of cycle stability, representative HCEs and OAHEs enable worse long-term cell cycling performance compared to the LEI-forming electrolyte, often starting with lower initial capacities (Supplementary Figs. 89 and 90). For Zn||NaV_3O_8 coin cells, only the 1 m $Zn(TFSI)_2$ + SL+ H_2O OAHE allows for comparable cell cycling stability to cells with the LEI-forming electrolyte solution, with slightly lower specific discharge capacity retention of 91% versus 96% for the cell with the 1.8 mol% DEE electrolyte after 200 cycles (Supplementary Fig. 89). For Zn||$LiMn_2O_4$ cells, the 30 m $ZnCl_2$ + 5 m LiCl HCE cannot allow for charging up to 2.5 V because Cl^- are oxidized at high potential, generating Cl_2 that corrodes the positive electrode (Supplementary Fig. 91). Other HCEs also enable rapid cell capacity decay due to sluggish electrode kinetics (Supplementary Fig. 90). Among them, only the 20 m LiTFSI + 1 m $Zn(TFSI)_2$ HCE demonstrates stable cyclability, allowing for cells retaining 79% of their initial specific discharge capacity after 100 cycles, which is slightly higher than 76% for the cell with the 1.8 mol% DEC + 2 m LiOTf electrolyte.

The specific energy analysis reveals that Zn||$LiVPO_4F$ coin cell with the 1.8 mol% DEC-containing electrolyte + 2m LiOTf achieves 179 Wh kg^{-1} at 2.5 V, calculated based on the

combined mass of the positive and negative electrodes with an N/P ratio of 1.60. This value is higher than 136 Wh kg^{-1} obtained for the same cell configuration with an upper cut-off potential of 2.2 V (Supplementary Fig. 92). In comparison, the Zn||NaV_3O_8 cell delivers relatively lower specific energy (136 Wh kg^{-1}) due to its low average cell discharge potential. However, it offers excellent stability, cost-effectiveness, and a lithium-free composition, making it equally attractive for grid-scale storage, where long service life, costs, and sustainability aspects are paramount (Supplementary Fig. 93).

References

1 Chao, Y. et al. Recent advancements of electrochemical attenuated total reflection surface-enhanced infrared absorption spectroscopy. *Curr. Opin. Electrochem.* **46**, 101509 (2024).
2 Wang, F. et al. Hybrid aqueous/non-aqueous electrolyte for safe and high-energy Li-ion batteries. *Joule* **2**, 927-937 (2018).
3 Yang, C. et al. 4.0 V aqueous Li-ion batteries. *Joule* **1**, 122-132 (2017).
4 Ma, L. et al. Realizing high zinc reversibility in rechargeable batteries. *Nat. Energy* **5**, 743-749 (2020).
5 Dong, Q. et al. Separators based on the dynamic tip-occupying electrostatic shield effect for dendrite-free lithium-metal batteries. *Adv. Sustain. Syst.* **6**, 2100386 (2021).
6 Li, C. et al. Two-dimensional molecular brush-functionalized porous bilayer composite separators toward ultrastable high-current density lithium metal anodes. *Nat. Commun.* **10**, 1363 (2019).
7 Wang, D. et al. Insight on organic molecules in aqueous Zn-ion batteries with an emphasis on the Zn anode regulation. *Adv. Energy Mater.* **12**, 2102707 (2022).
8 Miao, L. et al. Aqueous electrolytes with hydrophobic organic cosolvents for stabilizing zinc metal anodes. *ACS Nano* **16**, 9667-9678 (2022).
9 Wang, Y. et al. Sulfolane-containing aqueous electrolyte solutions for producing efficient ampere-hour-level zinc metal battery pouch cells. *Nat. Commun.* **14**, 1828 (2023).
10 Zhao, X. et al. Advanced buffering acidic aqueous electrolytes for ultra-long life aqueous zinc-ion batteries. *Small* **18**, e2200742 (2022).
11 Liu, S. et al. Monolithic phosphate interphase for highly reversible and stable Zn metal anode. *Angew. Chem. Int. Ed.* **62**, e202215600 (2023).
12 Sun, Y. et al. Low-cost and long-life Zn/Prussian blue battery using a water-in-ethanol electrolyte with a normal salt concentration. *Energy Storage Mater.* **48**, 192-204 (2022).
13 Zhao, H. et al. Unraveling a cathode/anode compatible electrolyte for high-performance aqueous rechargeable zinc batteries. *Energy Storage Mater.* **50**, 464-472 (2022).
14 Dong, Y. et al. Non-concentrated aqueous electrolytes with organic solvent additives for stable zinc batteries. *Chem. Sci.* **12**, 5843-5852 (2021).
15 Zhou, L. et al. Unshared pair electrons of zincophilic Lewis base enable long-life Zn anodes under "three high" conditions. *Angew. Chem. Int. Ed.* **61**, e202208051 (2022).
16 Ming, F. et al. Co-solvent electrolyte engineering for stable anode-free zinc metal batteries. *J. Am. Chem. Soc.* **144**, 7160-7170 (2022).
17 Yu, M. et al. High-performance zinc-ion battery enabled by tuning the terminal group and chain length of PEO-based oligomers. *Batter. Supercaps* **6**, e202200535 (2023).
18 Liu, S. et al. Tuning the electrolyte solvation structure to suppress cathode dissolution, water reactivity, and Zn dendrite growth in zinc-ion batteries. *Adv. Funct. Mater.* **31**, 2104281 (2021).
19 Xie, C. et al. Weak solvent chemistry enables stable aqueous zinc metal batteries over a wide temperature range from -50 to 80 degrees. *Sci. Bull.* **68**, 1531-1539 (2023).
20 Guan, K. et al. Anti-corrosion for reversible zinc anode via a hydrophobic interface in aqueous zinc batteries. *Adv. Energy Mater.* **12**, 2103557 (2022).
21 Zhang, X. et al. Zincophilic and hydrophobic groups of surfactant-type electrolyte additive enabled stable anode/electrolyte interface toward long-lifespan aqueous zinc ion batteries. *Energy Storage Mater.* **70**, 103500 (2024).

22 Liu, Z. et al. Low‑cost multi‑function electrolyte additive enabling highly stable interfacial chemical environment for highly reversible aqueous zinc ion batteries. *Adv. Funct. Mater.* **33**, 2308463 (2023).
23 Yan, X. et al. Highly reversible Zn anodes through a hydrophobic interface formed by electrolyte additive. *Nanomater.* **13**, 1547 (2023).
24 Tao, L. et al. Dual-protected zinc anodes for long-life aqueous zinc ion battery with bifunctional interface constructed by zwitterionic surfactants. *Energy Storage Mater.* **63**, 102981 (2023).
25 Li, X. et al. Promoting desolvation by hydrophobic and zincophilic adsorption layer to achieve stable Zn anodes at low temperature. *ACS Sustain. Chem. Eng.* **12**, 7858-7868 (2024).
26 Wang, H. et al. Rearrangement of H-bonds network of solvation structure via a zincophilic polyol-type surfactant to stabilize zinc anode in aqueous zinc-ion batteries. *Energy Storage Mater.* **67**, 103238 (2024).
27 Lin, Y. et al. Dendrite-free Zn anode enabled by anionic surfactant-induced horizontal growth for highly-stable aqueous Zn-ion pouch cells. *Energy Environ. Sci.* **16**, 687-697 (2023).
28 Bayaguud, A., Luo, X., Fu, Y. & Zhu, C. Cationic surfactant-type electrolyte additive enables three-dimensional dendrite-free zinc anode for stable zinc-ion batteries. *ACS Energy Lett.* **5**, 3012-3020 (2020).
29 Lin, Y., Li, Y., Mai, Z., Yang, G. & Wang, C. Interfacial regulation via anionic surfactant electrolyte additive promotes stable (002)‑textured zinc anodes at high depth of discharge. *Adv. Energy Mater.* **13**, 2301999 (2023).
30 Zhang, Y. et al. Nonionic surfactant-assisted in situ generation of stable passivation protective layer for highly stable aqueous Zn metal anodes. *Nano Lett.* **22**, 8574-8583 (2022).
31 Jing, F. et al. Interface engineering enabled by sodium dodecyl sulfonate surfactant for stable Zn metal batteries. *J. Colloid. Interface Sci.* **669**, 984-991 (2024).
32 Luo, M. et al. A dendrite suppression coating formulated via electrophoretic deposition using Bi-functional surfactants for Zn-ion batteries. *J. Alloys Compd.* **918**, 165790 (2022).
33 Xie, W. et al. Highly 002-oriented dendrite-free anode achieved by enhanced interfacial electrostatic adsorption for aqueous zinc-ion batteries. *ACS Nano* **18**, 21184-21197 (2024).
34 Thieu, N. A. et al. Synergistically stabilizing zinc anodes by molybdenum dioxide coating and Tween 80 electrolyte additive for high-performance aqueous zinc-ion batteries. *ACS Appl. Mater. Interfaces* **15**, 55570-55586 (2023).
35 Fang, T. et al. Dendrite-free and stable zinc-ion batteries enabled by a cation-anion synergistic regulation additive. *J. Power Sources* **581**, 233521 (2023).
36 Zheng, S. et al. In-situ formation of heterogeneous interfaces inducing surface crystallographic manipulation toward highly stable Zn anode. *Chem. Eng. J.* **473**, 145313 (2023).
37 Guo, G. et al. Zincophilic anionic hydrogel electrolyte with interfacial specific adsorption of solvation structures for durable zinc ion hybrid supercapacitors. *Adv. Funct. Mater.* **34**, 2308405 (2023).
38 Yang, K. et al. Poloxamer pre-solvation sheath ion encapsulation strategy for zinc anode–electrolyte interfaces. *ACS Energy Lett.* **9**, 209-217 (2023).
39 Liu, X. et al. A multifunctional zwitterion electrolyte additive for highly reversible zinc metal anode. *Small* **20**, 2307557 (2024).

40 Zhao, K. et al. Boosting the reversibility of Zn anodes via synergistic cation-anion interface adsorption with addition of multifunctional potassium polyacrylate. *J. Colloid. Interface Sci.* **664**, 816-823 (2024).
41 Zhu, J. et al. The construction of binary phase electrolyte interface for highly stable zinc anodes. *Adv. Mater.*, e2304426 (2023).
42 Ma, G. et al. Reshaping the electrolyte structure and interface chemistry for stable aqueous zinc batteries. *Energy Storage Mater.* **47**, 203-210 (2022).
43 Wang, Y. et al. Manipulating electric double layer adsorption for stable solid‐electrolyte interphase in 2.3 Ah Zn‐pouch cells. *Angew. Chem. Int. Ed.* **135**, e202302583 (2023).
44 Wang, D. et al. Solvation modulation enhances anion-derived solid electrolyte interphase for deep cycling of aqueous zinc metal batteries. *Angew. Chem. Int. Ed.* **62**, e20231029 (2023).
45 Chen, S. et al. Coordination modulation of hydrated zinc ions to enhance redox reversibility of zinc batteries. *Nat. Commun.* **14**, 3526 (2023).
46 Peng, H. et al. Dynamic Zn/electrolyte interphase and enhanced cation transfer of sol electrolyte for all-climate aqueous zinc metal batteries. *Angew. Chem. Int. Ed.* **62**, e202308068 (2023).
47 Xu, Z. et al. Durable modulation of Zn(002) plane deposition via reproducible zincophilic carbon quantum dots towards low N/P ratio zinc-ion batteries. *Mater. Horiz.* **10**, 3680-3693 (2023).
48 Zhao, J. et al. "Water-in-deep eutectic solvent" electrolytes enable zinc metal anodes for rechargeable aqueous batteries. *Nano Energy* **57**, 625-634 (2019).
49 Dong, D., Wang, T., Sun, Y., Fan, J. & Lu, Y.-C. Hydrotropic solubilization of zinc acetates for sustainable aqueous battery electrolytes. *Nat. Sustain.* **6**, 1474-1484 (2023).
50 Jiang, H. et al. Chloride electrolyte enabled practical zinc metal battery with a near-unity Coulombic efficiency. *Nat. Sustain.* **6**, 806-815 (2023).
51 Zhang, C. et al. A $ZnCl_2$ water-in-salt electrolyte for a reversible Zn metal anode. *Chem. Commun.* **54**, 14097-14099 (2018).
52 Lu, H., Zheng, S., Wei, L., Zhang, X. & Guo, X. Manipulating Zn^{2+} solvation environment in poly(propylene glycol)‐based aqueous Li^+/Zn^{2+} electrolytes for high‐voltage hybrid ion batteries. *Carbon Energy* **5** (2023).
53 Li, X. et al. Initiating a high-temperature zinc ion battery through a triazolium-based ionic liquid. *RSC Adv.* **12**, 8394-8403 (2022).
54 Han, D. et al. A non-flammable hydrous organic electrolyte for sustainable zinc batteries. *Nat. Sustain.* **5**, 205-213 (2021).
55 Li, M. et al. Comprehensive H_2O molecules regulation via deep eutectic solvents for ultra-stable zinc metal anode. *Angew. Chem. Int. Ed.* **135** (2023).
56 Deng, Y. et al. Inhibition of side reactions and dendrite growth using a low-cost and non-flammable eutectic electrolyte for high-voltage and super-stable zinc hybrid batteries. *J. Mater. Chem. A* **11**, 8368-8379 (2023).
57 Thiele, U., Snoeijer, J. H., Trinschek, S. & John, K. Equilibrium contact angle and adsorption layer properties with surfactants. *Langmuir* **34**, 7210-7221 (2018).
58 Gau, C.-S. & Zografi, G. Relationships between adsorption and wetting of surfactant solutions. *J. Colloid Interface Sci.* **140**, 1-9 (1990).
59 Staniscia, F., Guzman, H. V. & Kanduc, M. Tuning contact angles of aqueous droplets on hydrophilic and hydrophobic surfaces by surfactants. *J. Phys. Chem. B.* **126**, 3374-3384 (2022).
60 Xu, J. et al. Electrolyte design for Li-ion batteries under extreme operating conditions. *Nature* **614**, 694-700 (2023).

61 Sinibaldi, R. et al. The role of water coordination in binary mixtures. A study of two model amphiphilic molecules in aqueous solutions by molecular dynamics and NMR. *J. Phys. Chem. B* **110**, 8885-8892 (2006).
62 Stirnemann, G., Sterpone, F. & Laage, D. Dynamics of water in concentrated solutions of amphiphiles: key roles of local structure and aggregation. *J. Phys. Chem. B* **115**, 3254-3262 (2011).
63 Troncoso, J., Zemánková, K. & Jover, A. Dynamic light scattering study of aggregation in aqueous solutions of five amphiphiles. *J. Mol. Liq.* **241**, 525-529 (2017).
64 Li, J., Yan, G., Zhou, L., Bai, X. & Chen, X. Molecular mechanism of the effect of benzene ring structure in nonionic surfactants on the wettability of anthracite. *Colloids Surf. A: Physicochem. Eng. Asp.* **657**, 130634 (2023).
65 Chen, X. et al. Influence of the branched structure of polyoxyethylene units in nonionic surfactants on the wettability of anthracite: A combined modeling and experimental study. *Adsorp. Sci. Techno.* **2022**, 2022 (2022).
66 He, X. et al. Chaotropic monovalent anion - induced rectification inversion at nanopipettes modified by polyimidazolium brushes. *Angew. Chem. Int. Ed.* **130**, 4680-4683 (2018).
67 Roberts, J. M., Diaz, A. R., Fortin, D. T., Friedle, J. M. & Piper, S. D. Influence of the hofmeister series on the retention of amines in reversed-phase liquid chromatography. *Anal. Chem.* **74**, 4927-4932 (2002).
68 Reber, D., Grissa, R., Becker, M., Kühnel, R. S. & Battaglia, C. Anion selection criteria for water-in-salt electrolytes. *Adv. Energy Mater.* **11**, 2002913 (2020).
69 Kim, Y. et al. Corrosion as the origin of limited lifetime of vanadium oxide-based aqueous zinc ion batteries. *Nat. Commun.* **13**, 2371 (2022).
70 Zhu, K. et al. Synergistic H^+/Zn^{2+} dual ion insertion mechanism in high-capacity and ultra-stable hydrated VO_2 cathode for aqueous Zn-ion batteries. *Energy Storage Mater.* **29**, 60-70 (2020).
71 Li, Z. et al. Non-polar ether-based electrolyte solutions for stable high-voltage non-aqueous lithium metal batteries. *Nat. Commun.* **14**, 868 (2023).
72 Choi, I. R. et al. Asymmetric ether solvents for high-rate lithium metal batteries. *Nat. Energy* **10**, 365-379 (2025).
73 Wang, D. et al. A thermodynamic cycle-based electrochemical windows database of 308 electrolyte solvents for rechargeable batteries. *Adv. Funct. Mater.* **33** (2023).
74 Liao, M. et al. VPO_4F fluorophosphates polyanion cathodes for high-voltage proton storage. *Angew. Chem. Int. Ed.* **134** (2022).
